# Congenital heart defect repair with ADAPT tissue engineered pericardium scaffold: An early-stage health economic model

**DOI:** 10.1371/journal.pone.0204643

**Published:** 2018-09-27

**Authors:** Vladica M. Veličković, Oleg Borisenko, Mikael Svensson, Tim Spelman, Uwe Siebert

**Affiliations:** 1 Synergus AB, Health Economics and Evidence Synthesis Department, Stockholm, Sweden; 2 Institute of Public Health, Medical Decision Making and Health Technology Assessment, Department of Public Health, Health Services Reseaech and Health Technology Assessment, UMIT - University for Health Sciences, Medical Informatics and Technology, Hall i.T., Austria; 3 Health Metrics, The Sahlgrenska Academy, University of Gothenburg, Gothenburg, Sweden; 4 Centre for Population Health, Burnet Institute, Melbourne, Australia; 5 School of Public Health and Preventive Medicine, Monash University, Melbourne, Australia; York University, CANADA

## Abstract

**Objective:**

The objective of this study was to evaluate the cost effectiveness of tissue engineered bovine tissue pericardium scaffold (CardioCel) for the repair of congenital heart defects in comparison with surgery using xenogeneic, autologous, and synthetic patches over a 40-year time horizon from the perspective of the UK National Health Service.

**Methods:**

A six-state Markov state-transition model to model natural history of disease and difference in the interventional effect of surgeries depending on patch type implanted. Patches differed regarding their probability of re-operation due to patch calcification, based on a systematic literature review. Transition probabilities were based on the published literature, other clinical inputs were based on UK registry data, and cost data were based on UK sources and the published literature. Incremental cost-effectiveness ratio (ICER) was determined as incremental costs per quality adjusted life years (QALY) gained. We used a 40-year analytic time-horizon and adopted the payer perspective. Comprehensive sensitivity analyses were performed.

**Results:**

According to the model predictions, CardioCel was associated with reduced incidence of re-operation, increased QALY, and costs savings compared to all other patches. Cost savings were greatest compared to synthetic patches. Estimated cost savings associated with CardioCel were greatest within atrioventricular septal defect repair and lowest for ventricular septal defect repair. Based on our model, CardioCel relative risk for re-operations is 0.938, 0.956and 0.902 relative to xenogeneic, autologous, and synthetic patches, respectively.

**Conclusion:**

CardioCel was estimated to increase health benefits and save cost when used during surgery for congenital heart defects instead of other patches.

## Introduction

Congenital heart defects (CHD) are associated with considerable morbidity and mortality globally, with an incidence ranging from 8 to 13 per 1000 live births [1–3]. The ongoing need for repeat and revision surgery coupled with a lifelong burden of associated disease and comorbidity translates into significant public health impact and healthcare costs, which characteristically extend well beyond childhood. Of the $2.6 billion USD in hospital costs associated with birth defects in the United States during 2004, $1.4 billion (54%) was directly associated with the management and treatment of structural cardiovascular defects [2]. The costs of identifying critical congenital heart defects in neonates have previously been reported at just over $20,000 USD per newborn detected [4, 5] whilst median palliation costs have been reported at up to $99,000 USD [6].

A diverse range of cardiovascular patches for the correction of congenital defects in neonates and pediatric patients have been tested in clinical trials including the use of synthetic, autologous, and biological material [7–12]. Of the biologics, patches derived from bovine pericardium have previously demonstrated to be effective for the closure of several common anomalies [7, 8, 10]. Whilst the complication rate associated with such procedures is relatively moderate [13], calcification of patches with subsequent dehiscence and failure is a known risk of biologics [14], in addition to infection, inflammation, and bleeding [15].

The ADAPT tissue-engineered bovine pericardium bio-scaffold (CardioCel) is distinct from comparable patches by the removal of calcium-binding phospholipids and antigens during manufacture, both of which are known to promote the calcification observed with other patches [13, 16]. Recent data from the extension study of the Phase II trial of CardioCel in CHD repair reported no calcification or repeat surgery across 14 patients followed-up for between five and eight years [unpublished data, [17]]. A single-centre, prospective cohort study of 30 pediatric patients receiving the CardioCel scaffold observed no patch-associated morbidity within one month of insertion and no echocardiographic evidence of calcification, bleeding, or failure at 18 to 36 months’ post-surgery [13]. A recent retrospective German review of 37 pediatric and adult patients similarly reported no patch-related complication or signs of tissue failure [18]. In addition, Prabhu et al. [19] in histologic evaluation of explanted CardioCel in CHD population confirms that in top of the fact that calcification did not occur, CardioCel demonstrate evidence of remodelling and neointima formation.

Whilst the early data suggests a potential advantage favoring CardioCel in terms of calcification, remodeling, and thrombus formation relative to synthetic patches, and lack of calcification, surface thickening, and structural leak relative to biological patches [15], the potential cost-effectiveness of CardioCel has not yet been investigated. The objective of this study was to evaluate the potential cost-effectiveness of CardioCel repair of congenital cardiac defects in comparison with xenogeneic, autologous, and synthetic patches.

## Materials & methods

### Model description

**Fig 1:**
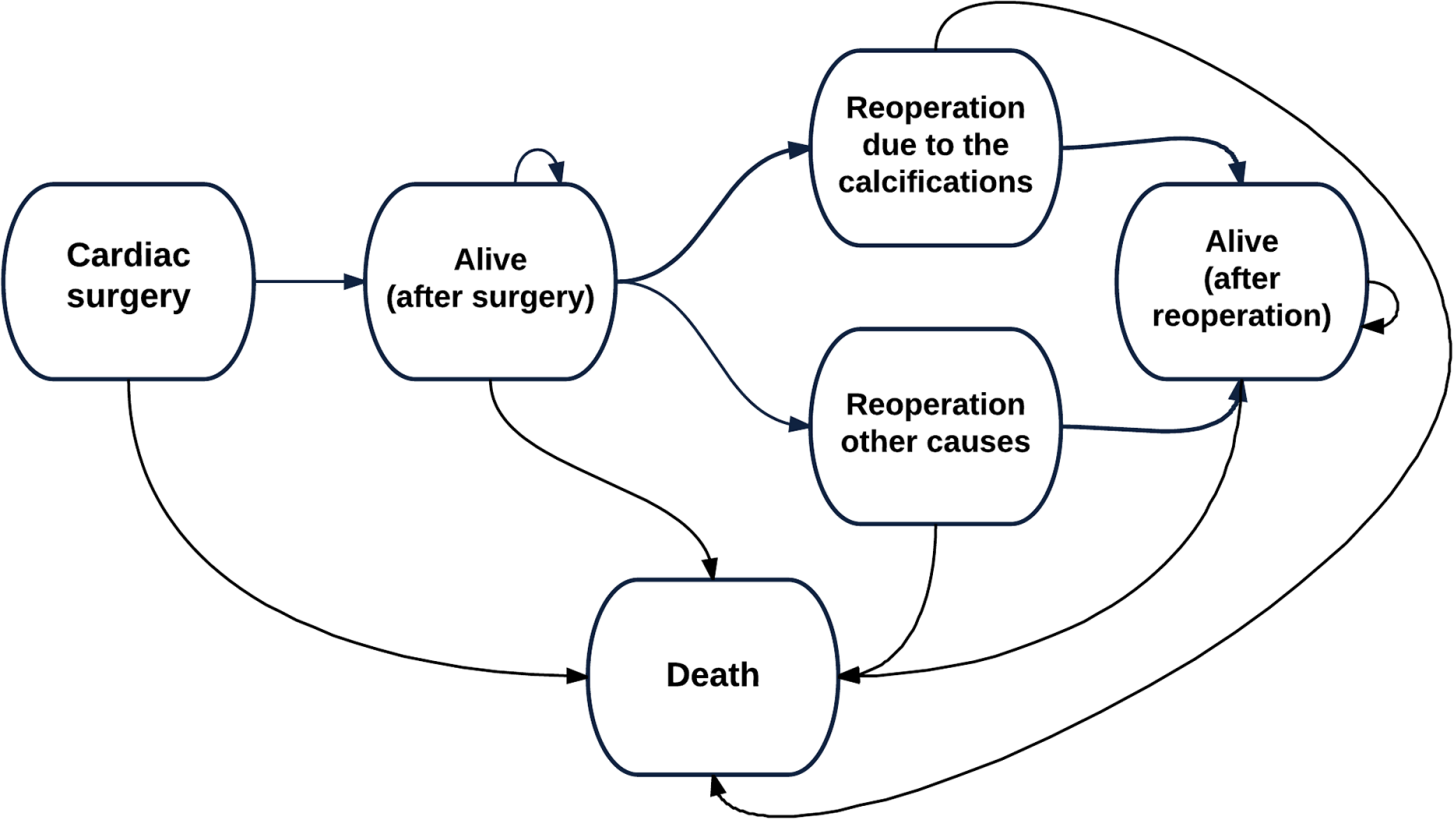
Health states and pathways used in the decision analysis.

A Markov state-transition cohort model was developed and used to estimate the cost-effectiveness of CardioCel versus other patches (Fig 1). Patients enter the model at the time of the index patch repair surgery, after which they may stay alive with no re-operation, undergo repeat operation due to calcification of patch or other causes, or die.

The premise of the analysis is that due to better tissue properties, CardioCel can reduce the frequency of reoperations caused by calcification and correspondingly can increase survival and quality of life of patients with a lower cost to the health care system.

The ideal solution which can provide a full insight into outcomes after patch implantation will be to follow patients from index surgery through the complete lifetime horizon with all relevant events properly analyzed. However, in real world settings this is not feasible both for organisational and financial reasons. On the other hand, the simulation methods framework allows for simulating the lives of virtual patients from the index event until death taking into account all relavant events and synthesize all relevant available evidence (prospective and retrospective primary data, published secondary data sources, and real-world data sources from registries). One of the most frequently used methods for this purpose is the Markov state-transition model, and in line with methodological recommendations we use this method in order to adequately address the research question [20, 21]. We also followed the international ISPOR-SMDM guidelines on decision-analytic modelling [22]. The evaluation is reported in line with “Consolidated Health Economic Evaluation Reporting Standards” (CHEERS) [23].

### Clinical inputs

The target population includes a mix of pediatric patients with six congenital cardiovascular anomalies, treated with the most common procedures involving the use of patches (Table 1). Distribution of surgical methods was informed by the 2014–15 Admitted Care Hospital Episode Statistics (HES), which collects data from all hospital admissions in England [24].

**Table 1:** Congenital heart defects and most common surgical procedures involving use of patches.

Congenital heart defect	Surgical procedure
Aortic valve stenosis	Aortic valvotomy
Transposition of great arteries	Arterial switch
Atrioventricular septal defect	AVSD (complete) repair
Coarctation of the aorta	Isolated cortication repair
Tetralogy of Fallot	Tetralogy repair
Ventricular septal defect	VSD repair

AVSD—Atrioventricular septal defect, ToF—Tetralogy of Fallot, VSD—Ventricular septal defect

Three groups of comparator patches were used: xenogeneic, autologous, and synthetic patches. Synthetic patches included expanded polytetrafluoroethylene (ePTFE) and fabric patches, whilst both decellularized xenogeneic and tissue-engineered patches were considered in the xenogeneic patch group. Utilization of each patch type for different surgeries was based on the 15-year analysis of HES [24].

The three-tier approach was taken in relation to the estimation of the frequency of reoperations. Firstly, disease-specific freedom from re-operation was informed from Monro et al. [25] and Sakurai et al [26]. Secondly, the disease-specific and patch-related etiological fraction of reoperations was estimated via the single source (tetralogy of Fallot (ToF) and coarctation of the aorta (CoA)) or meta-analysis of large, international studies reporting the cause of reoperation (atrioventricular septal defect (AVSD), ventricular septal defect (VSD), and transposition of great arteries (TGA)). Thirdly, within a fraction of patch-related reoperations, the fraction of reoperations due to patch calcification was estimated from explanted patch histological studies. Only patch calcification-related reoperations differ between compared patches. In the case of aortic stenosis, the assumption was made that 100% of reoperation were patch-related due to the specific position and active role of the patch. The baseline value of risk of re-operations in the absence of calcifications were estimated by adjusting these patch-related reoperations

Survival analysis involved calculating of the age-, gender- and disease-specific survival, and additional adjustment for procedure-specific 30-day mortality for both index and redo surgeries. The UK life tables [27] were utilised as a starting point with several subsequent adjustments in order to inform survival in the model. Conceptual steps of that adjustment are presented in Table 2.

**Table 2:** Approach to inform survival in the model.

Baseline mortality	Adjustment 1	Adjustment 2	Adjustment 3
UK life tables	Congenital heart defects relative risk	Disease specific relative risk	Disease specific (re)operative mortality

The first relative risk between mortality of the normal population and patients with CHD was informed from a population-based study from Finland [28]. This study is selected as the best possible source for several reasons: (i) this is the one of the largest CHD patient population-based studies internationally; (ii) reporting survival starting from the index surgery for four surgical decades (this is particularly important due to the significant differences in survival in recent decades); (iii) reported follow-up survival time is from 20 years for the more recent surgical decade up to 45 for oldest one (this give a unique opportunity to use older surgical decades as an external validation source when extrapolating recent surgical decades from 20 years of observed follow-up to 40 years after index surgery). The data from Fig 2 from Nieminen et al. [28] were extracted in order to estimate the relative risk between the general population and CHD disease mortality. In the first step, this relative risk was applied to the UK life tables and average survival for the UK population with CHD was estimated. Additionally, by extracting data from the survival curves in Fig 3 from Nieminen et al [28] the disease specific relative risk was determined as the ratio between average CHD mortality and specific disease mortality. However, due to the limitation of observed survival in the most recent surgical decade of 20 years, after extraction from the original source, data are first extrapolated up to 40 years after index surgery and then the relative ratio was estimated. Exceptions are aortic stenosis and atrioventricular septal defect because they are not reported in Nieminen et al [28] and therefore an alternative source is used for survival estimation. Aortic stenosis survival was informed by Alexiou et al. [29] as a most appropriate available source from the UK reporting survival 25 years after index surgery. In the case of atrioventricular septal defect currently, there is a lack of studies with sufficient follow-up coming from the UK. Therefore, as a most appropriate source, but still relevant for the UK, Ginde et al [30] reporting survival of patients with atrioventricular septal defect after index surgery in the US was selected. Details regarding extrapolation of survival data are reported in the Figs A—I and Tables A—E in S3 File).

**Fig 2:**
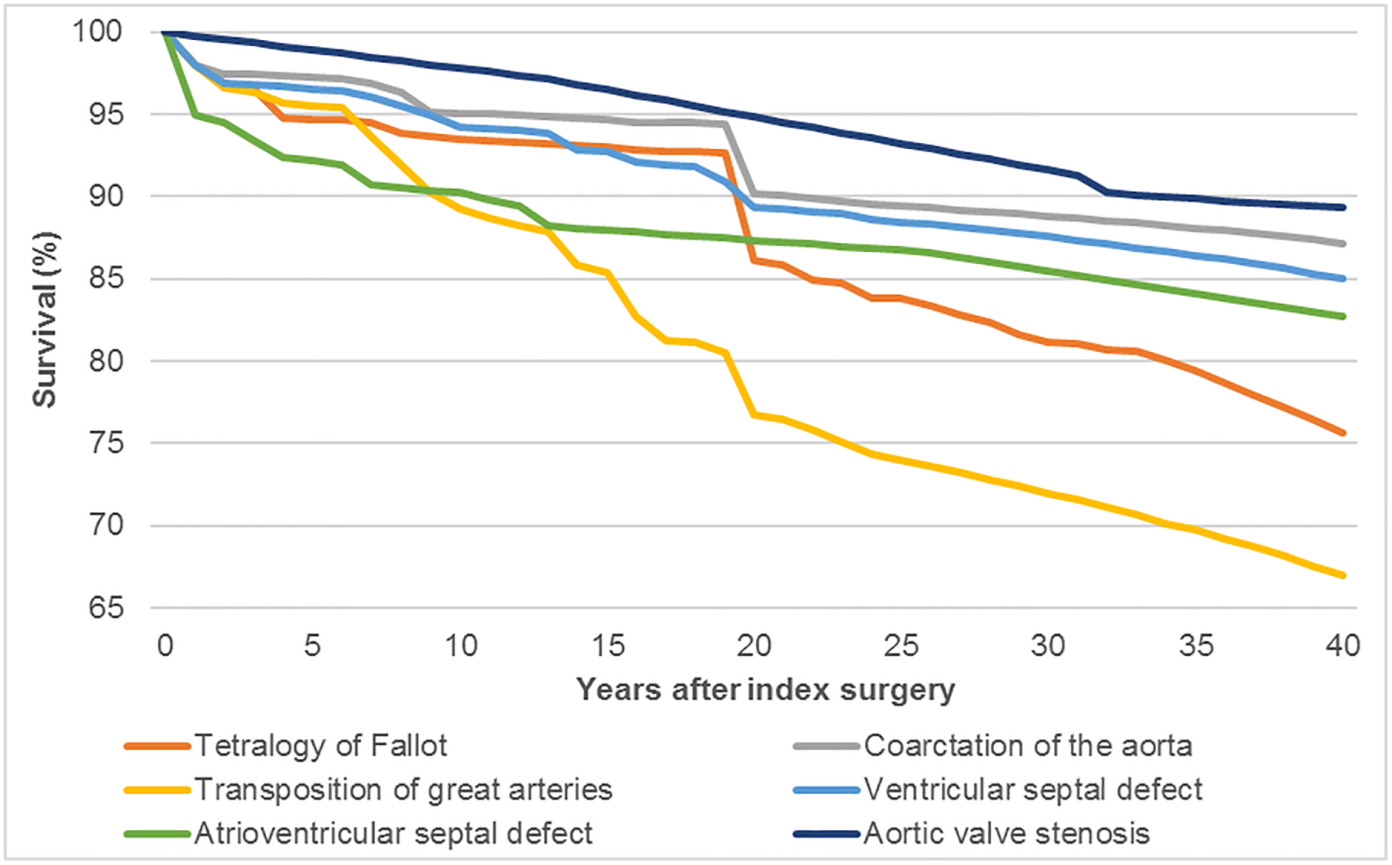
Observed and extrapolated survival. CHD—congenital heart defects, AS—aortic valve stenosis, VSD—ventricular septal defect, AVSD—atrioventricular septal defect, ToF—tetralogy of Fallot, TGA—transposition of great arteries, CoA—coarctation of the aorta.

**Fig 3:**
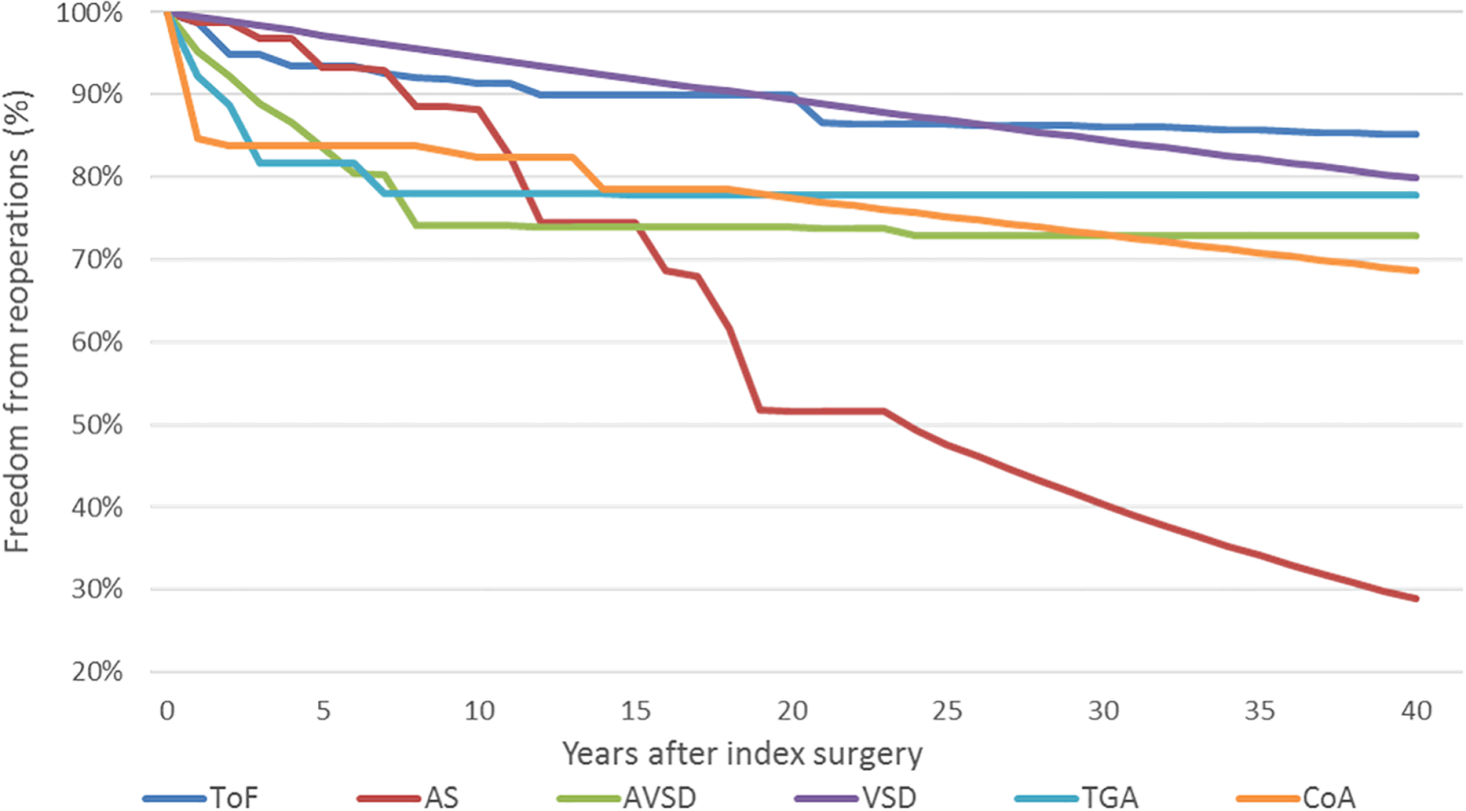
Observed and extrapolated freedom from reoperation data inputs. CHD—congenital heart defects, AS—aortic valve stenosis, VSD—ventricular septal defect, AVSD—atrioventricular septal defect, ToF—tetralogy of Fallot, TGA—transposition of great arteries, CoA—coarctation of the aorta.

The approach in the selection of an extrapolation model was done systematically, similar to the case of reoperation extrapolations and in line with current NICE recommendations [31]. Furthermore, survival in the model was additionally adjusted for procedure-specific operation mortality (30-day mortality) informed by the UK Central Cardiac Audit Database [32] as presented in Table 3.

**Table 3:** Procedure-specific operation mortality (30-day mortality).

Surgical procedure	Operation mortality (%)	Low burden (%)	Upper burden (%)
Aortic valvotomy	7.2	4.2	12.7
Arterial switch	2.4	1.6	3.5
AVSD (complete) repair	2.2	1.5	3.3
Interrupted aortic arch repair	7.7	4.8	12.5
Isolated cortication repair	1.0	0.6	1.6
Norwood procedure	17.5	14.9	20.2
Tetralogy repair	1.6	1.1	2.3
VSD repair	0.9	0.6	1.3

AVSD—Atrioventricular septal defect, VSD—Ventricular septal defect

Re-operation and survival trends (Table 4) were then extrapolated out to a 40-year time horizon using a variety of parametric and non-parametric functions (Figs 2 and 3). Model fit was assessed through comparison of Akaike (AIC) and Bayesian (BIC) Information Criterion, coefficients of determination, visual inspection of residuals, and comparison against external benchmarks [31].

**Table 4:** Clinical parameters.

Model variable	Congenital heart disease (surgical procedure)						Reference
	AS (Aortic valvotomy)	VSD (Isolated repair)	AVSD (Complete repair)	ToF (Tetralogy repair)	TGA (Arterial switch)	CoA (Isolated repair)	
Proportion of patch-related reoperations (fixed values)	16% ^a^ **	16% ^b^	90% ^b^	41% ^c^	53% ^b^	30% ^d^	^a^Assumption; ^b^Meta-analyses (Fig C, E, G in u S1 File);^c,d^Estimation based on HES [24]
Proportion of xenogeneic patches (index surgery)	61%	53%	69%	1%	37%	37%	HES, Admitted Care, 2014–2015[24]
Proportion of autologous patches (index surgery)	9%	23%	16%	54%	31%	31%	
Proportion of synthetic patches (index surgery)	29%	23%	16%	45%	31%	31%	
Calcification incidence CardioCel	5%*	5%*	5%*	5%*	5%*	5%*	Estimated based on Prabhu et al. [19], and Neethling et al. [13]
Calcification incidence xenogeneic patches	18.2%	18.2%	18.2%	18.2%	18.2%	18.2%	Meta-analyses (Table B in S1 File)
Calcification incidence autologous patches	12.5%.	12.5%.	12.5%.	12.5%.	12.5%.	12.5%.	Estimated based on Majeed et al [33]
Calcification incidence synthetic patches	35%	35%	35%	35%	35%	35%	Meta-analyses (Table B in S1 File)
Short-term survival (30-day postoperative period)	7.2%	0.9%	2.2%	1.6%	2.4%	1.0%	UK Central Cardiac Audit Database [32]
Proportion of the patients per indication	0.9%^a^	52	12.9	14.9	10.8	8.3	HES, Admitted Care, 2014–2015[24], ^a^ Proxy value

*Although currently available literature does not report any calcification of the CardioCel scaffold, the analysis used conservative assumption of 5% (range 0–10%) due to the reason that longest follow up data for CardioCel are eight years.

** Due to the lack of literature based inputs, conservative assumption of 16% of patch related reoperations was used in the analysis (equal to lowest value in the overall CHD cohort)

AS—aortic valve stenosis; VSD—ventricular septal defect; AVSD—atrioventricular septal defect; ToF—tetralogy of Fallot; TGA—transposition of great arteries; CoA—coarctation of the aorta; HES—Hospital Episode Statistics

### Utilities

The utilities employed in the analysis were calculated using the approach described by Mistry et al [34] and Knowles [35]. Quality-adjusted life-years (QALYs) were estimated in two steps. In the first step, patients with different CHD types were classified into four different heart failure categories as presented in Table 5.

**Table 5:** Classification of CHD patients into heart failure categories.

Class	Patient Symptoms (NYHA)	CHD disease
Class I (Mild)	No limitation of physical activity. Ordinary physical activity does not cause undue fatigue, palpitation, or dyspnea (shortness of breath)	No need for surgery
Class II (Mild)	Slight limitation of physical activity. Comfortable at rest, but ordinary physical activity results in fatigue, palpitation, or dyspnea	Isolated aortic valve disease, Isolated small ventricular septal defect
Class III (Moderate)	Marked limitation of physical activity. Comfortable at rest, but less than ordinary activity causes fatigue, palpitation, or dyspnea	Atrioventricular septal defects, Coarctation of the aorta, Interrupted aortic arch, Tetralogy of Fallot
Class IV (Severe)	Unable to carry out any physical activity without discomfort. Symptoms of cardiac insufficiency at rest. If any physical activity is undertaken, discomfort is increased	Transposition of the Great Arteries, Hypoplastic left heart syndrome

CHD—congenital heart defects, NYHA—New York Heart Association Functional Classification

In the second step, age-dependent utility values were assigned to the corresponding group of heart failure patients (Table 6.)

**Table 6:** Proxy utility values attached to different age group in patients with CHD diseases.

Age category	Utility values (SE)		References
**Mild CHD disability for the child (Class I and II)**			
0 to 25 years		0.850	Brown et al. [36]
26 to 45 years		0.834 (0.02705)	Kirsch et al. [37]
46 to 65 years		0.697 (0.03306)	
**Moderate CHD disability for the child (Class III)**			
0 to 25 years		0.750 (0.03962)	Yount et al. [38]
26 to 45 years		0.531 (0.06311)	Kirsch et al. [37]
46 to 65 years		0.488 (0.06170)	
**Severe CHD disability for the child (Class IV)**			
0 to 2 years		0.400	Caviness te al. [39]
3 to 18 years		0.390	Brown et al. [36]
19 to 25 years		0.390	Assumption based on Brown et al [36]
26 to 45 years		0.323 (0.06505)	Kirsch et al. [37]

CHD—congenital heart defects, SE—standard error

A short-term (one month) disutility of surgical intervention was also used. Firstly, the base value of 50% reduction in utility was calculated, based on Orlando et al [40]. Next, this base value was adjusted using relative severity of surgery, based on the Aristotle complexity score (ACS) [41] (Table 7). The base value of 50% reduction in quality of life was corresponding to the aortic valve replacement with ACS of 8.5.

**Table 7:** Basic Aristotle complexity score and short-term (monthly) disutility’s.

Procedures	Complexity Basic Score	Disutility value
Aortic valve replacement	8.5	-50%
VSD repair, Patch	6	-35,29%
Coarctation repair, end-to-end and patch aortoplasty	6	-35,29%
AVSD repair, Complete (CAVSD)	9	-52,94%
TOF repair, Ventriculotomy, Transanular patch	8	-47,06%
Arterial switch operation (ASO)	10	-58.82%
Aortic Valvuloplasty	8	-47,06%

VSD—ventricular septal defect; AVSD—atrioventricular septal defect; ToF—tetralogy of Fallot;

### Cost inputs

The cost of index surgery, reoperations, follow-up management (hospital stay, outpatient visits, tests, examinations, and drugs) was included in the analysis.

The UK expert inputs for resource use by defined CHD class were based on Mistry et al [34] and are presented in Table 8.

**Table 8:** Resource use data by CHD class.

CHD class	Outpatient visit frequency	Outpatient resource use	Inpatient stay frequency	Inpatient duration of stay	Additional inpatient resource use
Mild CHD disability	One visit every 4 years	ECG	One day stay every 15 years	2 days	-
		Chest x-ray			
		Transthoracic echo			
		30 mins of cardiologists’ time			
Moderate CHD disability	One visit every year	ECG	One day stay every 3 years	2 days	Cardiac MRI every 3rd year
		Chest x-ray			Exercise test every 3rd year
		30 mins of cardiologists’ time			Catheter every 15 years
		Echo every 2 years			
Severe CHD disability	Bi-annual visit	ECG	One day stay every 2 years	1 day	Cardiac MRI every 3rd year
		Chest x-ray			Exercise test every 3rd year
		30 mins of cardiologists’ time			Catheter every 10 years
		Echo every 2 years			

CHD—congenital heart defects, ECG—electrocardiogram, MRI—Magnetic resonance imaging

The cost of index surgery and reoperations was based on weighted average of Health Care Resource Groups (HRGs) for pediatric cardiac surgery procedures (Table 6). Analysis was performed using “HRG4+ 2015/16 Reference Costs Grouper” [42] in order to distinguish between different procedures used for index and redo surgeries. Unit cost of follow-up management is also presented in Table 9.

**Table 9:** Unit costs for resource use and cost of index surgery and reoperations.

Cost unit	Cost	References
**CHD class**		
Class I (Mild)	£ 315 (£ 59)	Mangham et al. [43]
Class II (Mild)	£ 693 (£ 95)	
Class III (Moderate)	£ 660 (£ 121)	
Class IV (Severe)	£ 1.206 (£ 237)	
**Drug—children from 1 to 18 years**		
Digoxin	£ 0.70	British National Formulary 2016 [44]
Furosemide	£ 12.07	
Warfarin	£ 1.47	
Amiodarone	£ 1.33	
Bisoprolol	£ 1.68	
Verapamil	£ 1.11	
Ramipril	£ 3.74	
**Drug—Adults, 18+ years**		
Digoxin	£ 1.04	British National Formulary 2016[44]
Furusemide	£ 0.62	
Warfarin	£ 1.47	
Amiodarone	£ 2.43	
Bisoprolol	£ 1.68	
Verapamil	£ 0.62	
Ramipril	£ 3.74	
**Hospital admissions—outpatient visits**		
Pediatric cardiology follow-up attendance	£ 183	UK Department of Health’s Reference Costs for 2014–2015 [45]
Adult cardiology follow-up attendance	£ 141	
Consultant cardiology cost (20 minutes)	£ 60	
**Hospital admissions—inpatient admissions**		
Congenital disorders (regular admission)	£ 361	UK Department of Health’s Reference Costs for 2014–2015 [45]
**Tests and investigations**		
ECG	£ 38	UK Department of Health’s Reference Costs for 2014–2015 [45]
Chest X-ray	£ 16	
Exercise test	£ 88	
MRI scan	£ 235	
Echocardiogram (outpatient)	£ 235	
Echocardiogram (inpatient)	£ 2274	
Catheter ≤ 18 years	£ 199	
Catheter ≥ 18 years	£ 340	
**Surgery cost**		
Complex Procedures for Congenital Heart Disease	£ 13235	Weighted average EC11 (A-C)[45]
Very Major Procedures for Congenital Heart Disease	£ 10537	Weighted average EC12 (A-C)[45]
Major Procedures for Congenital Heart Disease	£ 10537	Weighted average EC13 (A-C)[45]
Intermediate Procedures for Congenital Heart Disease	£ 5968	Weighted average EC14 (A-C)[45]
Minor Procedures for Congenital Heart Disease	£ 4967	Weighted average EC15 (A-B)[45]
Complex Repair of Ascending Thoracic Aorta	£ 15380	Weighted average ED14 (A-B)[45]
Standard Repair of Ascending Thoracic Aorta	£ 13049	Weighted average ED15 (A-B)[45]
Complex, Single Heart Valve Replacement or Repair	£ 13666	Weighted average ED24 (A-C)[45]
Standard, Single Heart Valve Replacement or Repair	£ 11158	Weighted average ED25 (A-C)[45]
Complex, Other Operations on Heart or Pericardium	£ 5968	Weighted average ED30 (A-C)[45]

CHD—congenital heart defects, ECG—electrocardiogram, MRI—Magnetic resonance imaging

### Uncertainty analysis

Uncertainty about data inputs was evaluated in deterministic and probabilistic sensitivity analysis per every CHD disease included into the analysis. Deterministic sensitivity analysis included varying each model parameter individually while holding other variables fixed at base-case values. Results are presented as Tornado diagrams. Monte Carlo probabilistic sensitivity analysis with 10,000 iterations were performed, where a random value for each variable is drawn based on a pre-determined distribution. The beta distribution was used for effectiveness and utility parameters; gamma distribution was used for cost and disutility parameters, normal distribution was used for age, and log-normal distribution was used for risk ratios.

### Analysis

In line with NICE recommendations, all costs and outcomes beyond the first year were discounted at 3.5% annually [46, 47]. Cardiac patch was considered cost-effective if the incremental cost-effectiveness ratio (ICER, which is calculated by dividing difference in cost between two arms by difference in QALY) was below the lower-bound willingness-to-pay threshold of £20,000/QALY [47]. Cardiac patch was considered a dominant strategy when associated expected effectiveness were greater and costs were smaller than the expected effectiveness and costs (also referred to as strong dominance or simple dominance).

Statistics analysis was performed using Stata 14 (StataCorp. 2015. Stata Statistical Software: Release 14. College Station, TX). The model was developed in Microsoft Excel 2016 (Microsoft Corp., Redmond, Washington, DC, USA).

## Results

### Base case analysis

According to model predictions, CardioCel was associated with lower incidence of re-operation, higher QALY and life years (LY), and lower overall costs when compared to any of the comparator patches (Table 10). CardioCel was dominating all three comparative patches.

**Table 10:** Base-case cost-effectiveness analysis.

	Cost, £	Δ cost, £	LY	Δ LY	QALY	Δ QALY	RI	RR	ICERs
**CardioCel vs Xenogeneic patches**									
Xenogeneic patches	27532		33.976		24.891		0.191		-
CardioCel	27434	-98	33.981	0.005	24.895	0.003	0.179	0.938	Dominant
**CardioCel vs Autologous patches**									
Autologous patches	27502		33.978		24.892		0.187		
CardioCel	27434	-68	33.981	0.003	24.895	0.002	0.179	0.956	Dominant
**CardioCel vs Synthetic patches**									
Synthetic patches	27594		33.973		24.889		0.199		
CardioCel	27434	-160	33.981	0.007	24.895	0.005	0.179	0.902	Dominant

LY—life years. QALY—quality adjusted life years, RI—reoperation incidence. RR—relative risk in reoperation., ICER—incremental cost-effectiveness ratio.

As patches are mutually exclusive treatments options, and therefore the base-case results are additionally reported (Tables A—L in S4 File) by calculating the incremental costs, effects, and ICER relative to the next most effective patch.

A similar pattern was observed for the model estimated results disaggregated by indication. Model estimations confirms that CardioCel again dominated all three comparator patch groups with cost savings maximized for AS across all three-patch type, whilst the smallest savings were estimated for VSD repair (Tables A—L in S4 File). Across all three comparator patch groups, cost savings were as follows (in order of decreasing savings): AS, AVSD, TGA, CoA, ToF, and VSD.

The model projections in relation to re-operation relative risk reduction was greatest in the AVSD with CardioCel 15%, 11%, and 23% for xenogeneic, autologous, and synthetic patches respectively. By comparison, the model prediction in relation to re-operation relative risk reduction associated with the use of CardioCel in AS repair was comparatively small with 1.6%, 1,2%, and 2.6% reductions estimated for xenogeneic, autologous, and synthetic patches respectively.

### Sensitivity analyses

Deterministic sensitivity analysis showed that the model most sensitive parameters are short-term operative mortality, the cost of reoperations, and utility values for CHD disability. CardioCel was cost saving according to model estimates and dominated all comparators within all investigated diseases.

Probabilistic sensitivity analysis demonstrated that CardioCel produced clinical benefits (additional QALYs) and had a cost-saving effect irrespective to an indication or chosen comparator among all comparators. Detailed disease-specific results together with breakdowns by reoperation aetiology and cost of surgeries versus the cost of follow-up fractions are presented in the Tables A—L and Figs A—AJ in S4 File).

## Discussion

Our model based economic evaluation informed by preliminary clinical outcome results suggests that the ADAPT tissue engineered bovine tissue pericardium scaffold (CardioCel), according to the simulation results, consistently dominates any of the xenogeneic, autologous, and synthetic comparator patches both in terms of being cost-saving and associated with a QALY gain across a 40-year time horizon.

This was observed across all indications in our model, although the magnitude of the advantage varied with the underlying anomaly. Predicted savings and QALYs gained were highest when CardioCel was used in the repair of congenital AVSD, relative to all three of the comparator patch groups. In contrast, CardioCel for ventricular septal defect and aortic stenosis repair was associated with the smallest predicted cost savings. Whilst this pattern was consistent across all three patch types, savings and QALYs were both highest when CardioCel was compared against synthetic patches, followed by xenogeneic, and then autologous patches according to the model predictions.

As short and long-term health outcomes associated with congenital cardiovascular defects improve and the associated survival gain leads to a population shift towards more adult patients [48, 49], so too is the potential gain in cost savings associated with avoiding reoperation secondary to patch failure or breakdown. In real terms, the primary model estimated cost savings associated with CardioCel for congenital cardiovascular anomaly repair are modest, with maximum savings per patient of £ 372 GBP over 40 years (CardioCel vs. synthetic patches in AVSD). However, the estimated relative reduction in re-operation risk was as high as 23% (CardioCel vs. synthetic patches, indication = AVSD) suggesting clinically significant gains in sub-groups of CHD patients, particularly those diagnosed with AVSD.

## Limitations

There are several limitations to this model-based economic evaluation. The presented analysis was informed by a variety of randomized and non-randomised data sources including national, administrative datasets and expert opinions in addition to trial data. Several of the data used in the primary analysis are limited by moderate to high risk of bias, secondary to retrospective designs and small sample size. Furthermore, estimation of the change of re-operation incidence due to the calcifications and the identification of factors associated with reoperation assumed that the overall risk of reoperation is the simple sum of its individual factors, which does not account for any correlation or interaction between subsets of these factors. Whilst the causes of re-operation are many and complex, there are limited data available that quantify the relationships between these explanatory variables and subsequent re-operation risk.

Further on, simulation of cohorts with state-transition models, can potentially exclude some important correlates of health and cost outcomes in a CHD population. In general, the ideal method is to randomize patients to appropriate comparative arms and follow all outcomes over time. However, comparing several different patches with 40 years of follow-up is not practical and feasible due to patch technology advancement over time and associated study costs. Therefore, despite all limitations, the decision-analytic simulation approach is the best reasonable alternative and similar conclusions have been reached in several other types of outcomes research in this field [50–52].

The focus of the present evaluation was upon the calcification patch properties only and their risk of subsequent reoperations. However, calcifications may also be associated with arrhythmias [53] and heart failure [54], each associated with their own unique suite of costs. The published data supporting these associations, however, is currently limited. Excluding the additional costs saved from avoiding these other conditions may underestimate the true benefit of CardioCel. Beyond reducing calcification, CardioCel has also been associated with the absence of surface thickening, thrombus formation, structural leak, and residual leak [13]. However, those properties and their effects on health and cost outcomes were not taken into consideration in these analyses, biasing our results against CardioCel.

The histological studies used for the adjustment of the patch related reoperations due to the calcifications are based on small sample sizes. The decision to use histological studies instead clinical studies, despite small sample size, was made after a detailed systematic review of the literature. Namely, in order to distinguish between different patch types, we conduct a systematic literature search in MEDLINE via PubMed, Cochrane, and EMBASE for the patch specific calcifications rates in CHD patients. The following keywords were used: “prostheses and implants", "transplants”,” biocompatible materials”, “patch”, “scaffold”, “cord”, *graft”, “septal occluder”, “bovine”, “porcine”, “equine”, “synthetic”, “biological”, “tissue”, “tissue-engineered”, “pericardium”, “homograft”, “homologous”, “autologous”, “xenograft” “heterograft”, “allograft”, “autograft”, “isograft”, "glutaraldehyde crosslinking", "glutaraldehyde fixation”. In total, 2026 articles were identified after deduplication. All studies identified by the search can be divided into two groups depending on the method used for assessing patch calcifications: (i) studies reporting echocardiography or other types of radiology assessment of implanted patches; and (ii) studies reported a histological examination of explanted patches.

In the early phase of our research, the focus was on clinical studies, which reported echocardiography or other types of radiology assessment of implanted patches. In total, 49 studies were identified. Only few studies were eligible for full-text review. The primary reason for exclusion of studies was that they reported calcification rates for a prosthesis, conduits, and valved conduits and not for patch or scaffolds. However, detailed analyses of the studies showed that there was no possibility to differentiate between patch type and rates of calcification, mostly due to differences in reporting and an infeasibility to distinguish between clinically consequential and insignificant calcifications. There is a clear need for the standardization in assessment and categorization of calcification severity, and currently evidence synthesis of the patch specific calcification rates according to their severity is not feasible from clinical studies.

However, the second approach (using histological studies) allows for more accurate quantification of the causal link between calcification and reoperation due to the reason that analyses were done on the explanted patches. Therefore, although the sample size of histological studies is much smaller, the relation between calcification and reoperation more accurately characterized. In such trade-off situation, we decide to use results of histological studies in the model, as it provides more reliable information regarding the clinical significance of calcifications in relation to reoperations. Unfortunately, other solutions cannot be expected in near to mid future. An international effort will be needed with linkage of several registries as well as their harmonization and standardization of the measurements.

In addition, modelled clinical pathway in the case of the isolated congenital aortic stenosis is the open surgical valvotomy in which in addition patch is used for the valve reconstruction. Subsequent reoperations are assumed to be exclusively repairs (reconstructions) with the same patch types (not replacements with prothesis). Although we are aware of a small number of infants in the projected clinical pathway in our model, we are forced to model in this way due to the need to isolate the patch effect which is not modified by other non-patch surgical approaches, both at the level of index surgery, but for the reoperations as well. This is the trade-off decision we must make to have a fair comparison of patches. Effect of switching to another patch or other surgical approaches in case of the reoperations was not possible to statistically control due to the lack of sufficient primary data sources. Therefore, the infrequent rather than standard of care pathway is modelled. To stay in line with current clinical practise and to limit potential bias in final results, AS indication was modelled using adequately low prevalence inputs in the overall CHD cohort. In addition, conservative assumption in relation to patch-related re-operations (16%) in this indication were taken into account to future minimise the impact of AS sub-cohort to final results. However, by developing new patch technologies as CardioCel, it is highly possible that clinical practise will shift toward using AS repairs not only as a temporary solution toward surgical replacement but rather as a long-term solution in both pediatric and adult patients.

The current evidence level in relation to differences in short and middle term outcomes among different patch classes lack direct head to head comparison. Future comparative studies are needed for more robust quantification of the patch calcification impact on re-operations, arrhythmias, and heart failure. Additional research is needed to shed light on the underlying causal relations of other important patch properties, as surface thickening, thrombus formation, structural leak, and residual leak, with mid and long-term outcomes. However, having in mind the differences in outcomes among different CHD diseases, which dictated the need for disease specific comparison and consequently small target populations, the realistic and feasible approach should be retrospective comparison, ideally based on registry data.

## Conclusion

A Markov state-transition model was developed to evaluate the cost effectiveness of the surgical repair of congenital heart diseases with bovine tissue engineered scaffold CardioCel compared with patches of xenogeneic, autologous, and synthetic origin. Model results demonstrated that CardioCel reduces reoperations, prolongs life, improves quality of life and saves costs compared xenogeneic, autologous, and synthetic patches. However, future research is needed to better quantify crucial causal relations links between patch properties and core outcomes.

## Supporting information

S1 File Reoperation etiological fractions. (PDF)

S2 File Freedom from reoperations inputs. (PDF)

S3 File Survival inputs. (PDF)

S4 File Disease specific results. (PDF)

S5 File Variables distributions and parameters used for the uncertainty analysis. (PDF)
