# Supplementary material for: Congenital heart defect repair with ADAPT tissue engineered pericardium scaffold: An early-stage health economic model
Source: PLoS One. 2018 Sep 27;13(9):e0204643. doi: 10.1371/journal.pone.0204643 (PMC6160133; doi:10.1371/journal.pone.0204643)
Supplement: S1 File — (PDF) [file pone.0204643.s001.pdf]

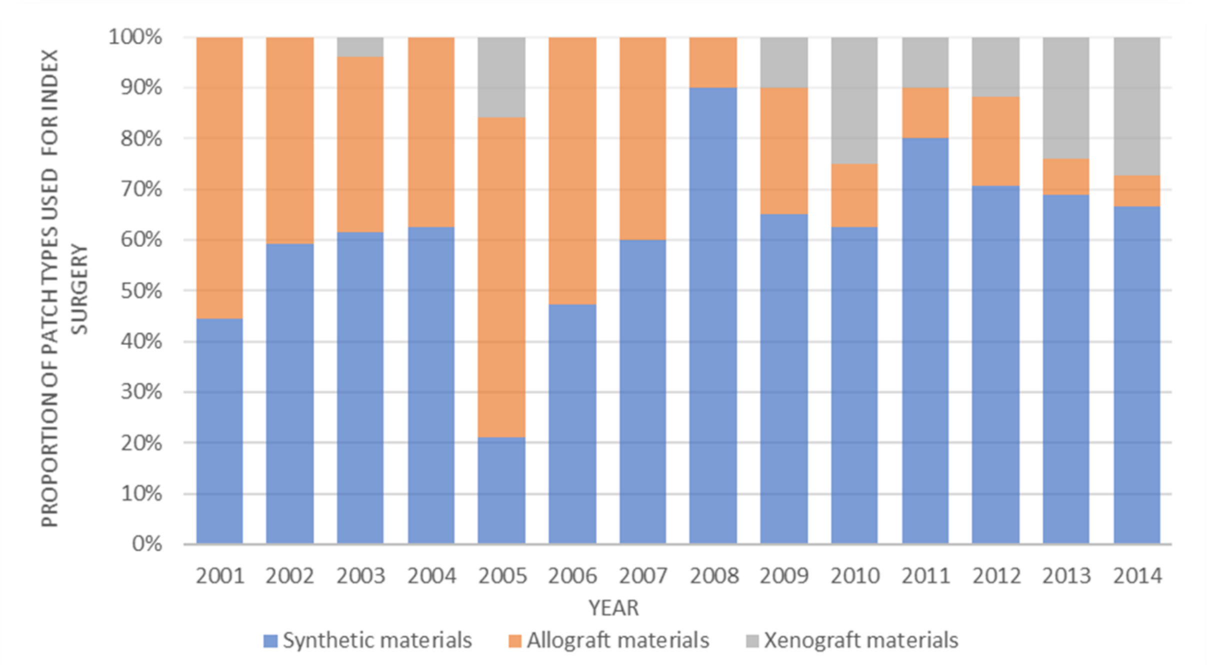

Fig A Historical trends of using different patches types for aortic valve valvotomy

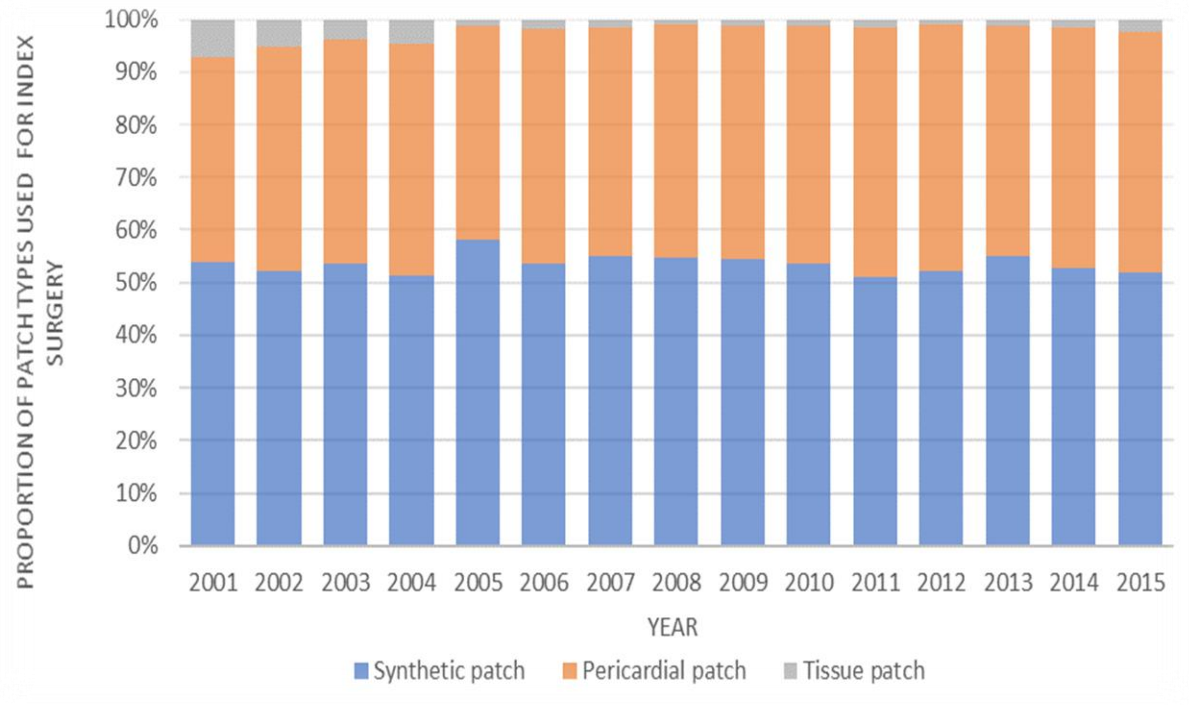

Fig B Historical trends of using different patches types for ventricular septum defect

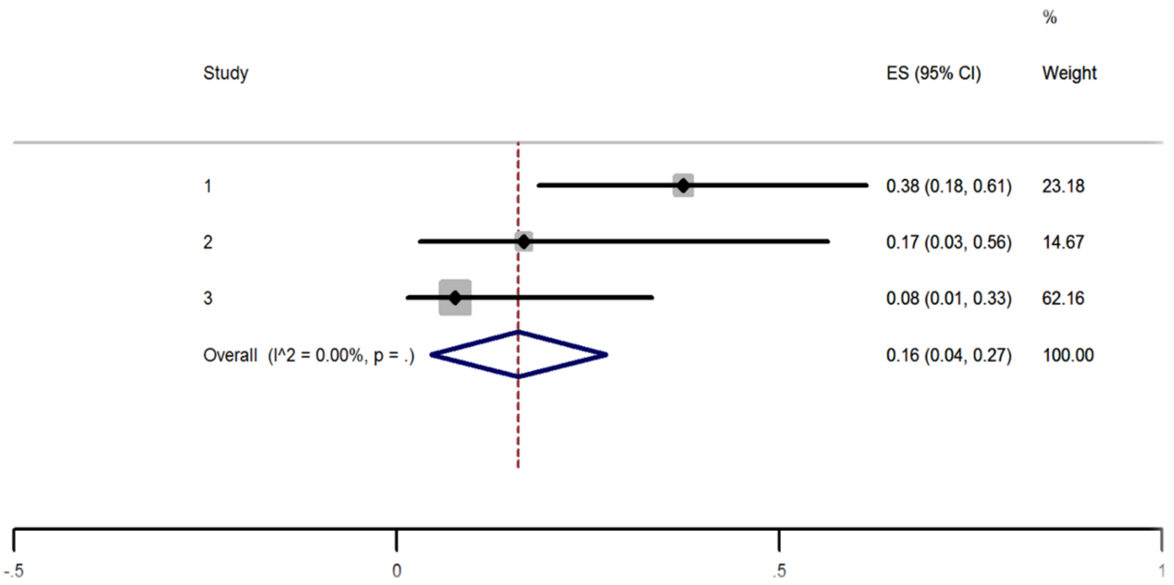

Fig C Ventricular septum defect: meta-analysis patch related reoperation fractions ([1], [2], [3])

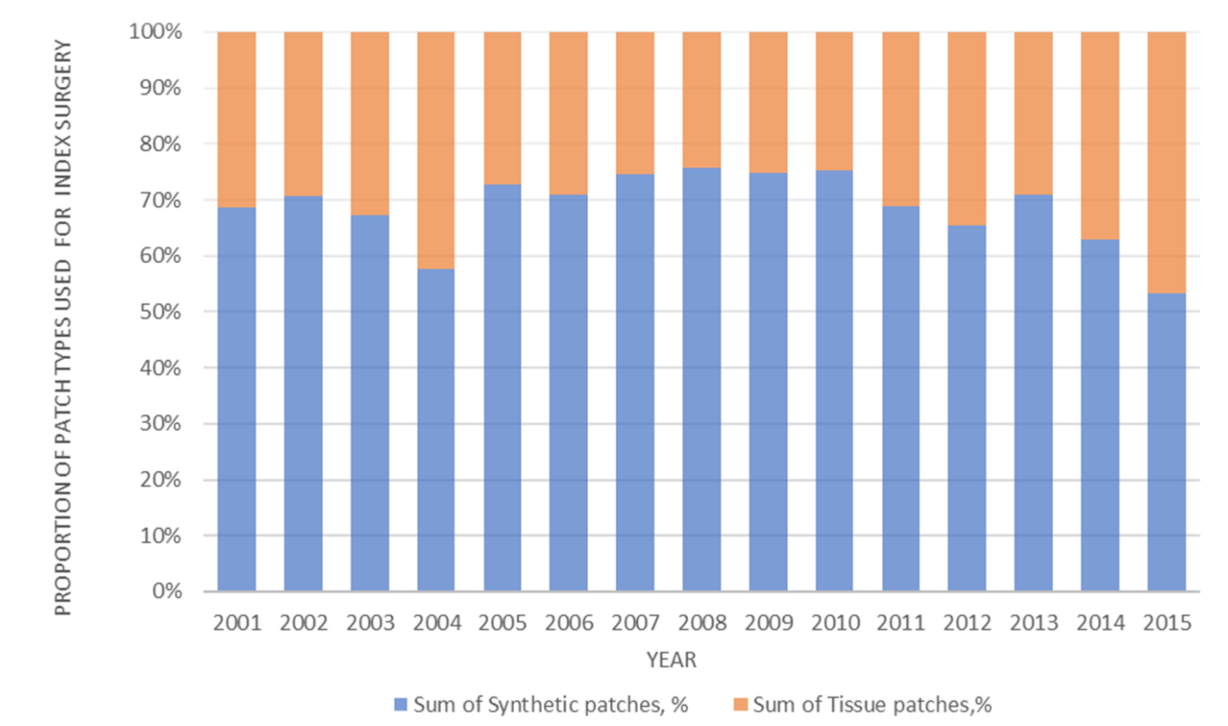

Fig D Historical trends of using different patches types for atrioventricular septum defect

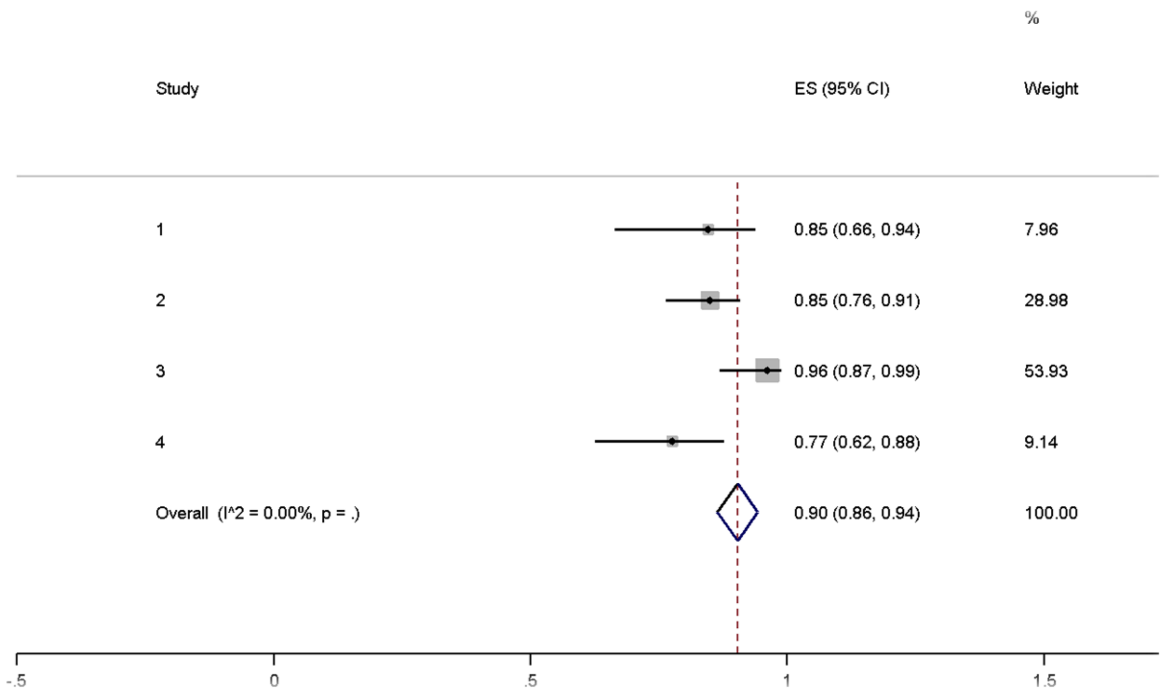

Fig E Atrioventricular septum defect: meta-analysis of patch related reoperation fractions ([4], [5], [6], [7])

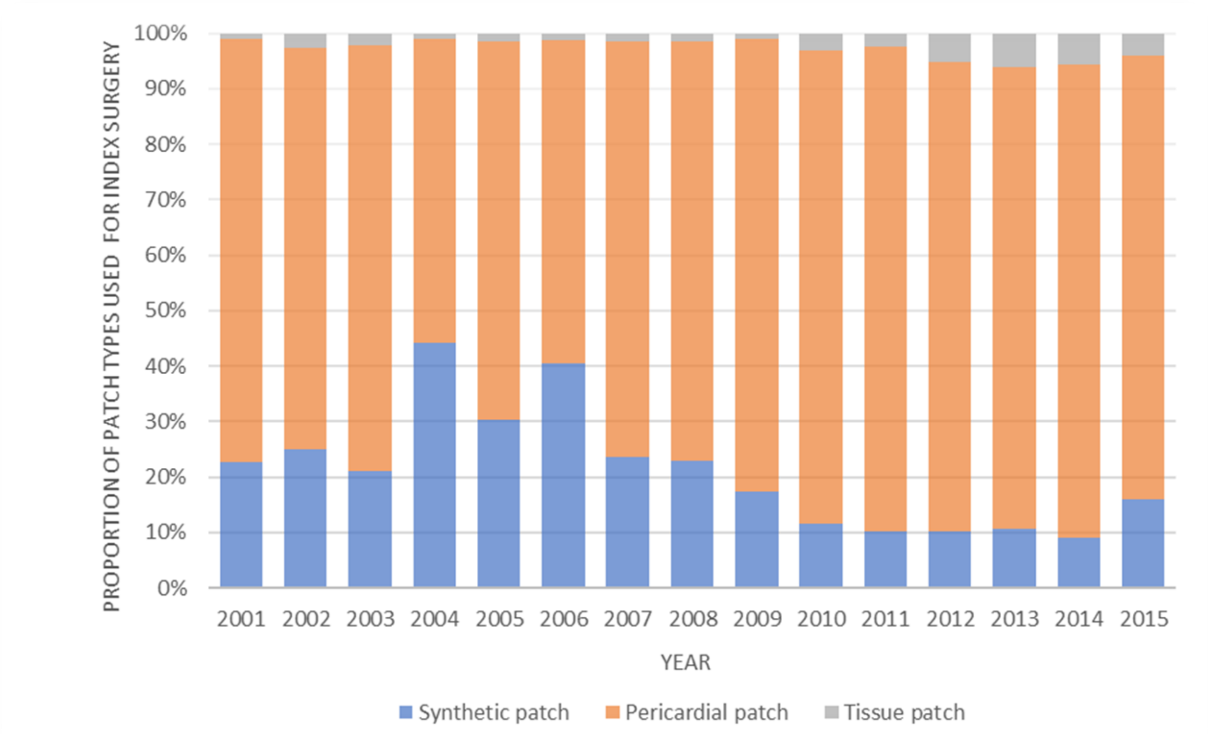

Fig F Historical trend of surgical procedures used for repair of transposition of great arteries

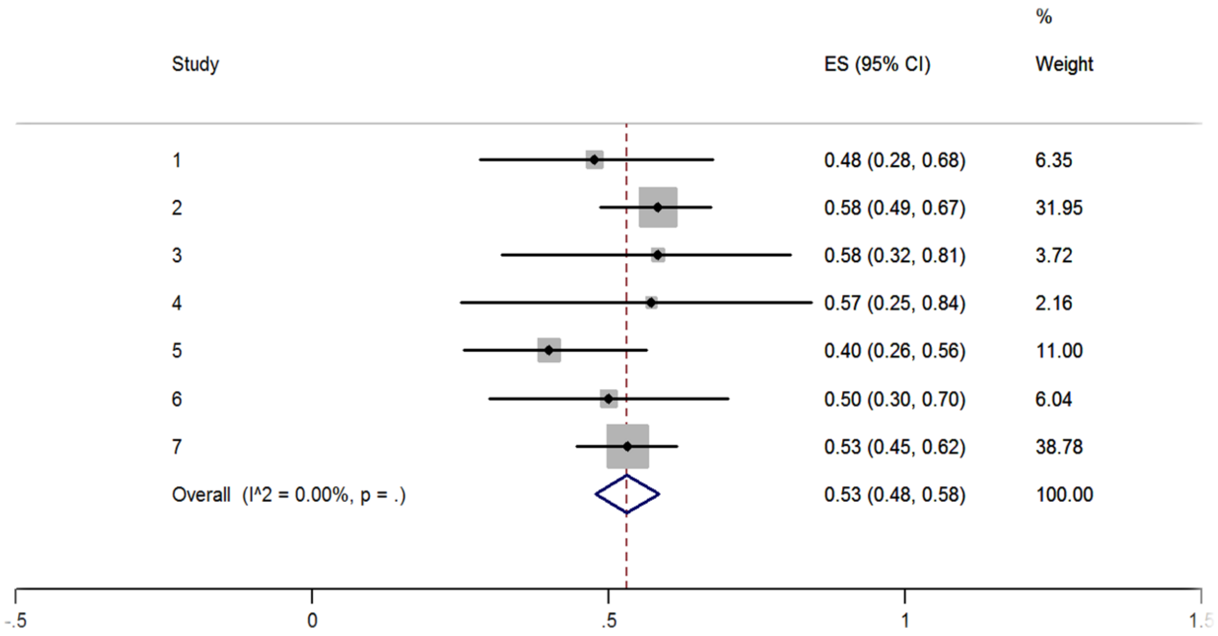

Fig G Transposition of great arteries: meta-analysis of patch related reoperation fractions ([8], [9], [10], [11], [12], [13])

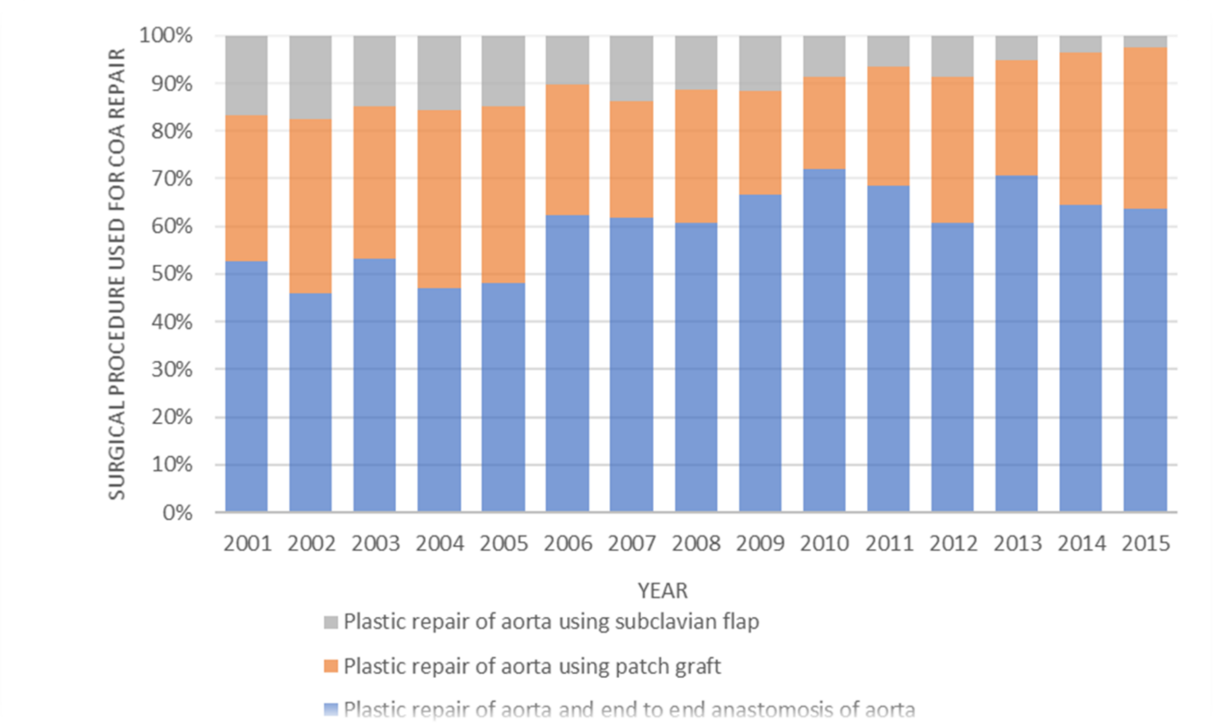

Fig H Historical trend of surgical procedures used for repair of coarctation of the aorta

Table A Patch specific calcification incidence pediatric CHD patients based on histological examination of explanted patches

Patch origin	Subtype/Brand name	Sample size	Calcification (%)	References
Xenogeneic patches	Porcine/CorMatrix	12	25%	Woo et al [14]
	Bovine pericardium/Peri-Guard®	3	0%	Majeed et al [15]
	Bovine pericardium/PhotoFix™	8	12.5%	
	Pooled estimate	23	18.2% (CI 2-35%)	Meta-analysis
Synthetic patches	ePTFE	4	50%	Hayabuchi et al [16]
	Fabric	4	0%	Tomizawa et al [17]
	ePTFE	10	30%	
	Pooled estimate	18	35% CI 10-60%)	Meta-analysis

Table B Congenital heart defects, appropriate surgical procedures and their relation to HRGs

Disease	ICD-10	OPCS	OPCS description	HRG
AVSD	Q212	K091	Repair of defect of atrioventricular septum using dual prosthetic patches	EC12
AVSD	Q212	K092	Repair of defect of atrioventricular septum using prosthetic patch NEC	EC12
AVSD	Q212	K093	Repair of defect of atrioventricular septum using tissue graft	EC12
AVSD	Q212	K096	Revision of repair of defect of atrioventricular septum	EC11
ToF	Q213	K043	Repair of tetralogy of Fallot using transannular patch	EC12
ToF	Q213	K044	Revision of repair of tetralogy of Fallot	EC11
TGA	Q259	K051	Repositioning of transposed great arteries	EC12
CoA	Q251	L233	Plastic repair of aorta using patch graft	ED15
CoA	Q251	L235	Revision of plastic repair of aorta	ED15
IAA	Q251	L237	Repair of interrupted aortic arch	ED14
VSD	Q210	K111	Repair of defect of interventricular septum using prosthetic patch	ED30
VSD	Q210	K112	Repair of defect of interventricular septum using pericardial patch	ED30
VSD	Q210	K113	Repair of defect of interventricular septum using tissue graft NEC	ED30
VSD	Q210	K115	Revision of repair of defect of interventricular septum	ED30
AS	Q230	K265	Aortic valve repair NEC	ED25
AS	Q230	K268	Other specified plastic repair of aortic valve	ED25
AS	Q230	K269	Unspecified plastic repair of aortic valve	ED25
AS	Q230	K302	Revision of plastic repair of aortic valve	ED24
HLS	Q234	K175	Biventricular repair of hypoplastic left heart syndrome	EC11

References

1. Bol-Raap G, Weerheim J, Kappetein AP, Witsenburg M, Bogers AJ. Follow-up after surgical closure of congenital ventricular septal defect. European journal of cardio-thoracic surgery : official journal of the European Association for Cardio-thoracic Surgery. 2003;24(4):511-5. PubMed PMID: 14500067.
2. Roos-Hesselink JW, Meijboom FJ, Spitaels SE, Van Domburg R, Van Rijen EH, Utens EM, et al. Outcome of patients after surgical closure of ventricular septal defect at young age: longitudinal follow-up of 22-34 years. European heart journal. 2004;25(12):1057-62. doi: 10.1016/j.ehj.2004.04.012. PubMed PMID: 15191777.
3. Mongeon FP, Burkhart HM, Ammash NM, Dearani JA, Li Z, Warnes CA, et al. Indications and outcomes of surgical closure of ventricular septal defect in adults. JACC Cardiovascular interventions. 2010;3(3):290-7. doi: 10.1016/j.jcin.2009.12.007. PubMed PMID: 20298987.
4. Ginde S, Lam J, Hill GD, Cohen S, Woods RK, Mitchell ME, et al. Long-term outcomes after surgical repair of complete atrioventricular septal defect. The Journal of thoracic and cardiovascular surgery. 2015;150(2):369-74. doi: 10.1016/j.jtcvs.2015.05.011. PubMed PMID: 26048271.
5. Sojak V, Kooij M, Yazdanbakhsh A, Koolbergen DR, Bruggemans EF, Hazekamp MG. A single-centre 37-year experience with reoperation after primary repair of atrioventricular septal defect. European journal of cardio-thoracic surgery : official journal of the European Association for Cardio-thoracic Surgery. 2016;49(2):538-44; discussion 44-5. doi: 10.1093/ejcts/ezv115. PubMed PMID: 25855593.
6. Hoohenkerk GJ, Bruggemans EF, Rijlaarsdam M, Schoof PH, Koolbergen DR, Hazekamp MG. More than 30 years' experience with surgical correction of atrioventricular septal defects. The Annals of thoracic surgery. 2010;90(5):1554-61. doi: 10.1016/j.athoracsur.2010.06.008. PubMed PMID: 20971263.
7. Buratto E, Ye XT, Bullock A, Kelly A, d'Udekem Y, Brizard CP, et al. Long-term outcomes of reoperations following repair of partial atrioventricular septal defect. European journal of cardio-thoracic surgery : official journal of the European Association for Cardio-thoracic Surgery. 2016. doi: 10.1093/ejcts/ezw018. PubMed PMID: 26920940.
8. Losay J, Touchot A, Serraf A, Litvinova A, Lambert V, Piot JD, et al. Late outcome after arterial switch operation for transposition of the great arteries. Circulation. 2001;104(12 Suppl 1):I121-6. PubMed PMID: 11568042.
9. Tobler D, Williams WG, Jegatheeswaran A, Van Arsdell GS, McCrindle BW, Greutmann M, et al. Cardiac outcomes in young adult survivors of the arterial switch operation for transposition of the great arteries. Journal of the American College of Cardiology. 2010;56(1):58-64. doi: 10.1016/j.jacc.2010.03.031. PubMed PMID: 20620718.
10. Angeli E, Raisky O, Bonnet D, Sidi D, Vouhe PR. Late reoperations after neonatal arterial switch operation for transposition of the great arteries. European journal of cardio-thoracic surgery : official journal of the European Association for Cardio-thoracic Surgery. 2008;34(1):32-6. doi: 10.1016/j.ejcts.2008.04.007. PubMed PMID: 18468448.
11. Prifti E, Crucean A, Bonacchi M, Bernabei M, Murzi B, Luisi SV, et al. Early and long term outcome of the arterial switch operation for transposition of the great arteries: predictors and functional evaluation. European journal of cardio-thoracic surgery : official journal of the European Association for Cardio-thoracic Surgery. 2002;22(6):864-73. PubMed PMID: 12467806.
12. Shim MS, Jun TG, Yang JH, Park PW, Kang IS, Huh J, et al. Current expectations of the arterial switch operation in a small volume center: a 20-year, single-center experience. Journal of cardiothoracic surgery. 2016;11:34. doi: 10.1186/s13019-016-0428-9. PubMed PMID: 26911711; PubMed Central PMCID: PMCPMC4766671.
13. Serraf A, Roux D, Lacour-Gayet F, Touchot A, Bruniaux J, Sousa-Uva M, et al. Reoperation after the arterial switch operation for transposition of the great arteries. The Journal of thoracic and cardiovascular surgery. 1995;110(4 Pt 1):892-9. PubMed PMID: 7475154.
14. Woo JS, Fishbein MC, Reemtsen B. Histologic examination of decellularized porcine intestinal submucosa extracellular matrix (CorMatrix) in pediatric congenital heart surgery. Cardiovascular pathology : the official journal of the Society for Cardiovascular Pathology. 2016;25(1):12-7. doi: 10.1016/j.carpath.2015.08.007. PubMed PMID: 26453090.
15. Majeed A, Baird CW, Borisuk MJ, Sanders S, Padera R. Histology of Pericardial Tissue Substitutes Used in Congenital Heart Surgery. Pediatr Dev Pathol. 2015. doi: 10.2350/15-08-1696-OA.1. PubMed PMID: 26492092.
16. Hayabuchi Y, Mori K, Kitagawa T, Sakata M, Kagami S. Polytetrafluoroethylene graft calcification in patients with surgically repaired congenital heart disease: evaluation using multidetector-

row computed tomography. American heart journal. 2007;153(5):806 e1-8. doi: 10.1016/j.ahj.2007.01.035. PubMed PMID: 17452157.

17. Tomizawa Y, Takanashi Y, Noishiki Y, Nishida H, Endo M, Koyanagi H. Evaluation of small caliber vascular prostheses implanted in small children: activated angiogenesis and accelerated calcification. ASAIO journal (American Society for Artificial Internal Organs : 1992). 1998;44(5):M496-500. PubMed PMID: 9804480.
