# Supplementary material for: Congenital heart defect repair with ADAPT tissue engineered pericardium scaffold: An early-stage health economic model
Source: PLoS One. 2018 Sep 27;13(9):e0204643. doi: 10.1371/journal.pone.0204643 (PMC6160133; doi:10.1371/journal.pone.0204643)
Supplement: S2 File — (PDF) [file pone.0204643.s002.pdf]

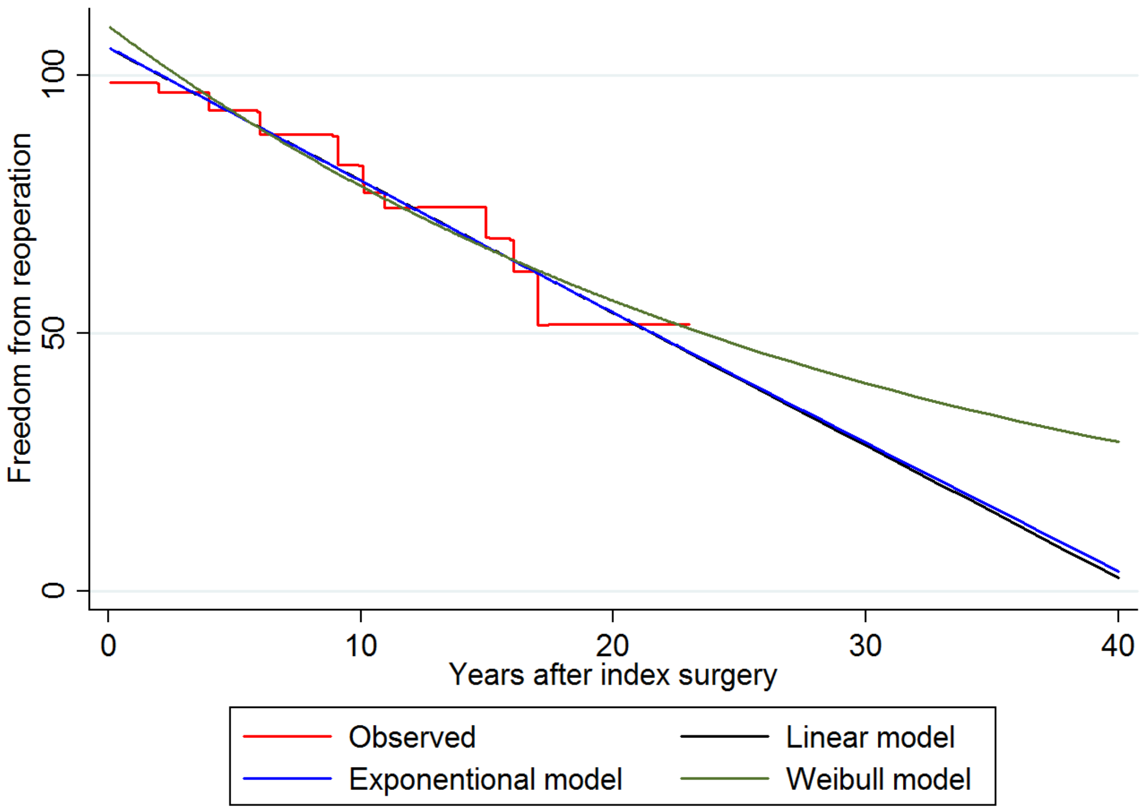

Fig A Observed and extrapolated freedom from reoperations after aortic valve valvotomy

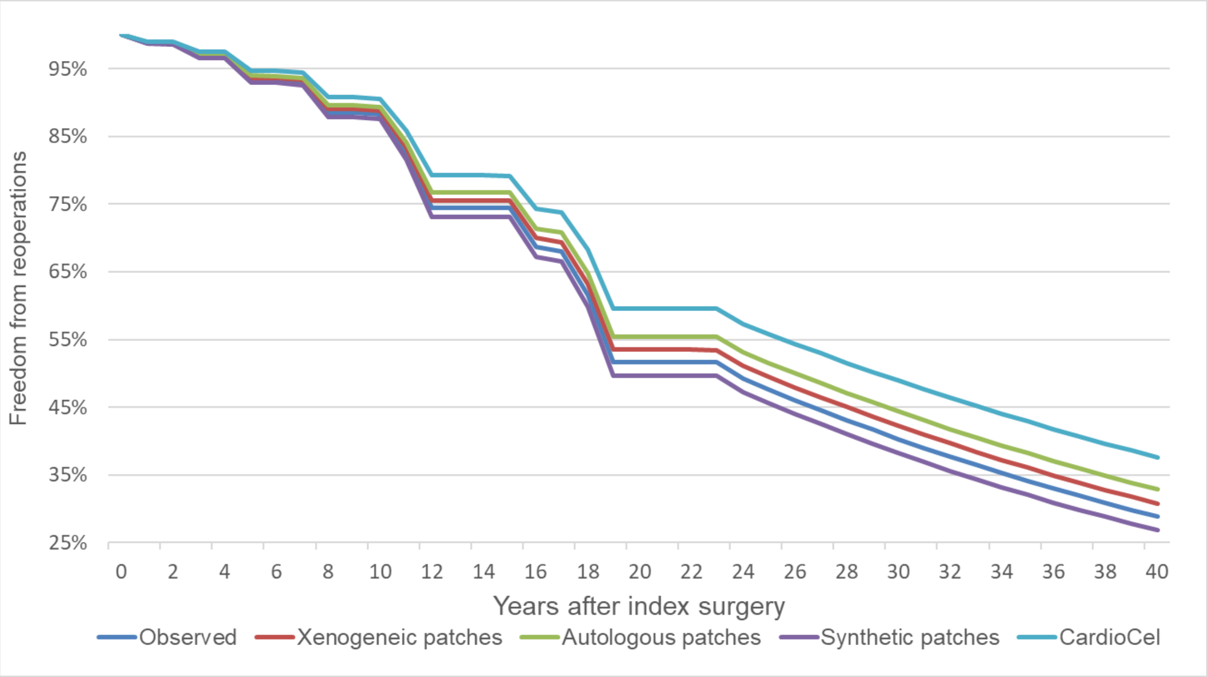

Fig B Observed and estimated freedom from reoperation by patch type after valvotomy

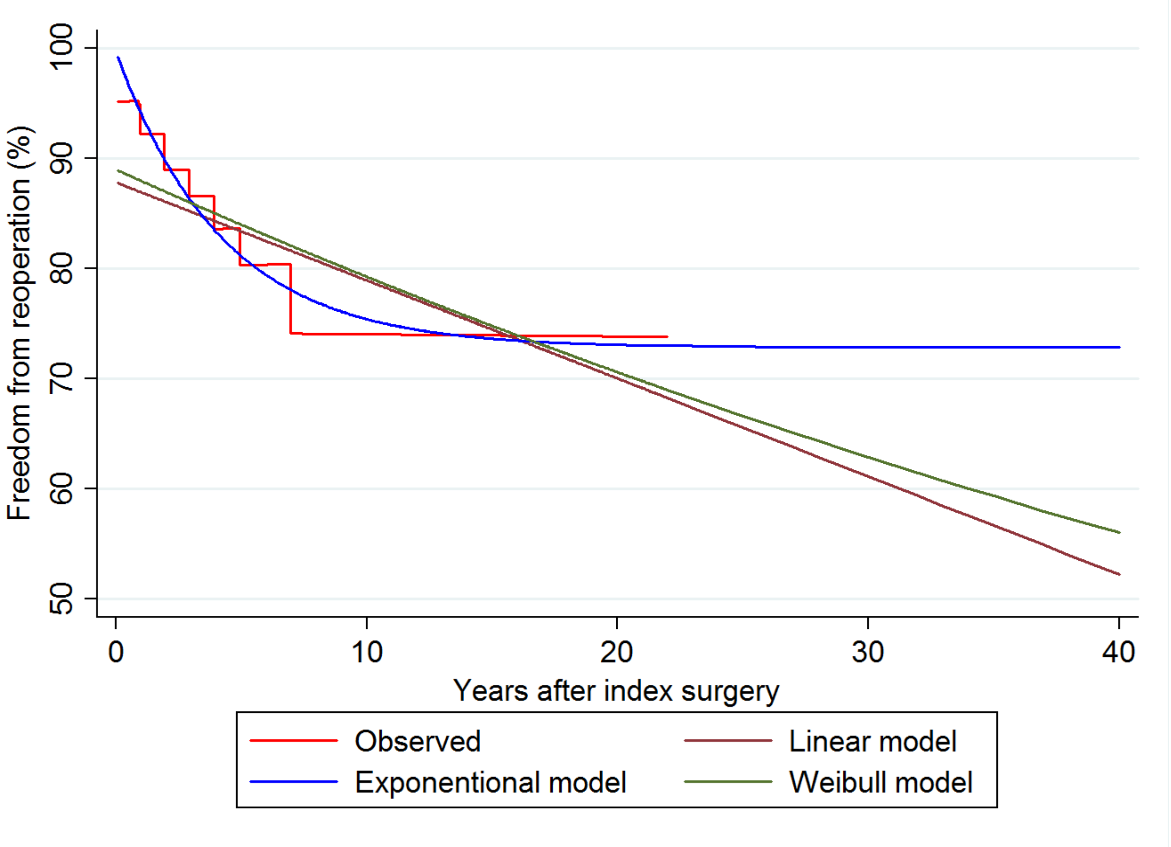

Fig C Observed and extrapolated freedom from reoperation after complete repair of AVSD

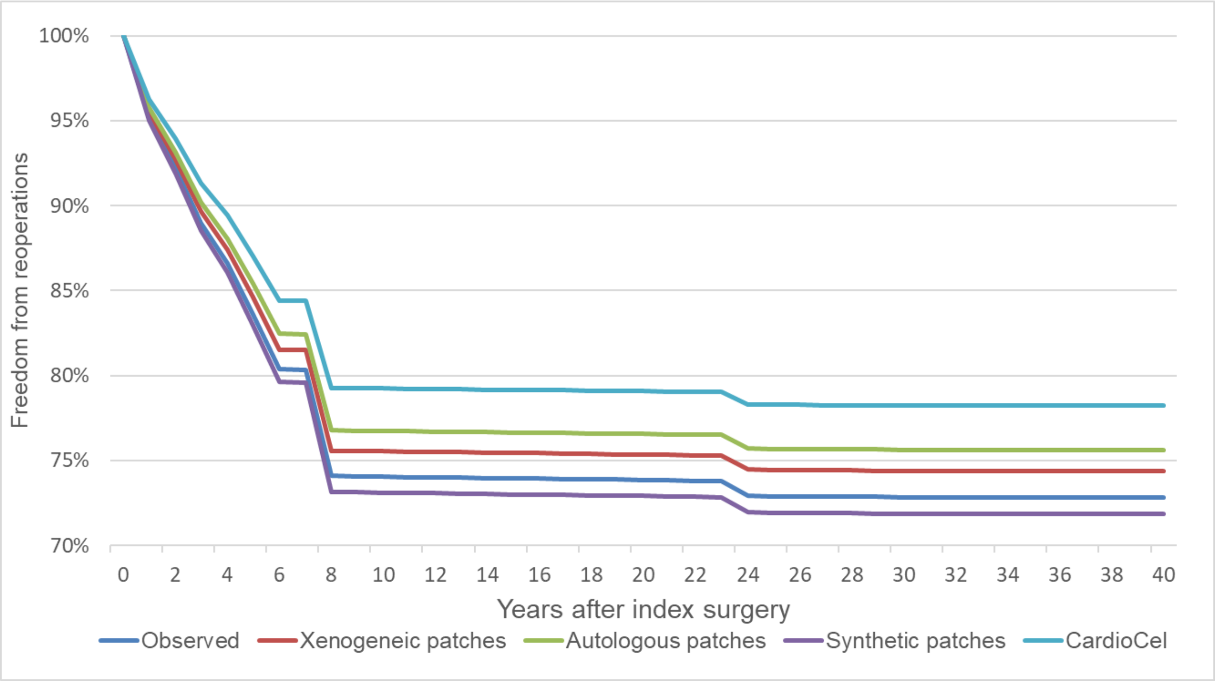

Fig D Observed and estimated freedom from reoperation by patch type after complete AVSD repair

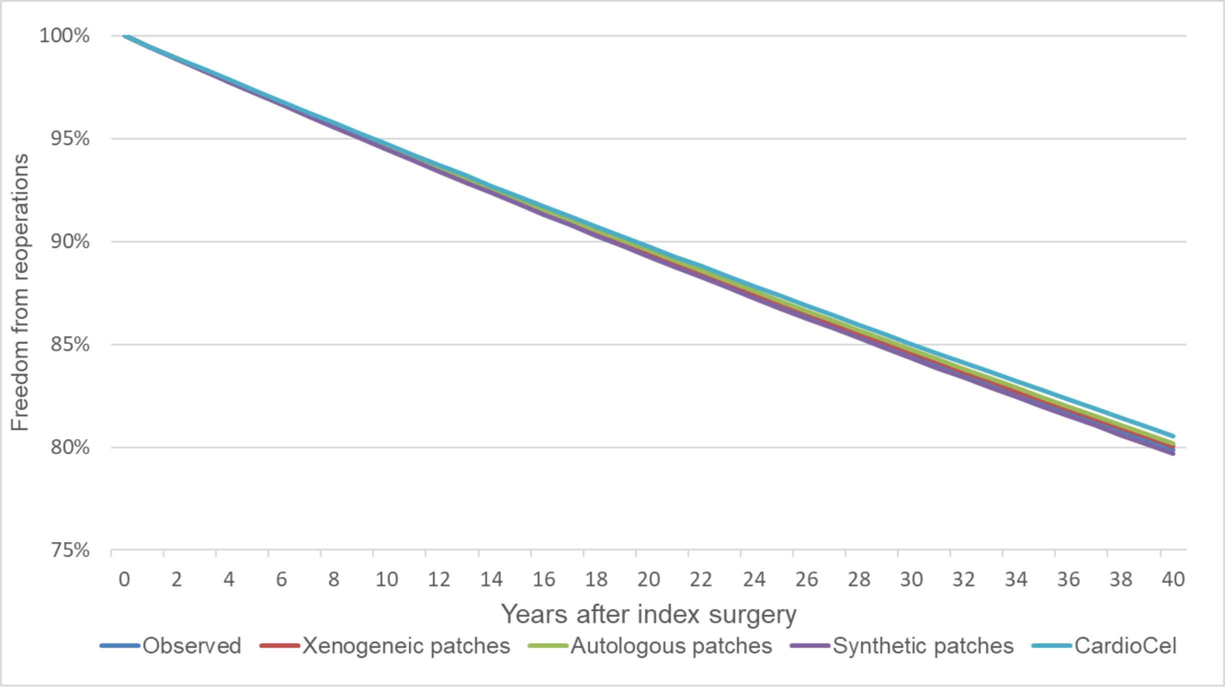

Fig E Observed and estimated freedom from reoperation by patch type after VSD repair

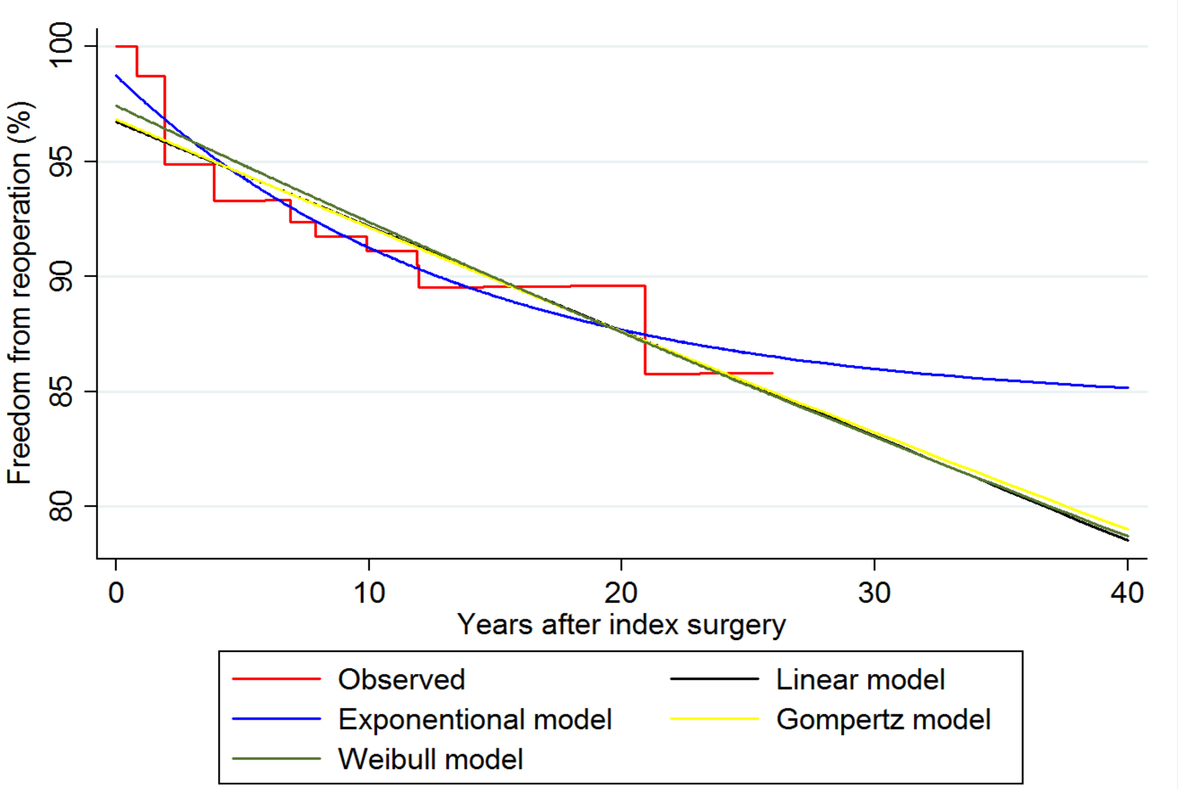

Fig F Observed and extrapolated freedom from reoperation after ToF repair

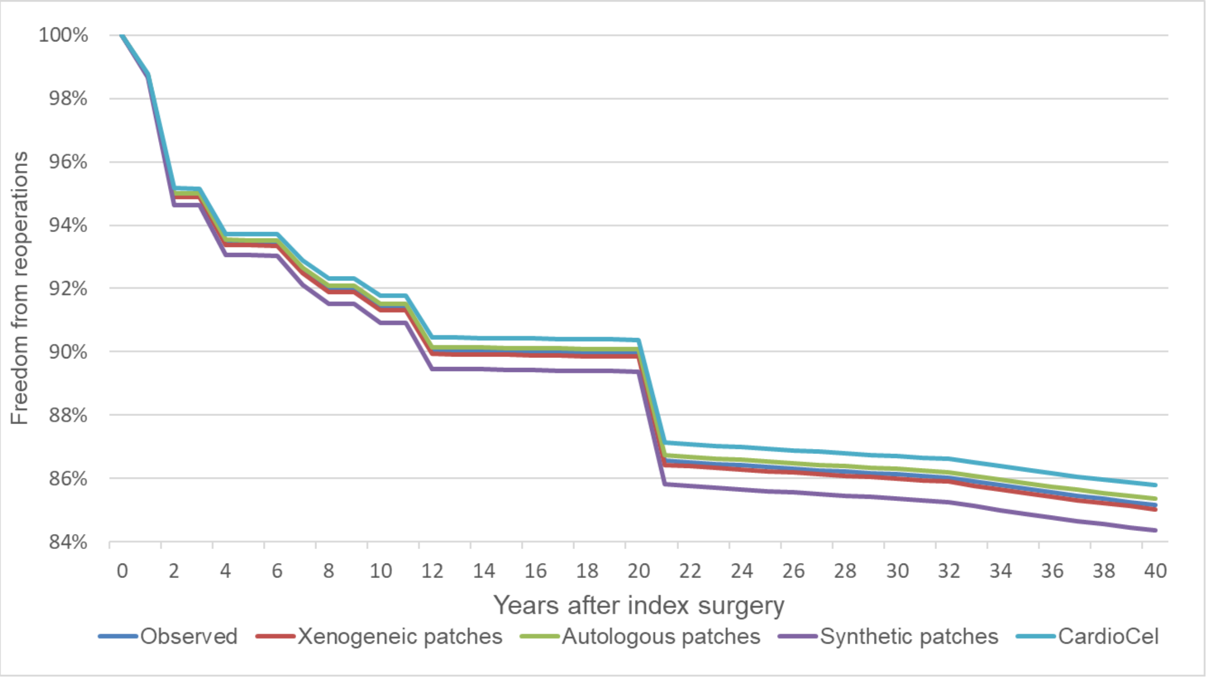

Fig G Observed and estimated freedom from reoperation by patch type after ToF repair

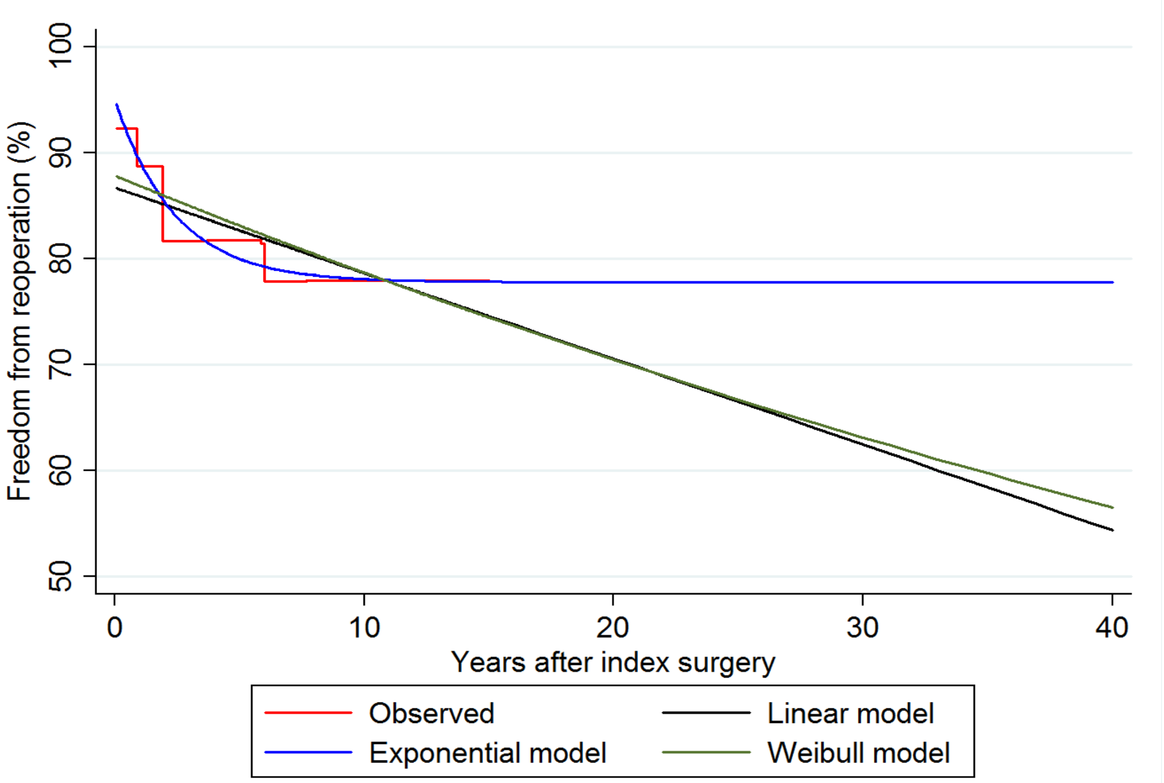

Fig H Observed and extrapolated freedom from reoperation after TGA repair

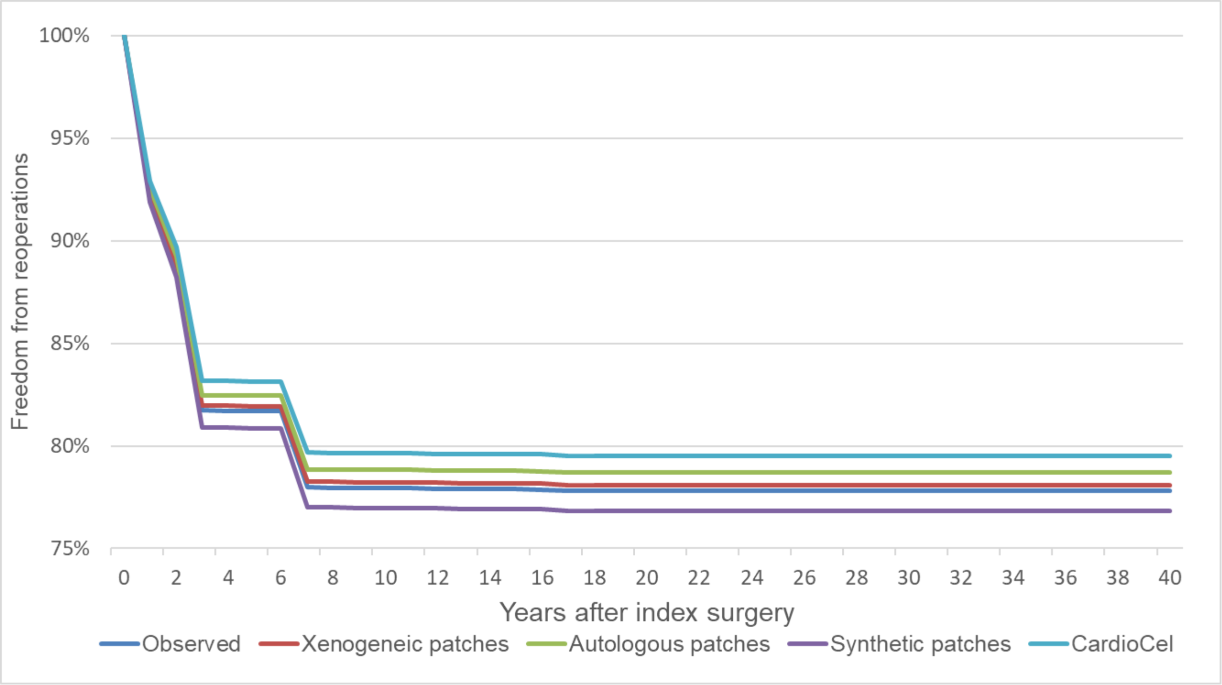

Fig I Observed and estimated freedom from reoperation by patch type after TGA repair

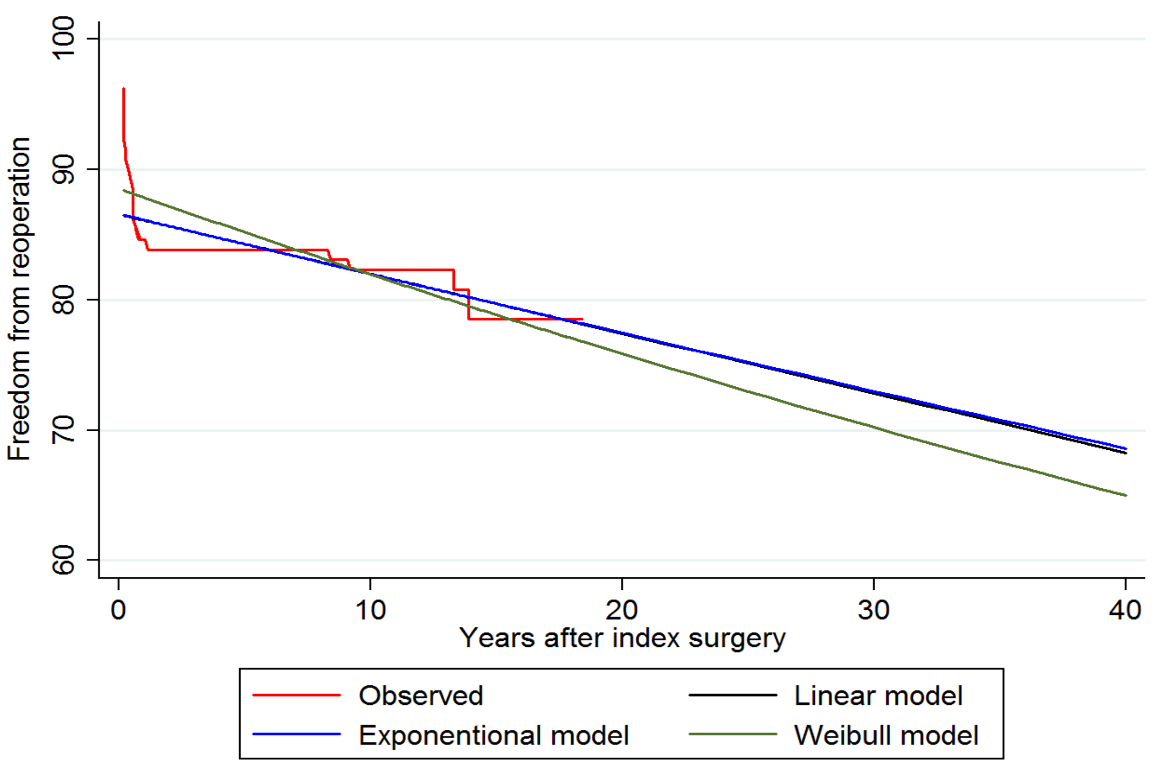

Fig J Observed and extrapolated freedom from reoperation after coarctation repair

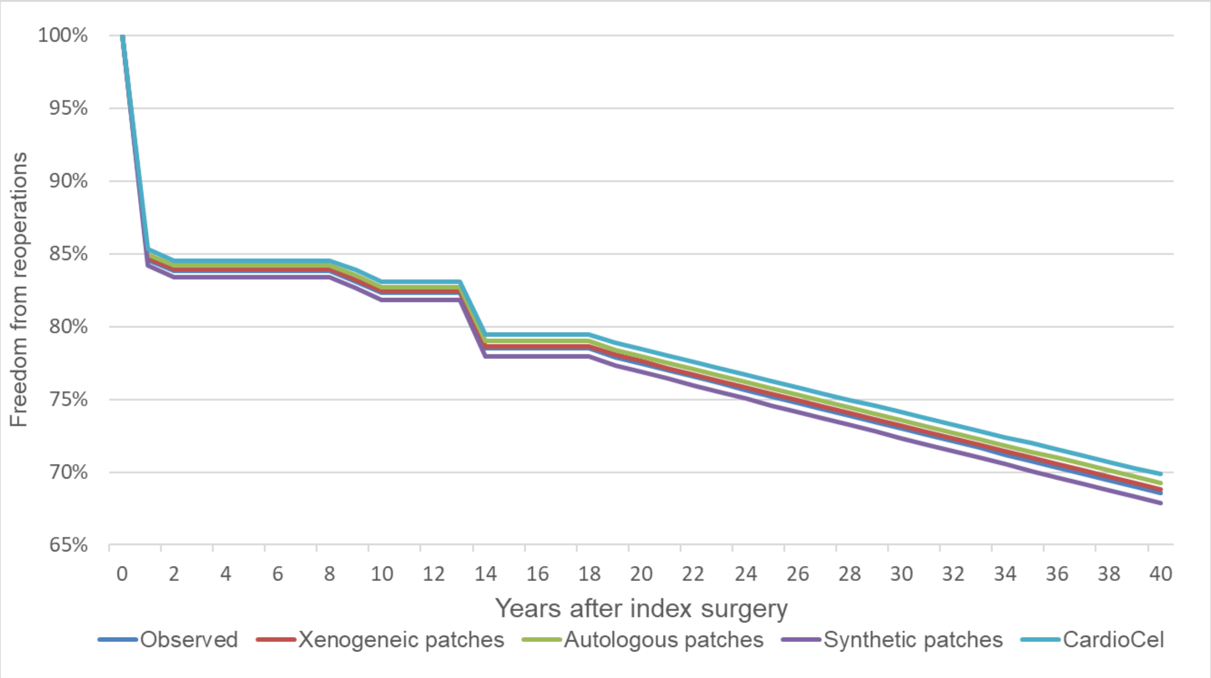

Fig K Observed and estimated freedom from reoperation by patch type arterial switch procedure
