# Supplementary material for: Congenital heart defect repair with ADAPT tissue engineered pericardium scaffold: An early-stage health economic model
Source: PLoS One. 2018 Sep 27;13(9):e0204643. doi: 10.1371/journal.pone.0204643 (PMC6160133; doi:10.1371/journal.pone.0204643)
Supplement: S3 File — (PDF) [file pone.0204643.s003.pdf]

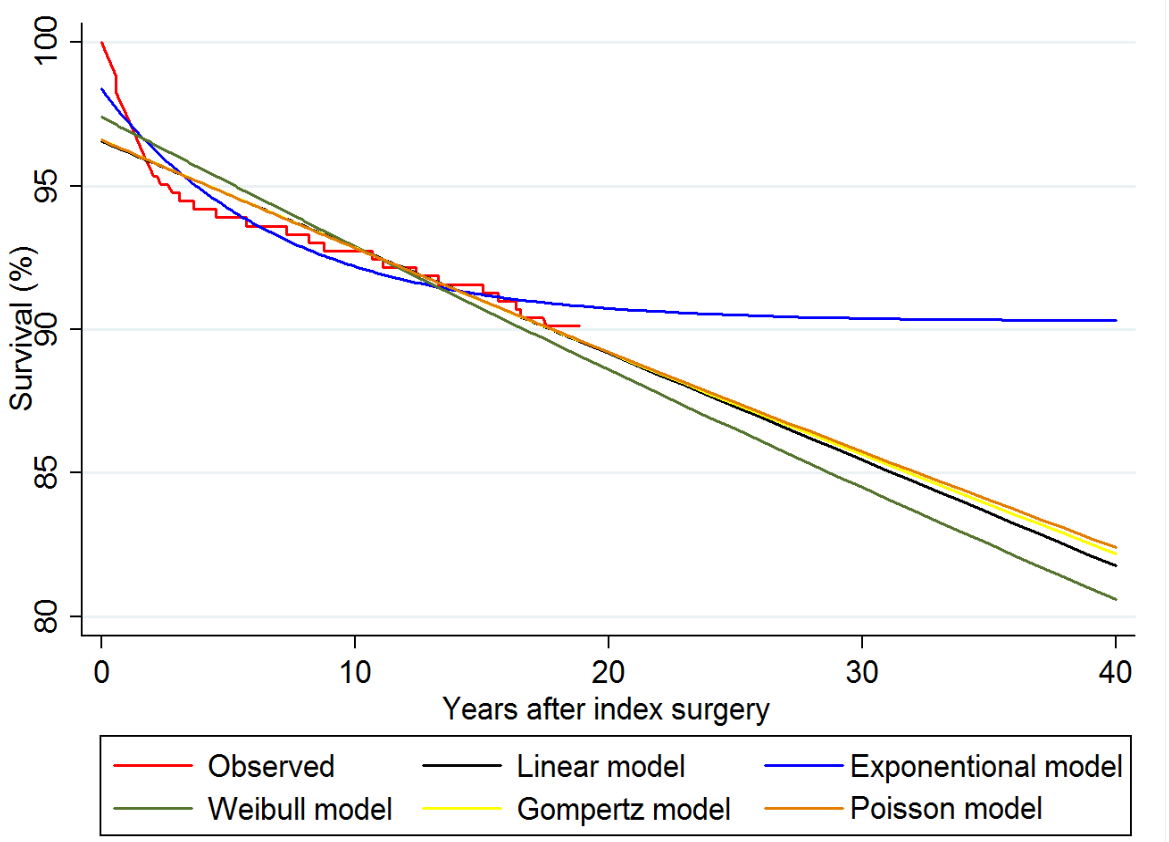

Fig A Observed and fitted survival distributions after index surgery for all CHD

Table A Model comparison – survival after CHD surgery

Model	AIC	BIC
Linear	-1003.897	-997.9164
Exponential	-1109.965	-1100.994
Weibull	-923.4359	-914.4646
Poisson	289.1342	295.1151

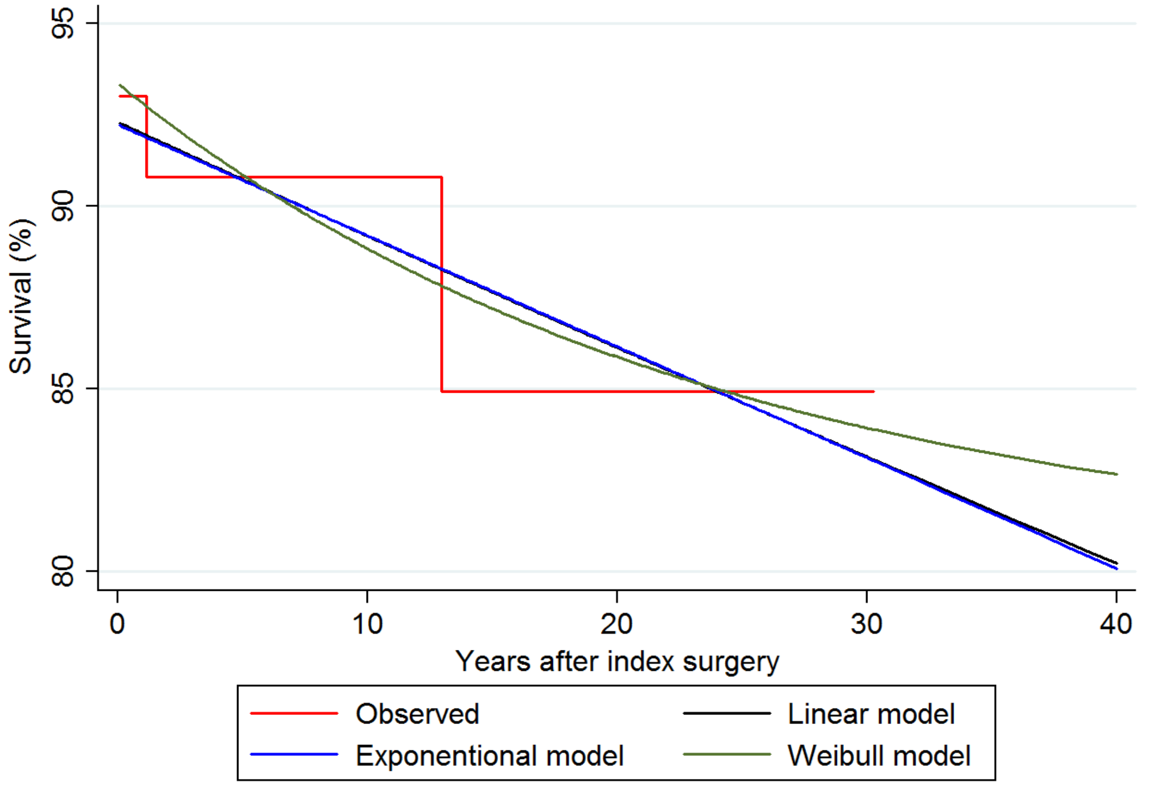

Fig B Observed and fitted survival distributions after index surgery for AS

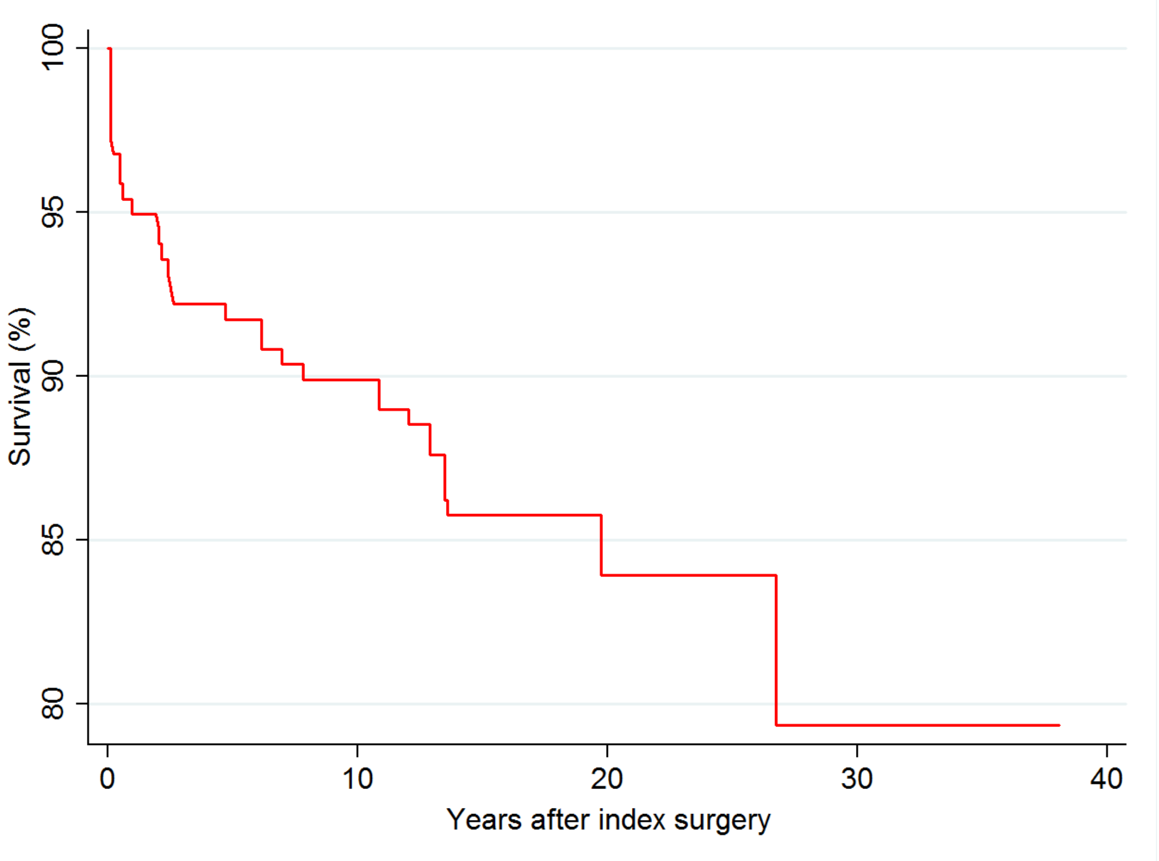

Fig C Observed survival distributions after index surgery for complete AVSD surgery

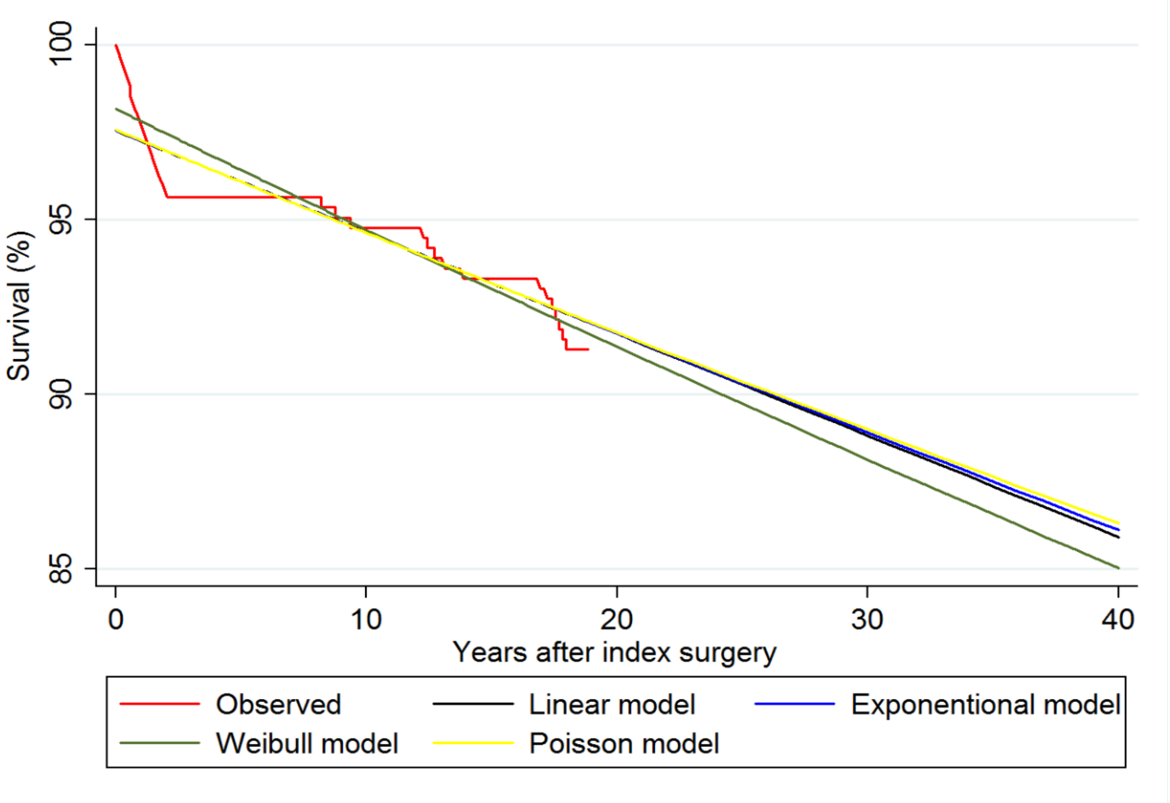

Fig D Observed and fitted survival distributions after index surgery for VSD

Table B Model comparison – survival after VSD

Model	AIC	BIC
Linear	-1814.439	-1806.841
**Exponential**	**-1853.399**	**-1842.001**
Weibull	-1742.534	-1731.136

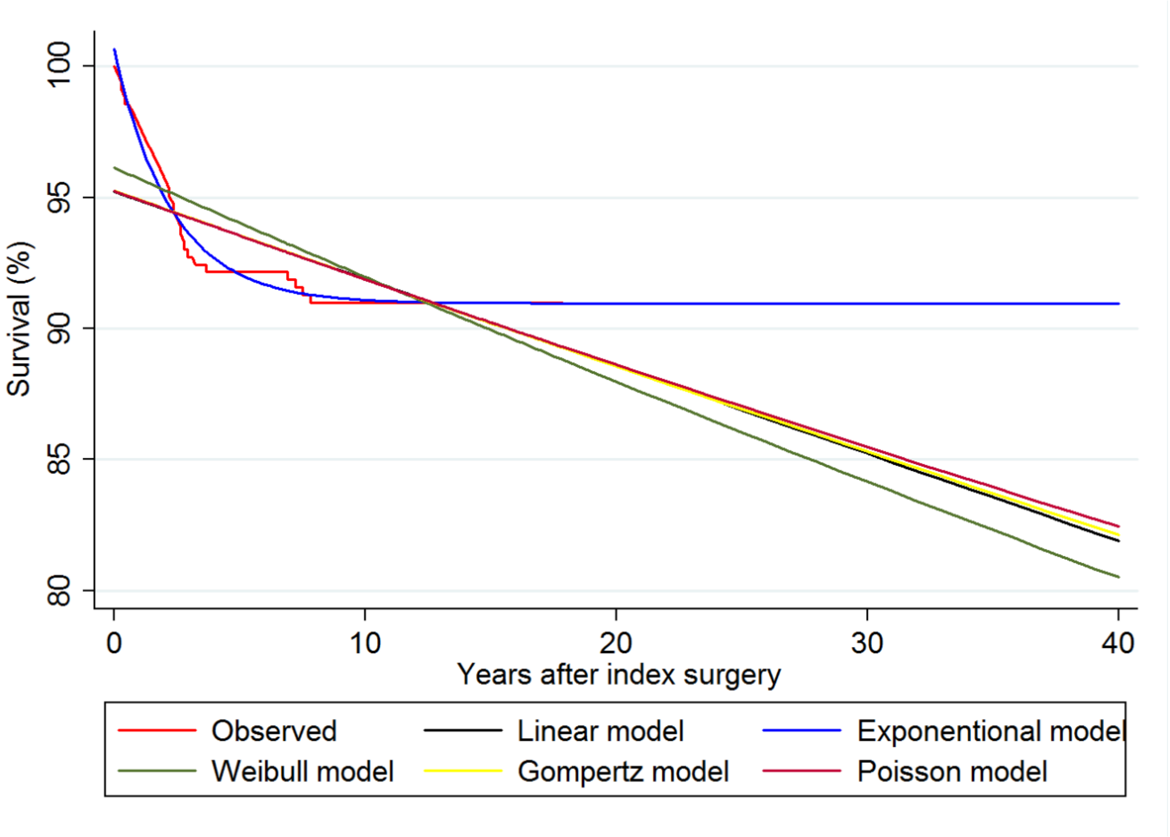

Fig E Observed and fitted survival distributions after index surgery for ToF

Table C Model comparison – survival after ToF

Model	AIC	BIC
Linear	-737.0459	-731.2502
**Exponential**	**-1125.587**	**-1116.894**
Weibull	-679.9804	-671.2869
Poisson	263.1039	268.8996

No single model has a good representation of the UK practise, therefore piecewise model is used.

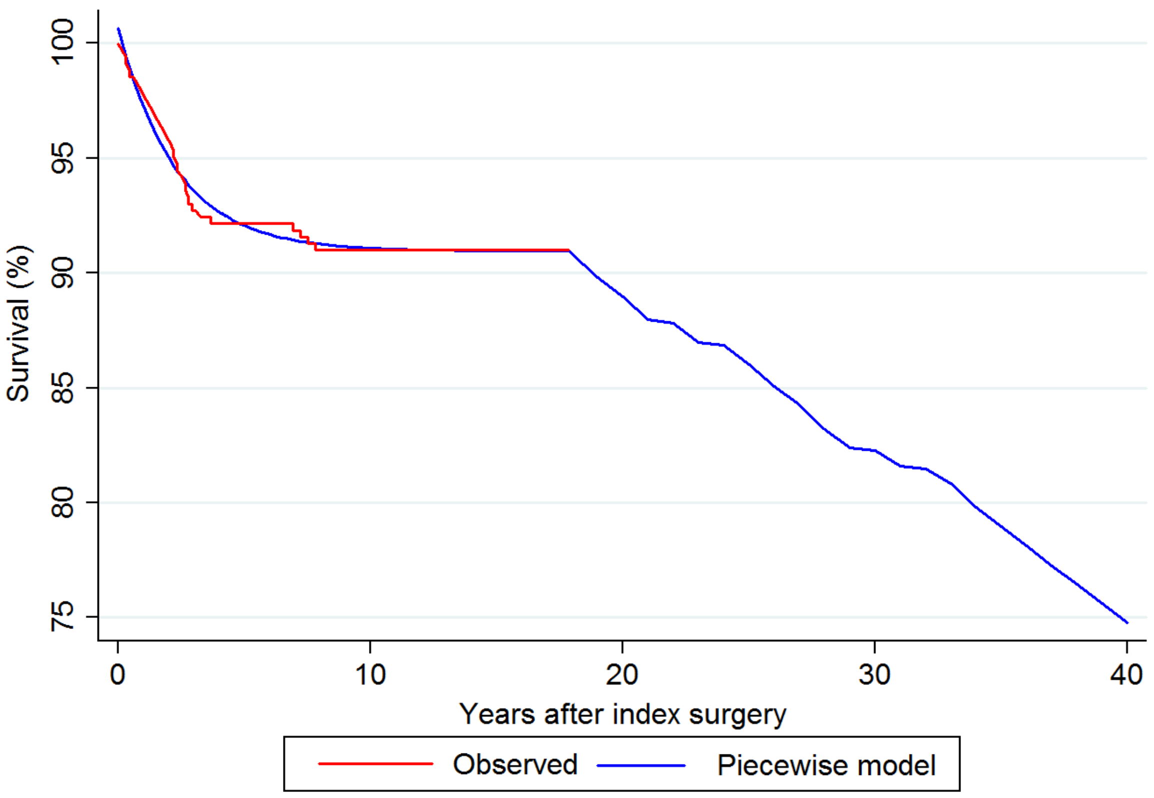

Fig F Observed and fitted survival distributions using piecewise model

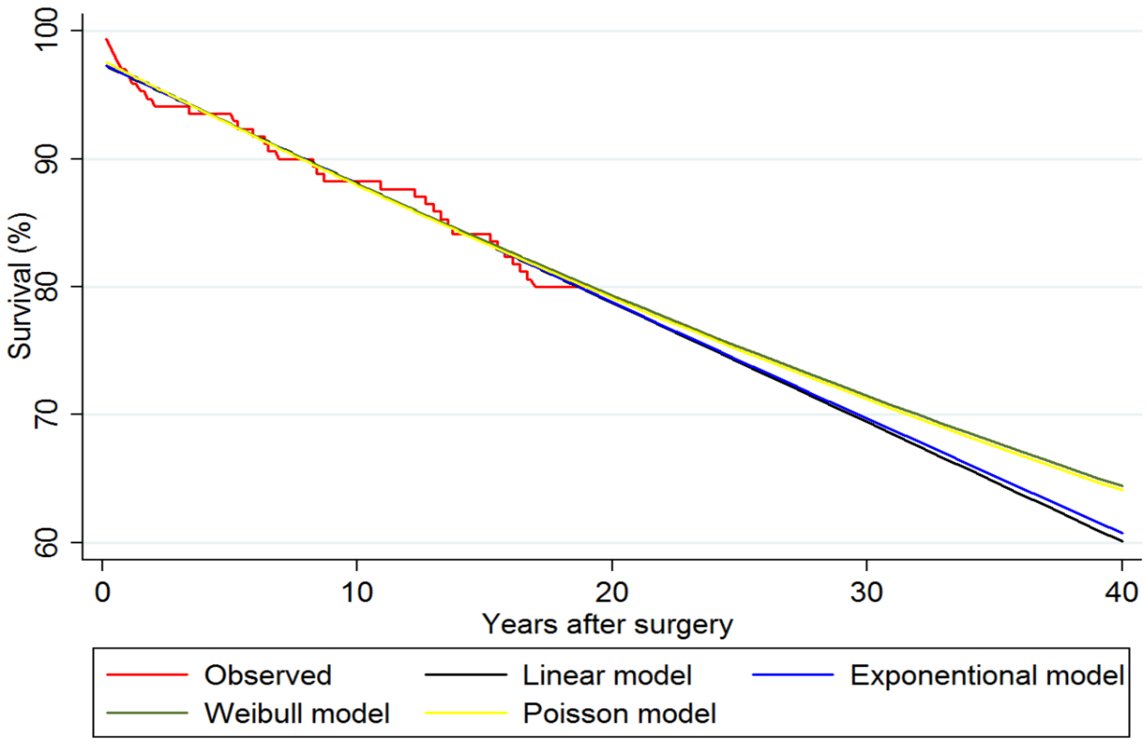

Fig G Observed and fitted survival distributions after index surgery for TGA

Table D Model comparison – survival after TGA

Model	AIC	BIC
Linear	-1038.554	-1032.546
Exponential	-1036.639	-1030.631
Weibull	-958.9102	-949.8984
Poisson	286.3111	292.319

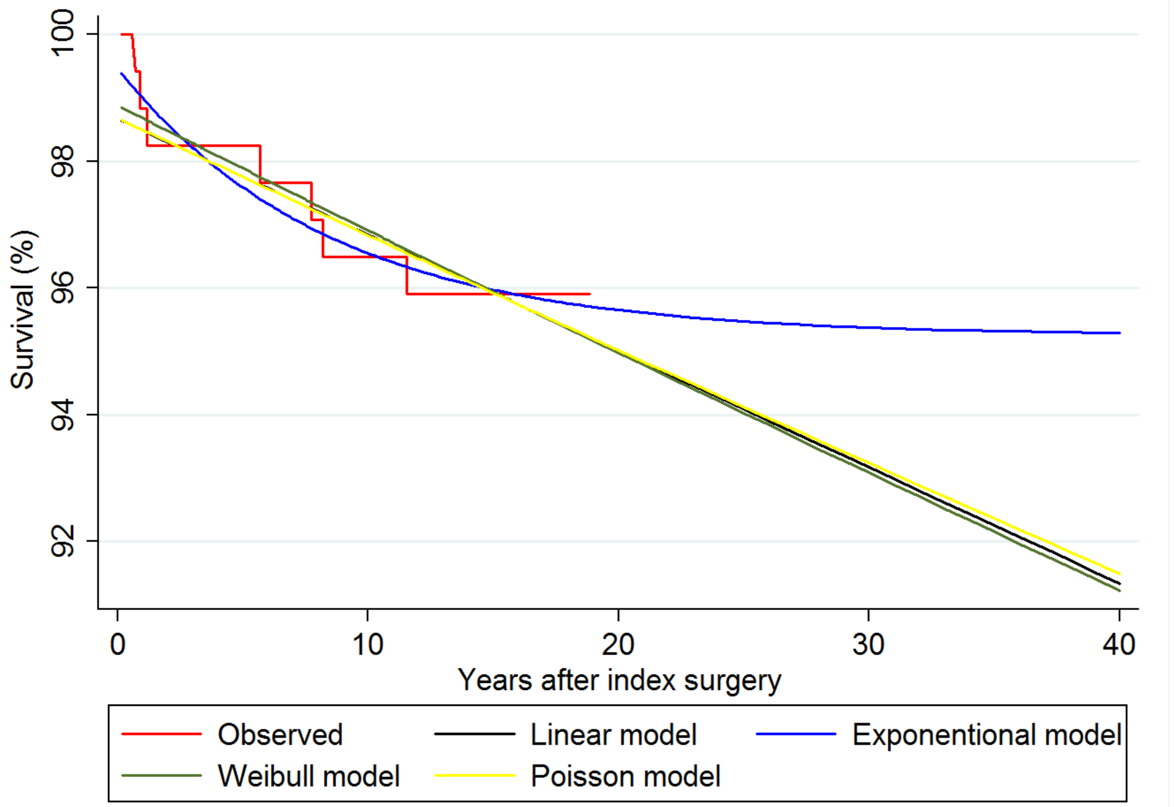

Fig H Observed and fitted survival distributions after index surgery for CoA

Table E Model comparison – survival after CoA

Model	AIC	BIC
Linear	-1002.11	-996.314
**Exponential**	**-1048.846**	**-1040.152**
Weibull	-978.9499	-970.2564
Poisson	268.5117	274.3074

Overview of average and disease specific survival in the UK

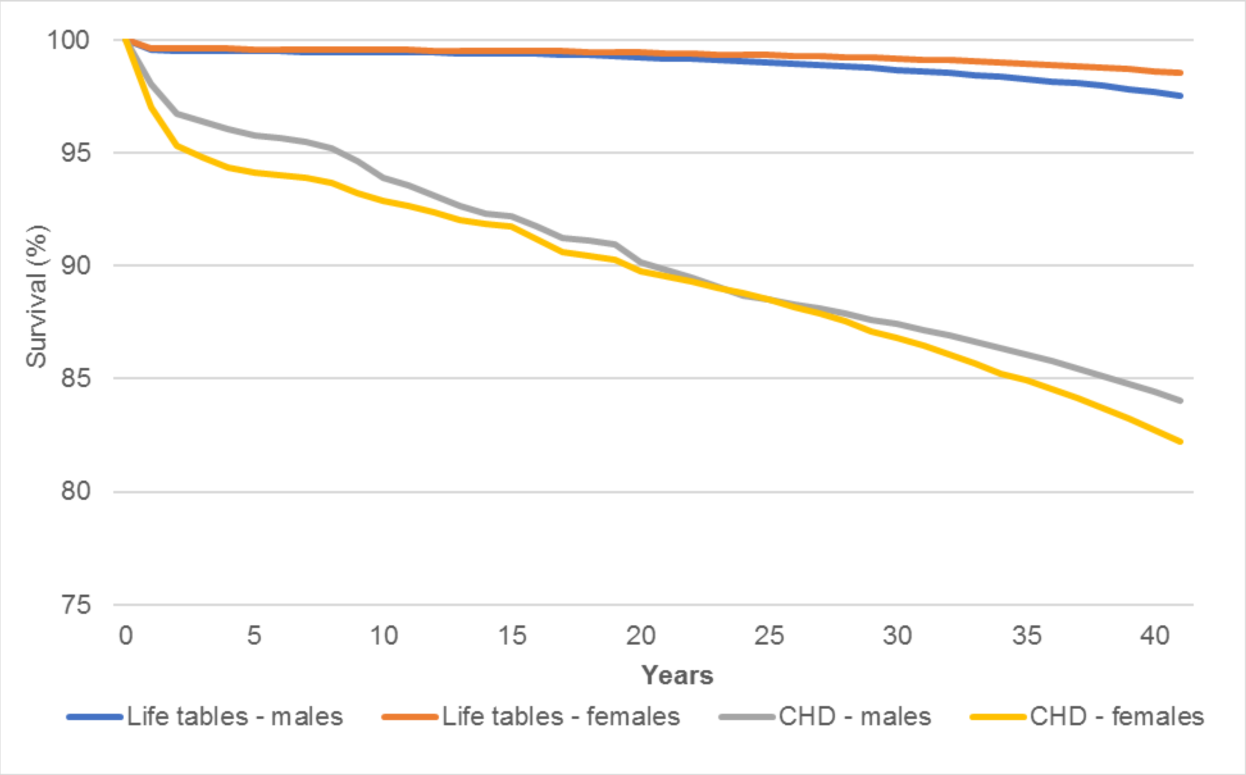

Fig I Life tables vs. CHD survival in the UK
