# Supplementary material for: Congenital heart defect repair with ADAPT tissue engineered pericardium scaffold: An early-stage health economic model
Source: PLoS One. 2018 Sep 27;13(9):e0204643. doi: 10.1371/journal.pone.0204643 (PMC6160133; doi:10.1371/journal.pone.0204643)
Supplement: S4 File — (PDF) [file pone.0204643.s004.pdf]

Aortic valve stenosis base-case results

Our model predicts that when used for the indication of aortic valve stenosis, CardioCel led to less cost and generated additional life years and quality-adjusted life year for the period of 40 years after index surgery and dominated all three other patch types.

Table A Results of cost-effectiveness analysis for base-case in aortic valve stenosis

	Cost, £	Δ Cost, £	LY	Δ LY	QALY	Δ QALY	ICERs
**CardioCel vs Xenogeneic patches**							
Xenogeneic patches	32419		33.605		28.319		
CardioCel	32337	-82	33.623	0.0177	28.335	0.0153	Dominant
**CardioCel vs Autologous patches**							
Autologous patches	32393		33.611		28.324		
CardioCel	32337	-56	33.623	0.0122	28.335	0.0105	Dominant
**CardioCel vs Synthetic patches**							
Synthetic patches	32471		33.594		28.310		
CardioCel	32337	-134	33.623	0.0291	28.335	0.0251	Dominant

Legend: LY – life years. QALY – quality adjusted life years, ICER – incremental cost-effectiveness ratio.

However, differences in generated additional life years and quality-adjusted life years are very small and therefore the incremental cost per reoperation averted was used as the adequate measure of effectiveness in this case.

Table B Results of cost-effectiveness analysis - incremental cost per reoperation averted in aortic valve stenosis

	Cost, £	Δ Cost, £	RI	RA	ICER, £/RA
**CardioCel vs Xenogeneic patches**					
Xenogeneic patches	32419		0.609		
CardioCel	32337	-82	0.599	0.010	Dominant
**CardioCel vs Autologous patches**					

Autologous patches	32393		0.606		
CardioCel	32337	-56	0.599	0.007	Dominant
**CardioCel vs Synthetic patches**					
Synthetic patches	32471		0.615		
CardioCel	32337	-134	0.599	0.016	Dominant

Legend: RI – reoperation incidence, RA – reoperation averted

According to the model results, CardioCel led to the smaller reoperation incidence by 1% in comparison with xenogeneic patches, 0.7% in comparison with autologous patches, and 1.6% in comparison with synthetic patches.

As expected short-term operative mortality and the cost of reoperation had the biggest impact on the results. Utility values for CHD disability as well as cost values of same parameters are one of the most important factors in this analysis irrespective of chosen comparators.

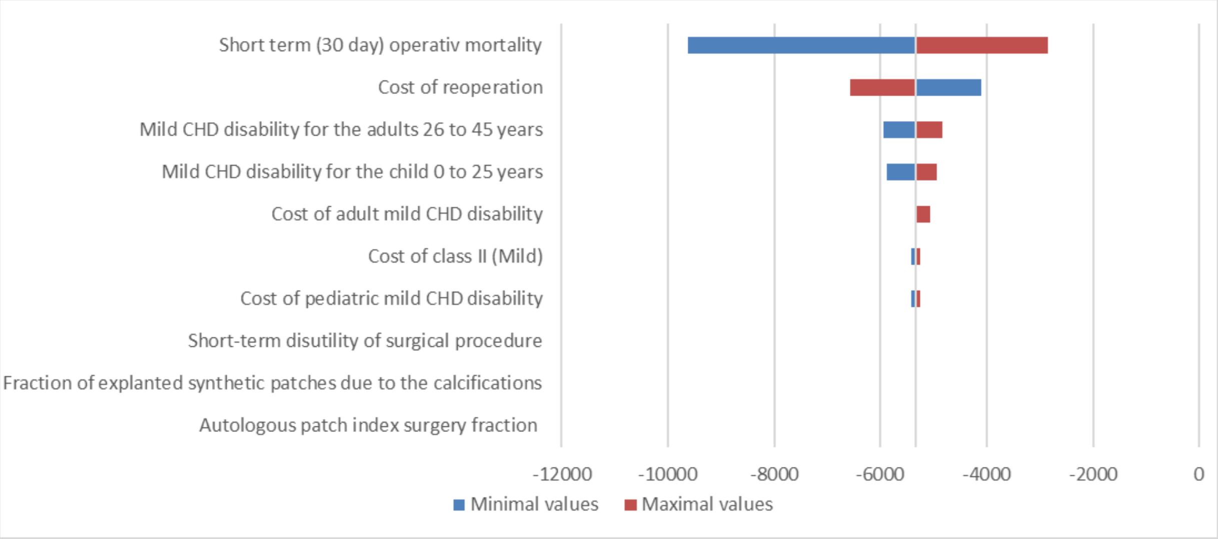

Fig A Tornado diagram for CardioCel/Xenogeneic patches in AS

Probabilistic sensitivity analysis demonstrated that CardioCel produced clinical benefits (additional QALYs) in all patients, and had a cost saving effect in all cases. Those trends stay stable in all 10,000 repeated iterations in which all included parameters were varied according to their distributions.

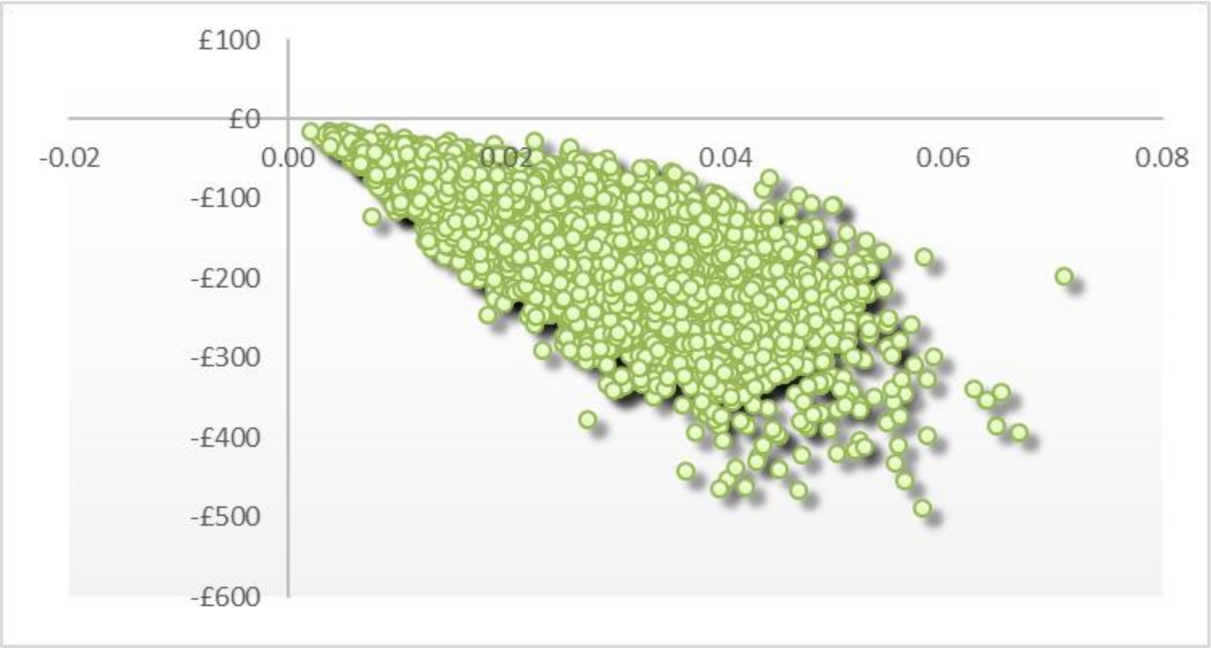

Fig B Cost-effectiveness acceptability plane for ICER

(CardioCel/Xenogeneic patches)

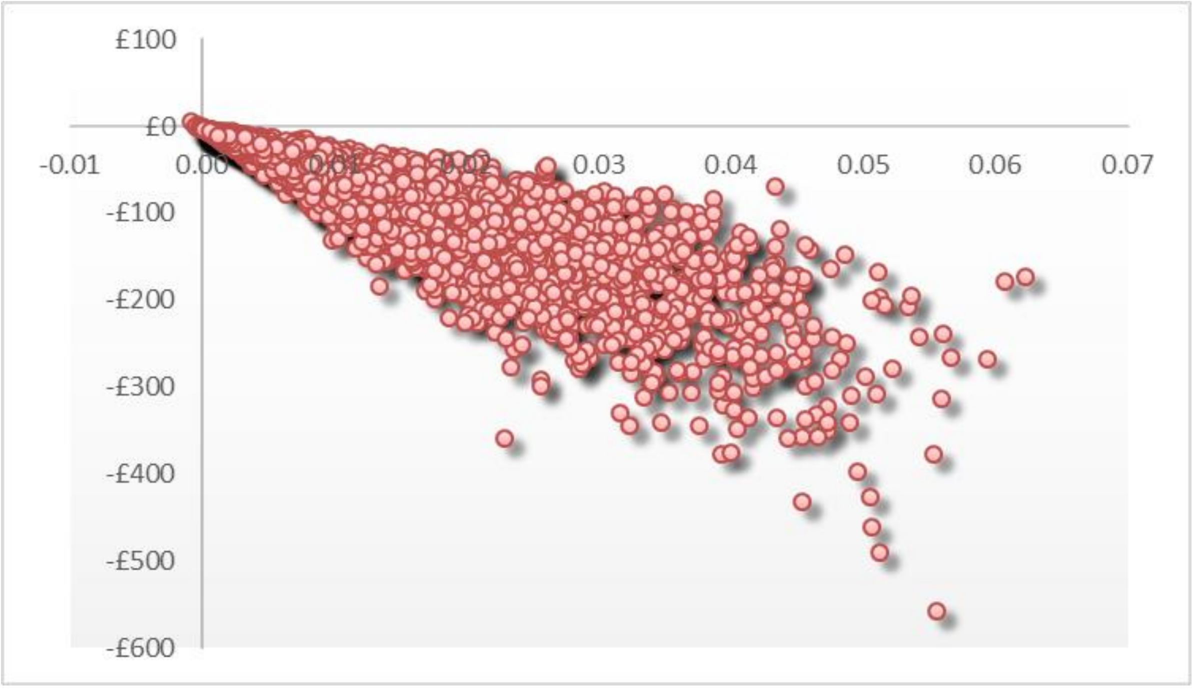

Fig C Cost-effectiveness acceptability plane for ICER

(CardioCel/Autologous patches)

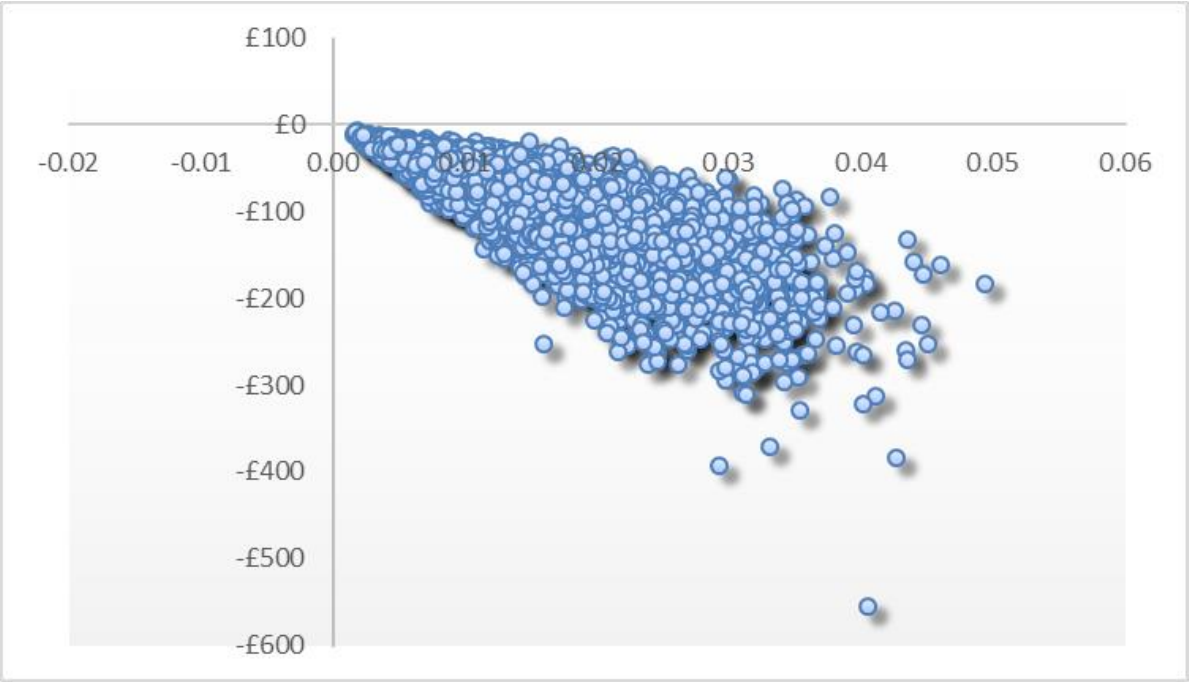

Fig D Cost-effectiveness acceptability plane for ICER (CardioCel/Synthetic patches)

Atrioventricular septum defect base-case results

For the AVSD indication, our model estimated that CardioCel led to less cost and generated additional life years and quality-adjusted life year for the period of 40 years after index surgery and dominated all three other patch types.

Table C Results of cost-effectiveness analysis for base-case in AVSD

	**Cost, £**	**Δ Cost, £**	**LYG**	**Δ LYG**	**QALY**	**Δ QALY**	**ICER, £/QALY**
**CardioCel vs Xenogeneic patches**							
CardioCel	39714		35.303		23.706		
Xenogeneic patches	40086	-372	35.285	0.0183	23.692	0.0136	Dominates
**CardioCel vs Autologous patches**							
CardioCel	39714		35.303		23.706		
Autologous patches	39966	-251	35.291	0.0124	23.697	0.0092	Dominates
**CardioCel vs Synthetic patches**							
CardioCel	39714		35.303		23.706		
Synthetic patches	40330	-616	35.273	0.0302	23.683	0.0225	Dominates

Legend: LY – life years. QALY – quality adjusted life years, ICER – incremental cost-effectiveness ratio.

However, as in all other analyzed diseases, the differences in generated additional life years and quality-adjusted life years are very small and therefore the incremental cost per reoperation averted was used as an adequate measure of effectiveness in this case.

Table D Results of cost-effectiveness analysis - incremental cost per reoperation averted in AVSD

	**Cost, £**	**Δ Cost, £**	**RI**	**RA**	**ICER, £/RA**
**CardioCel vs Xenogeneic patches**					
CardioCel	39714		0.202		
Xenogeneic patches	40086	-372	0.238	0.036	Dominates
**CardioCel vs Autologous patches**					
CardioCel	39714		0.202		
Autologous patches	39966	-251	0.226	0.024	Dominates
**CardioCel vs Synthetic patches**					
CardioCel	39714		0.202		
Synthetic patches	40330	-616	0.261	0.059	Dominates

Legend: RI – reoperation incidence, RA – reoperation averted

According to those projections, CardioCel led to the smaller reoperation incidence by 3.6% in comparison with xenogeneic patches, 2.4% in comparison with autologous patches and 5.9% in comparison with synthetic patches.

One-way sensitivity analysis demonstrates similar results as in the case of aortic valve stenosis.

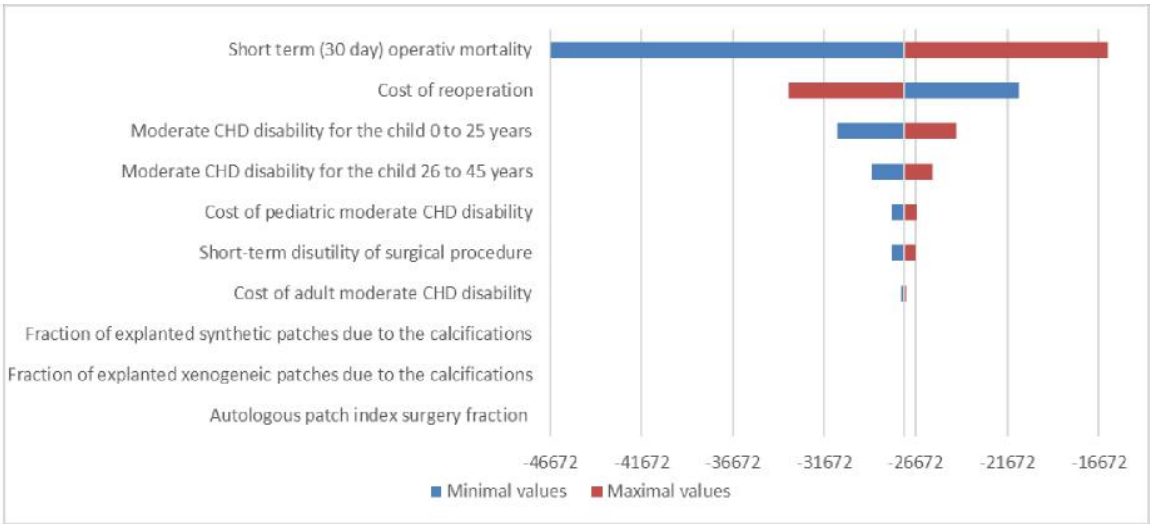

Fig E Tornado diagram for CardioCel/Xenogeneic patches in AVSD

Probabilistic sensitivity analysis demonstrated that CardioCel produced clinical benefits (additional QALYs) in all patients, and had a cost saving effect in the all of the cases.

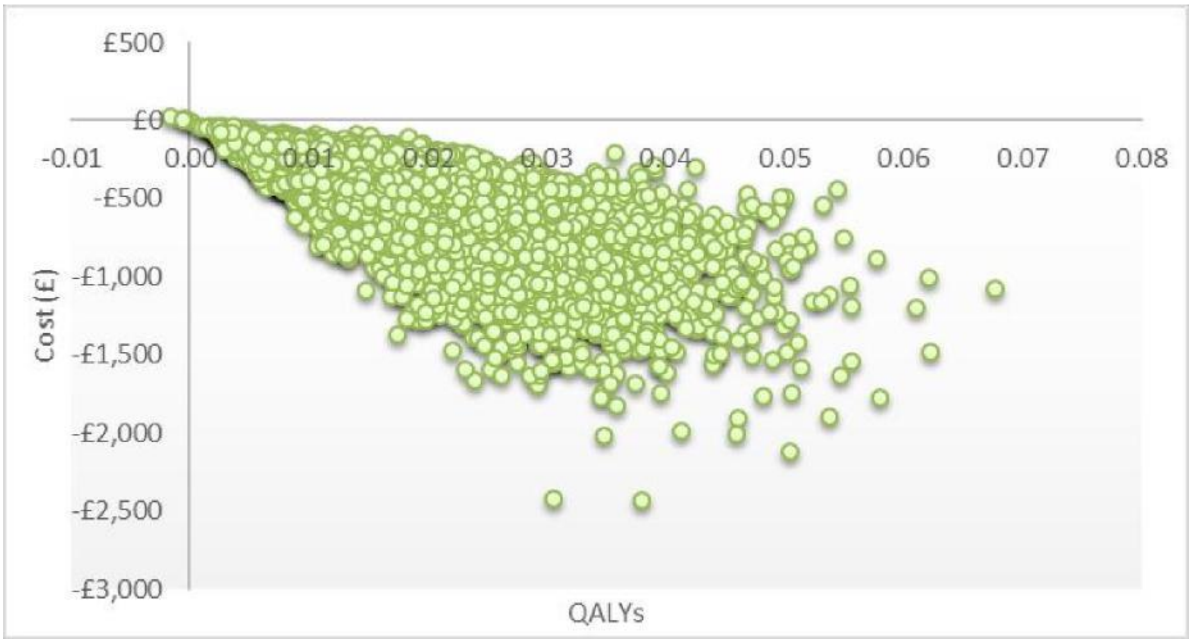

Fig F Cost-effectiveness acceptability plane for ICER

(CardioCel/Xenogeneic patches)

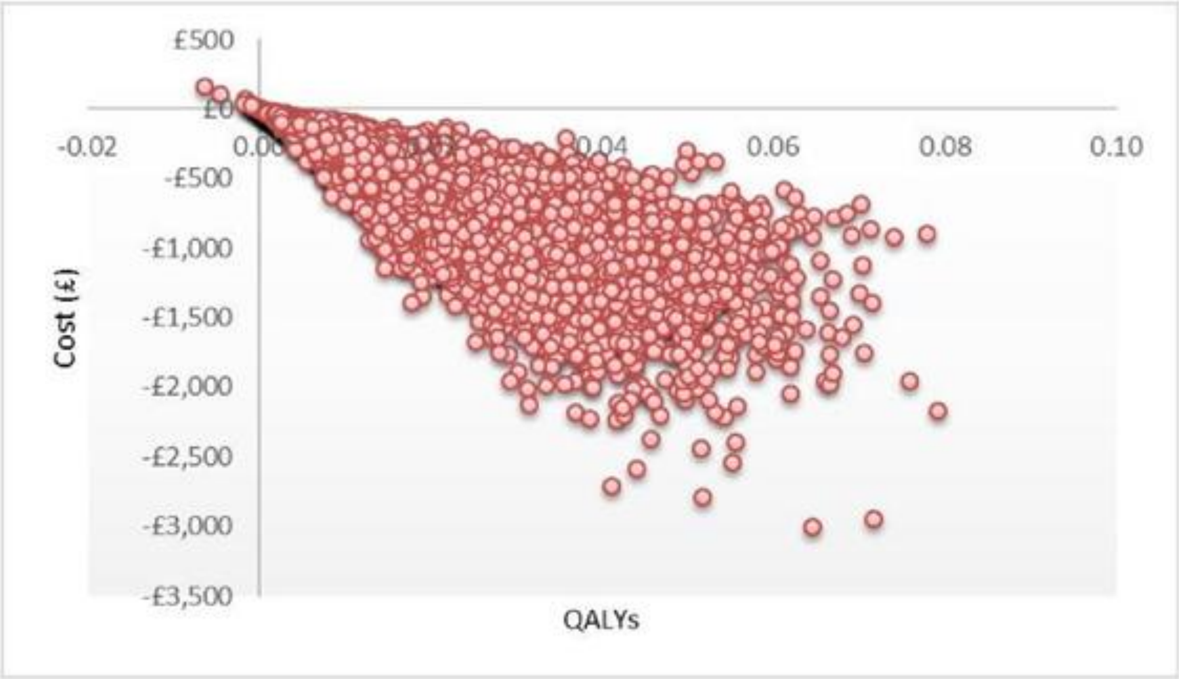

Fig G Cost-effectiveness acceptability plane for ICER

(CardioCel/Autologous patches)

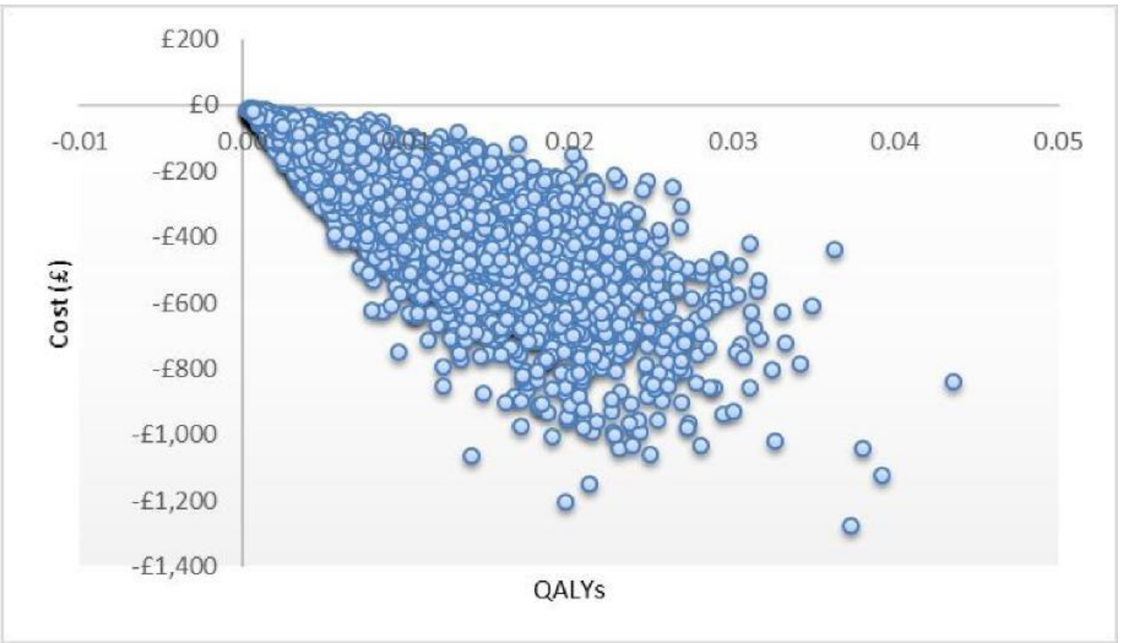

Fig H Cost-effectiveness acceptability plane for ICER (CardioCel/Synthetic patches)

Ventricular septum defect base-case results

In the case of VSD, our model predicts very small difference in life years gained and QALY gained between the types of patch used, not allowing reasonable analysis. In this case, the incremental cost per reoperation averted is used as the main outcome measure instead of QALYs.

According to our model, CardioCel dominates all patch types leading to the 0.1% of averted surgeries in comparison with xenogeneic patches and autologous patches, and 0.3% averted reoperations in comparison with synthetic patches.

Table E Results of cost-effectiveness analysis - incremental cost per reoperation averted

	Cost, £	Δ Cost, £	RI	RA	ICER, £/RA
**CardioCel vs Xenogeneic patches**					
CardioCel	14755		0.167		
Xenogeneic patches	14759	-4	0.168	0.001	Dominates
**CardioCel vs Autologous patches**					
CardioCel	14755		0.167		
Autologous patches	14758	-2	0.167	0.001	Dominates
**CardioCel vs Synthetic patches**					
CardioCel	14755		0.167		
Synthetic patches	14762	-7	0.169	0.003	Dominates

Legend: RI – reoperation incidence, RA – reoperation averted

Probabilistic sensitivity analysis demonstrated that CardioCel produced clinical benefits (additional QALYs) in all patients, and had a cost saving effect in the all of the cases. Those trends stay stable in all 10,000 repeated iterations in which all included parameters were varied according to their distributions.

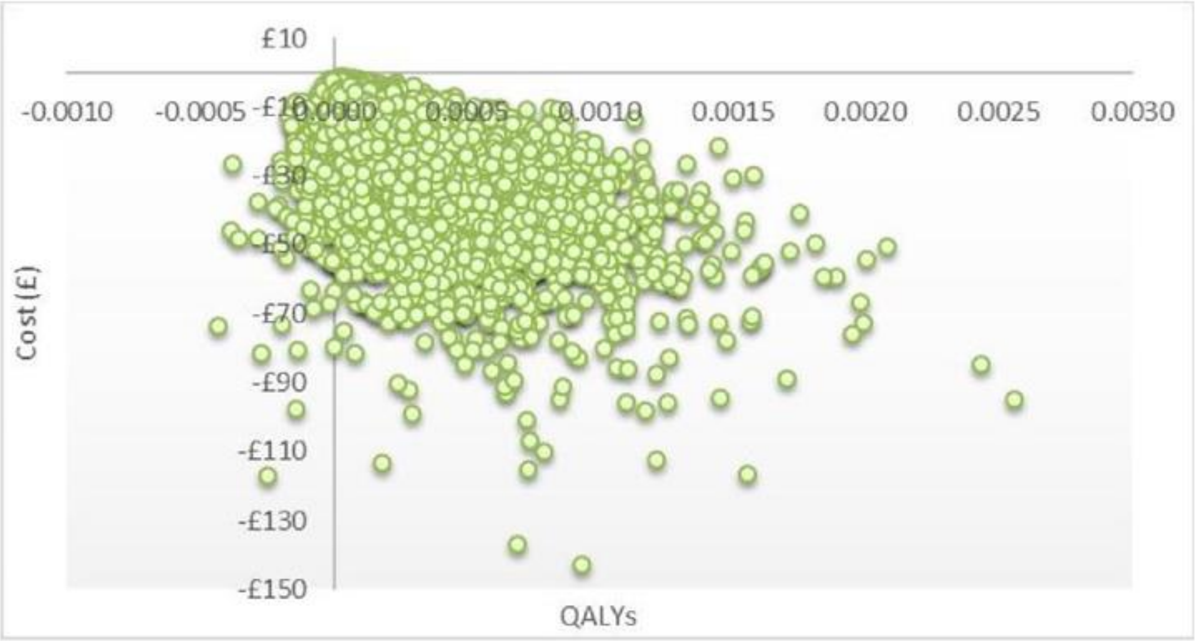

Fig I Cost-effectiveness acceptability plane for incremental cost per reoperation averted (CardioCel/Xenogeneic patches)

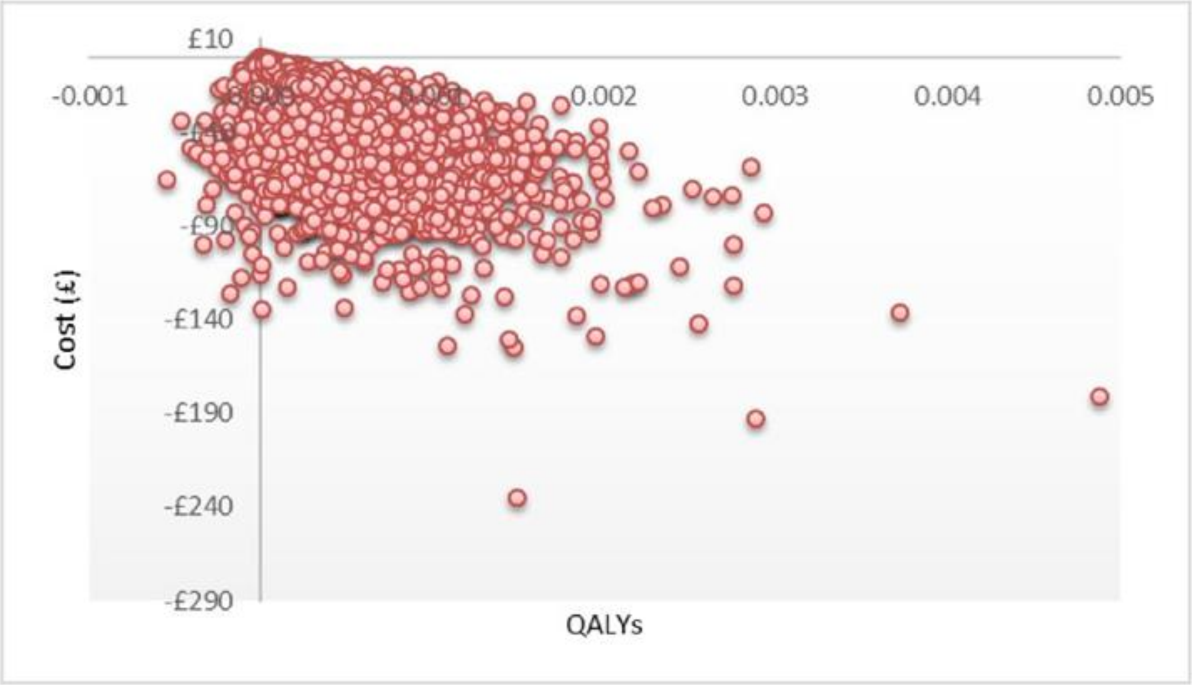

Fig K. Cost-effectiveness acceptability plane for incremental cost per reoperation averted (CardioCel/Autologous patches)

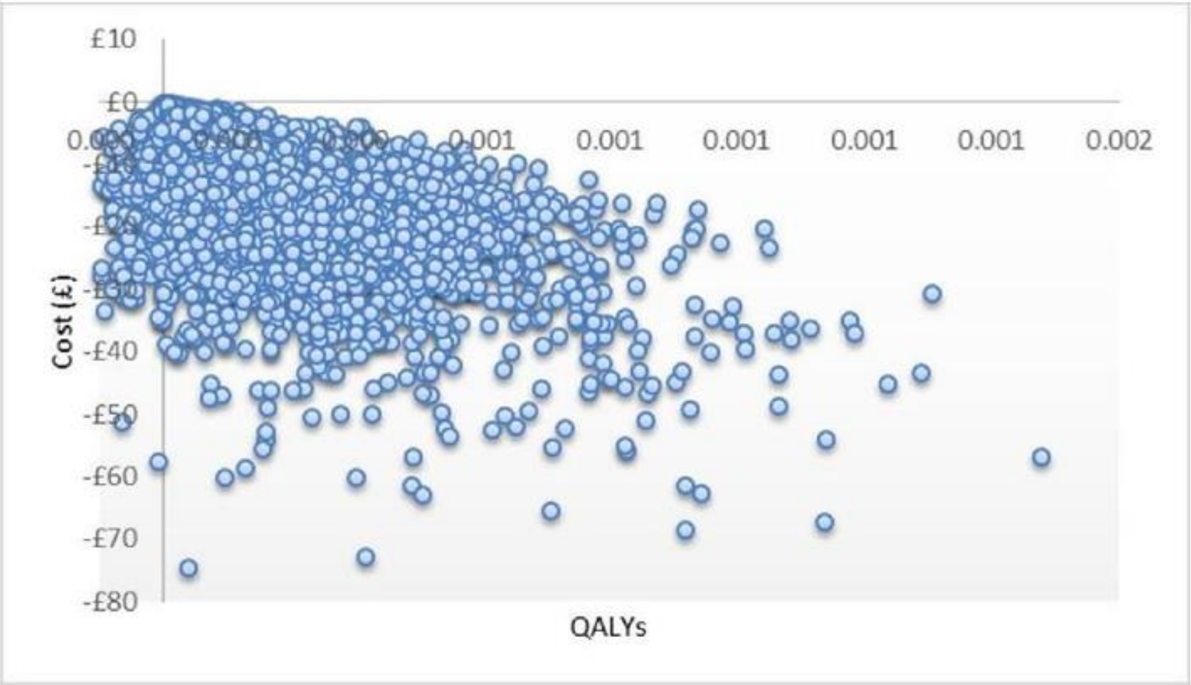

Fig L Cost-effectiveness acceptability plane for incremental cost per reoperation averted (CardioCel/Synthetic patches)

Tetralogy of Fallot base-case results

According to the results predicted by our model, patients with tetralogy of Fallot, as in other indications in this analysis, CardioCel led to less cost and more additional life years and quality-adjusted life year but that effect can be considered as very marginal even though it is evident that CardioCel dominated all three other patch types.

Table F Results of cost-effectiveness analysis for base-case in ToF

	**Cost, £**	**Δ Cost, £**	**LYG**	**Δ LYG**	**QALY**	**Δ QALY**	**ICER, £/QALY**
**CardioCel vs Xenogeneic patches**							
CardioCel	37438		34.626		23.953		
Xenogeneic patches	37504	-66	34.625	0.0015	23.952	0.0012	Dominates
**CardioCel vs Autologous patches**							
CardioCel	37438		34.626		23.953		
Autologous patches	37576	-138	34.623	0.0031	23.950	0.0025	Dominates
**CardioCel vs Synthetic patches**							
CardioCel	37438		34.626		23.953		
Synthetic patches	37591	-153	34.623	0.0034	23.950	0.0027	Dominates

Legend: LY – life years. QALY – quality adjusted life years, ICER – incremental cost-

effectiveness ratio.

Again, better insight into the true economic values of CardioCel can be measured by using incremental cost per reoperation averted.

Table G Results of cost-effectiveness analysis - incremental cost per reoperation averted in aortic valve stenosis

	Cost. £	Δ Cost, £	RI	RA	ICER. £/RA
**CardioCel vs Xenogeneic patches**					
CardioCel	37438		0.125		
Xenogeneic patches	37504	-66	0.132	0.007	Dominates
**CardioCel vs Autologous patches**					
CardioCel	37438		0.125		
Autologous patches	37576	-138	0.139	0.014	Dominates
**CardioCel vs Synthetic patches**					
CardioCel	37438		0.125		
Synthetic patches	37591	-153	0.141	0.015	Dominates

Legend: RI – reoperation incidence. RA – reoperation averted

Projections from our model indicate that CardioCel led to the smaller reoperation incidence for 0.7% in comparison with xenogeneic patches, 1.4% in comparison with autologous patches, and 1.5% in comparison with synthetic patches.

The one-way sensitivity analyses are in line with previous sensitivity analysis in other indications confirming short-term mortality and cost of reoperations as important parameters.

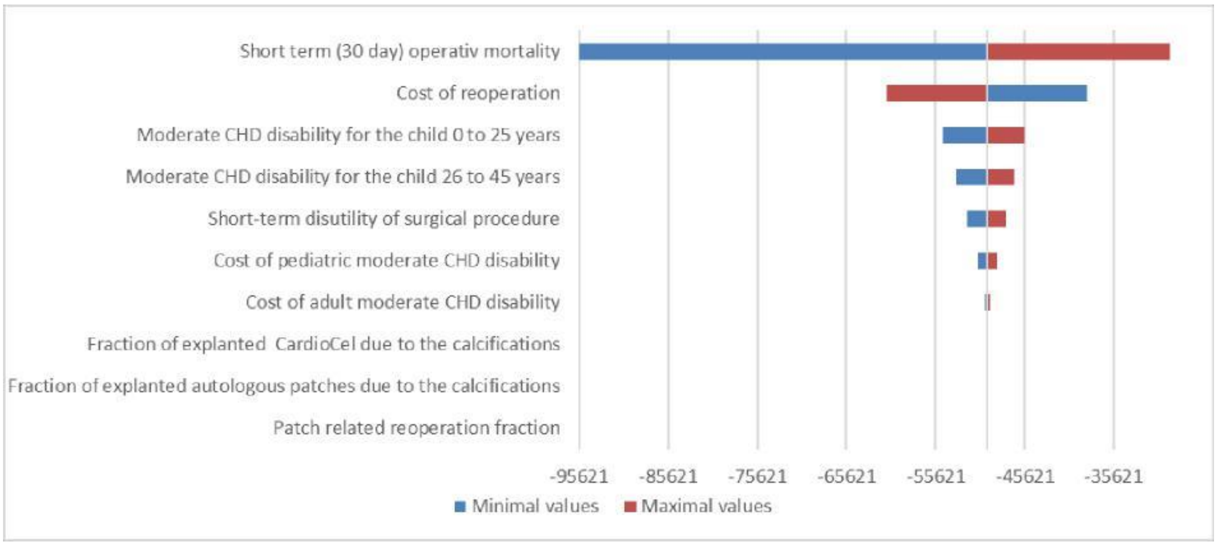

Fig M Tornado diagram for CardioCel/Xenogeneic patches in ToF

Probabilistic sensitivity analysis demonstrated that CardioCel may produce clinical benefits (additional QALYs) in the majority of iterations with cost saving effect.

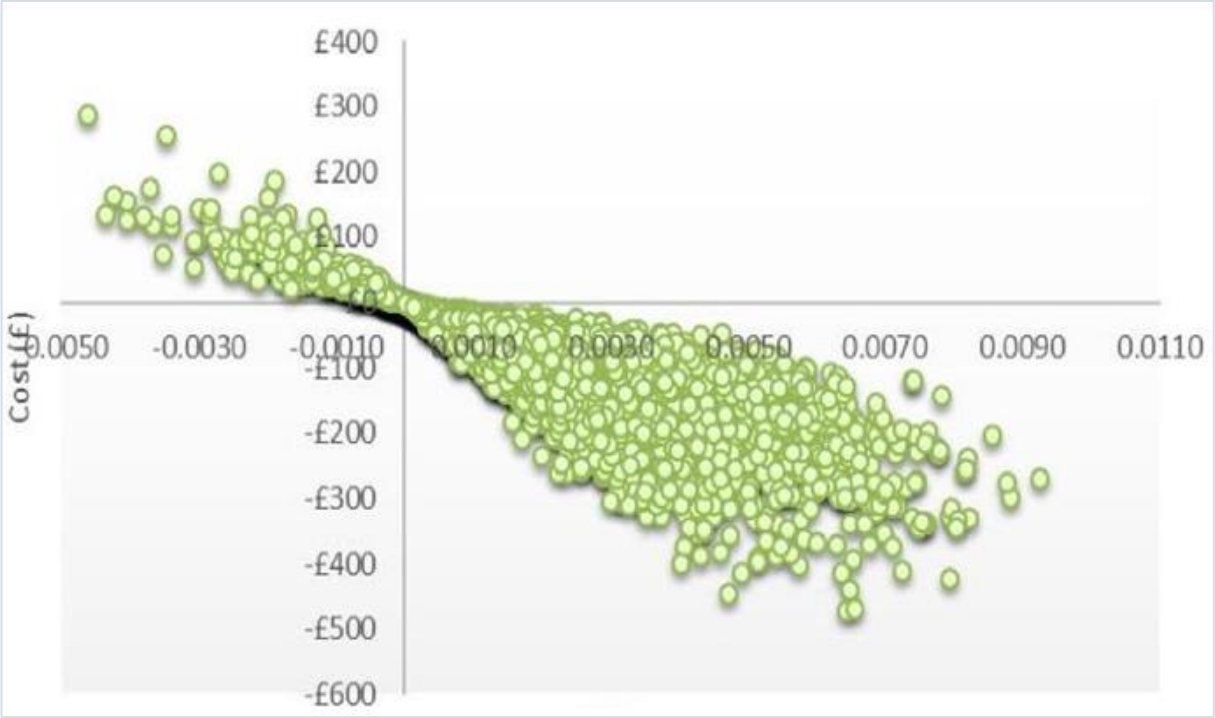

Fig N Cost-effectiveness acceptability plane for ICER

(CardioCel/Xenogeneic patches)

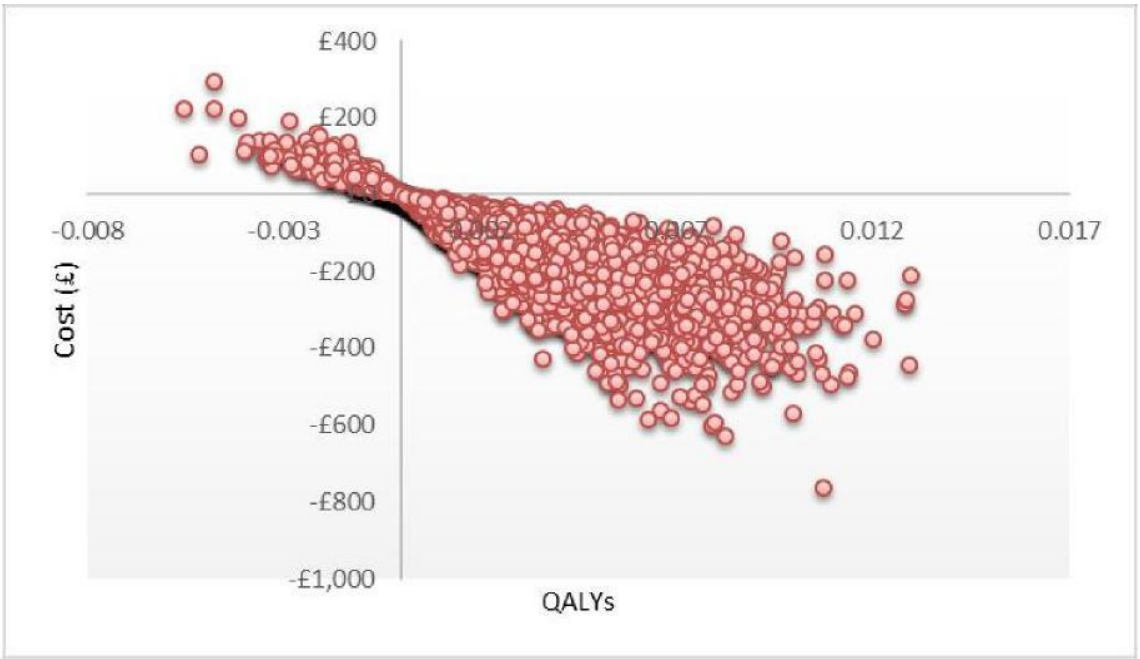

Fig O Cost-effectiveness acceptability plane for ICER

(CardioCel/Autologous patches)

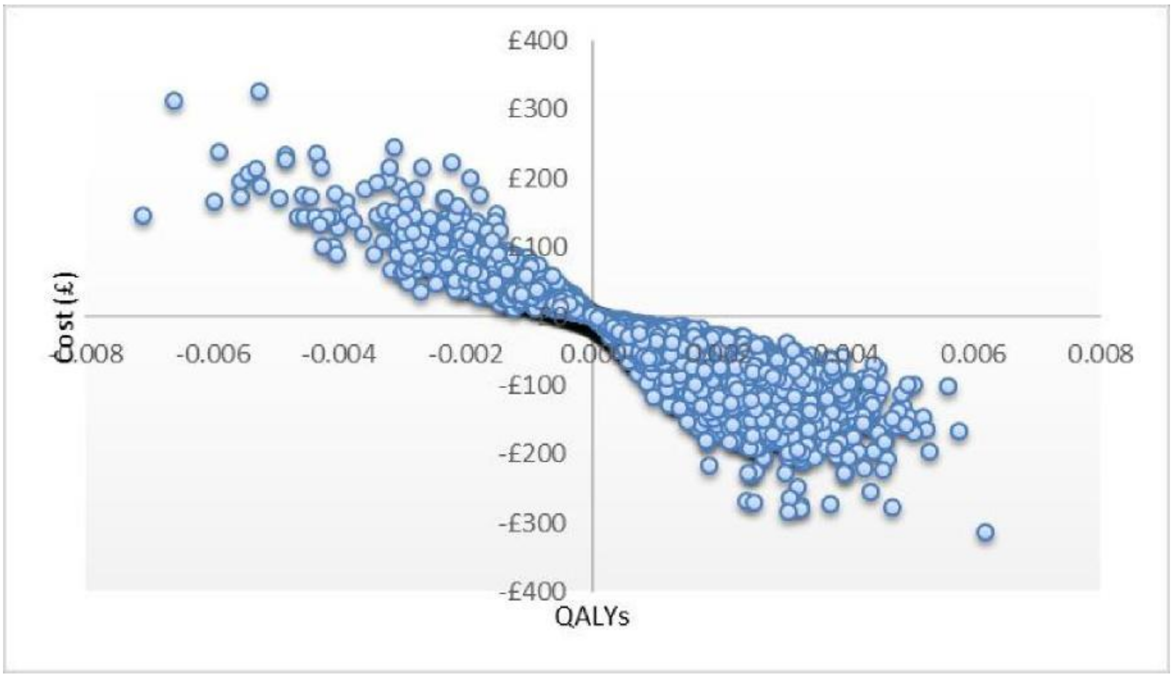

Fig P Cost-effectiveness acceptability plane for ICER (CardioCel/Synthetic patches)

Transposition of great arteries base-case results

According to model predictions the trend of cost/saving among CHD patients is present even in the more severe indications, such as TGA. In this indication, CardioCel led to less cost and generated additional life years and quality-adjusted life year of a lifetime of the cohorts and dominated all three other patch types.

Table H Results of cost-effectiveness analysis for base-case in TGA

	**Cost. £**	**Δ Cost £**	**LYG**	**Δ LYG**	**QALY**	**Δ QALY**	**ICER. £/QALY**
**CardioCel vs Xenogeneic patches**							
CardioCel	48962		31.026		11.411		
Xenogeneic patches	49063	-101	31.018	0.0082	11.407	0.0037	Dominates
**CardioCel vs Autologous patches**							
CardioCel	48962		31.026		11.411		
Autologous patches	49019	-58	31.021	0.0046	11.409	0.0021	Dominates
**CardioCel vs Synthetic patches**							
CardioCel	48962		31.026		11.411		
Synthetic patches	49153	-191	31.011	0.0154	11.404	0.0069	Dominates

Legend: LY – life years. QALY – quality adjusted life years, ICER – incremental cost-effectiveness ratio.

Additionally, due to the small differences in generated additional life years and quality-adjusted life years' incremental cost per reoperation averted was calculated.

Table I Results of cost-effectiveness analysis - incremental cost per reoperation averted in TGA

	**Cost. £**	**Δ cost. £**	**RI**	**RA**	**ICER. £/RA**
**CardioCel vs Xenogeneic patches**					
CardioCel	48962		0.193		
Xenogeneic patches	49063	-101	0.207	0.013	Dominates
**CardioCel vs Autologous patches**					
CardioCel	48962		0.193		
Autologous patches	49019	-58	0.201	0.008	Dominates
**CardioCel vs Synthetic patches**					
CardioCel	48962		0.193		
Synthetic patches	49153	-191	0.218	0.025	Dominates

Legend: RI – reoperation incidence. RA – reoperation averted

According to those model estimates, CardioCel led to the smaller reoperation incidence by 1.3% in comparison with xenogeneic patches, 0.8% in comparison with autologous patches, and 2.5% in comparison with synthetic patches.

New variables with high impact on ICER are related to severe CHD disability utility values as well as costs in addition to short-term operative mortality and cost of reoperations.

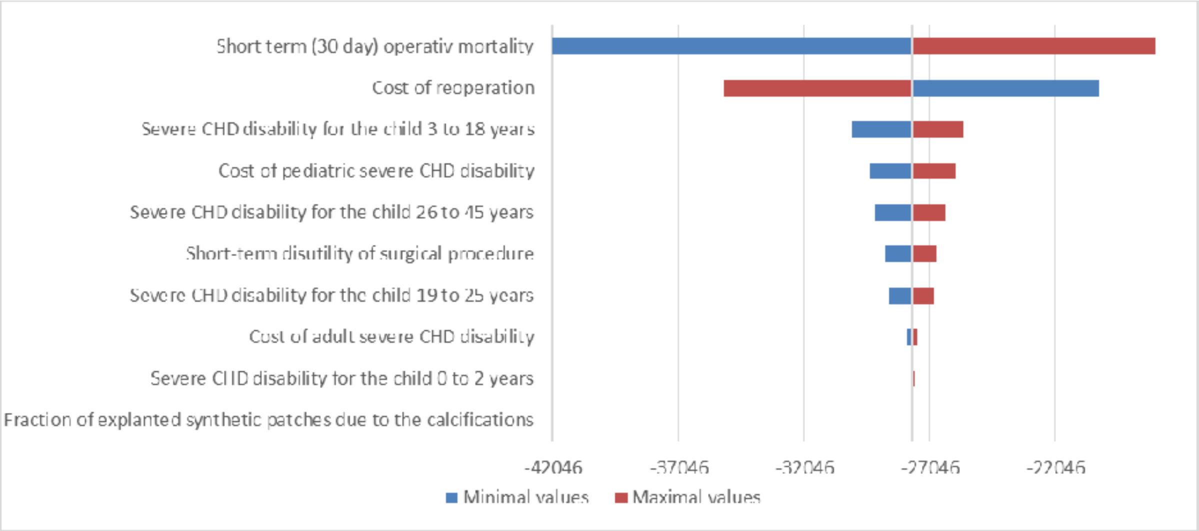

Fig Q Tornado diagram for CardioCel/Xenogeneic patches in TGA

Probabilistic sensitivity analysis demonstrated that CardioCel produced clinical benefits (additional QALYs) and had cost saving effect in the majority of the iterations.

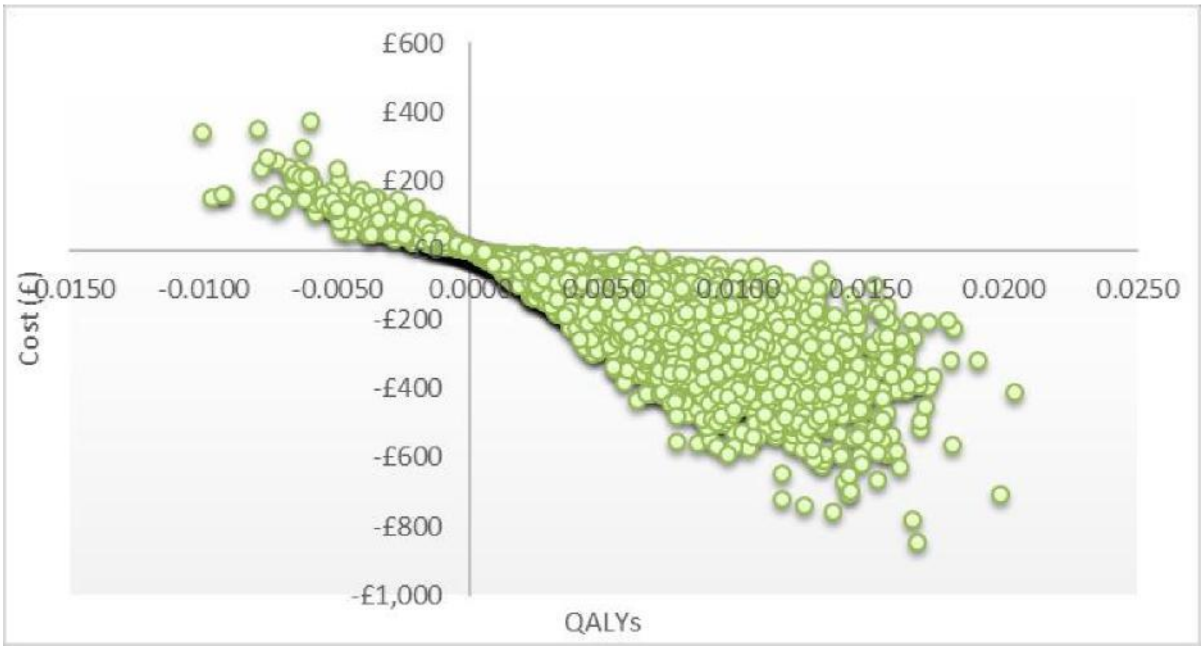

Fig R Cost-effectiveness acceptability plane for ICER

(CardioCel/Xenogeneic patches)

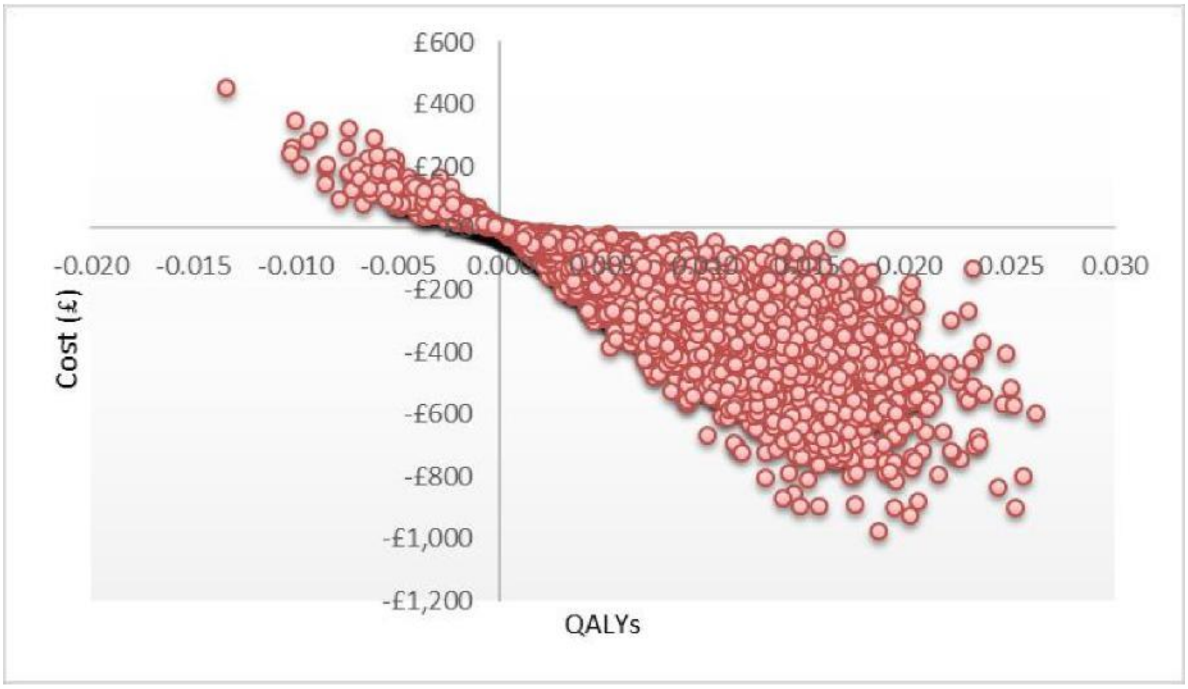

Fig S Cost-effectiveness acceptability plane for ICER

(CardioCel/Autologous patches)

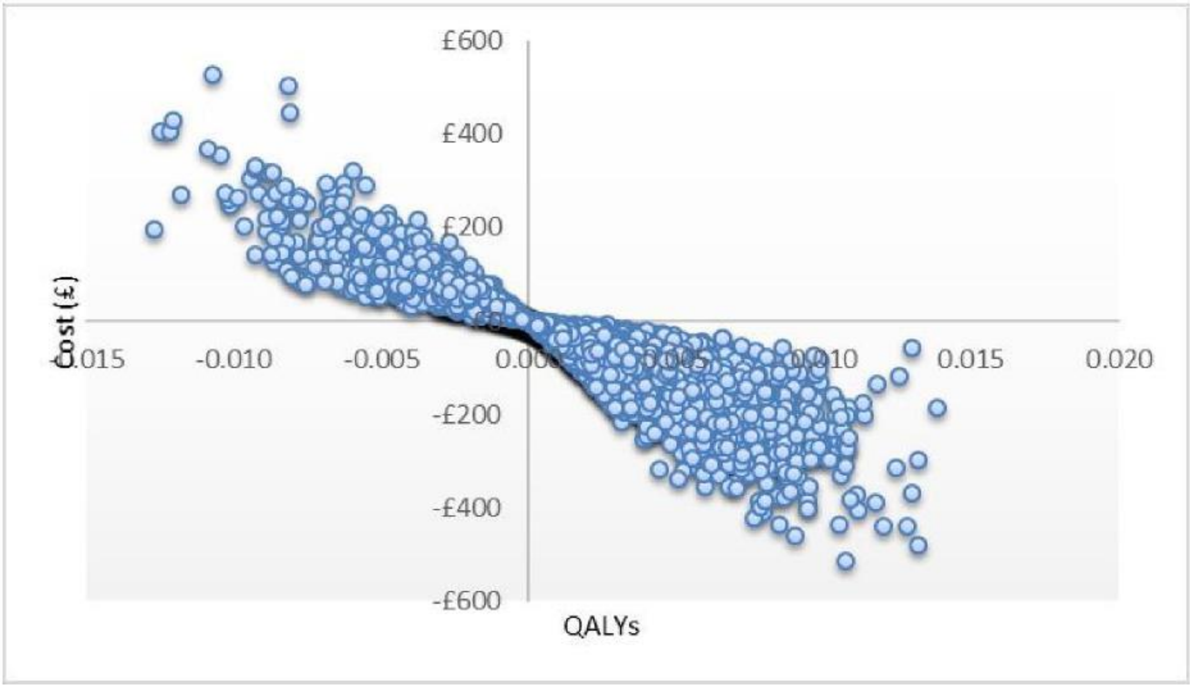

Fig T Cost-effectiveness acceptability plane for ICER (CardioCel/Synthetic patches)

Coarctation of the aorta base-case results

In CoA indication, according to the model estimates, CardioCel led to less cost and generated additional life years and quality-adjusted life year of a lifetime of the cohorts and dominated all three other patch types.

Table J Results of cost-effectiveness analysis for base-case in CoA

	**Cost. £**	**Δ cost. £**	**LYG**	**Δ LYG**	**QALY**	**Δ QALY**	**ICER. £/QALY**
**CardioCel vs Xenogeneic patches**							
CardioCel	44077		36.895		24.792		
Xenogeneic patches	44170	-94	36.892	0.0027	24.790	0.0020	Dominates
**CardioCel vs Autologous patches**							
CardioCel	44077		36.895		24.792		
Autologous patches	44130	-53	36.894	0.0015	24.791	0.0012	Dominates
**CardioCel vs Synthetic patches**							
CardioCel	44077		36.895		24.792		
Synthetic patches	44254	-177	36.890	0.0050	24.789	0.0039	Dominates

Legend: LY – life years. QALY – quality adjusted life years, ICER – incremental cost-effectiveness ratio

In addition, cost per reoperation averted was used as well as in other diseases.

Table K Results of cost-effectiveness analysis - incremental cost per reoperation averted in CoA

	Cost. £	Δ cost. £	RI	RA	ICER. £/RA
**CardioCel vs Xenogeneic patches**					
CardioCel	44077		0.286		
Xenogeneic patches	44170	-94	0.296	0.010	Dominates
**CardioCel vs Autologous patches**					
CardioCel	44077		0.286		
Autologous patches	44130	-53	0.292	0.006	Dominates
**CardioCel vs Synthetic patches**					
CardioCel	44077		0.286		
Synthetic patches	44254	-177	0.305	0.019	Dominates

Legend: RI – reoperation incidence. RA – reoperation averted

According to the model predictions, CardioCel led to the smaller reoperation incidence by 1% in comparison with xenogeneic patches, 0.6% in comparison with autologous patches, and 1.9% in comparison with synthetic patches.

As expected short-term operative mortality and cost of reoperations had the biggest impact on the results.

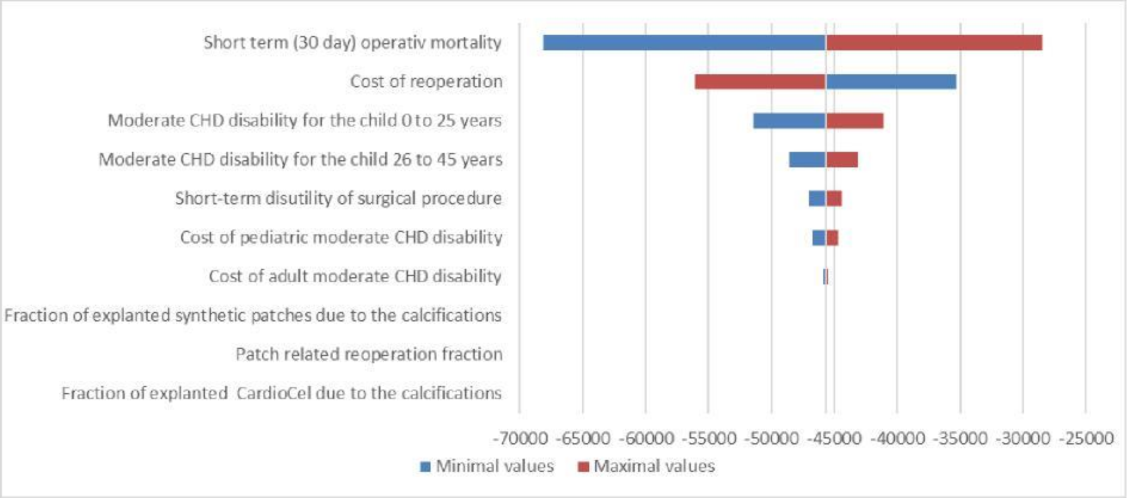

Fig U Tornado diagram for CardioCel/Xenogeneic patches in TGA

Probabilistic sensitivity analysis demonstrated that CardioCel produced clinical benefits (additional QALYs) and had a cost saving effect in majority of the iterations.

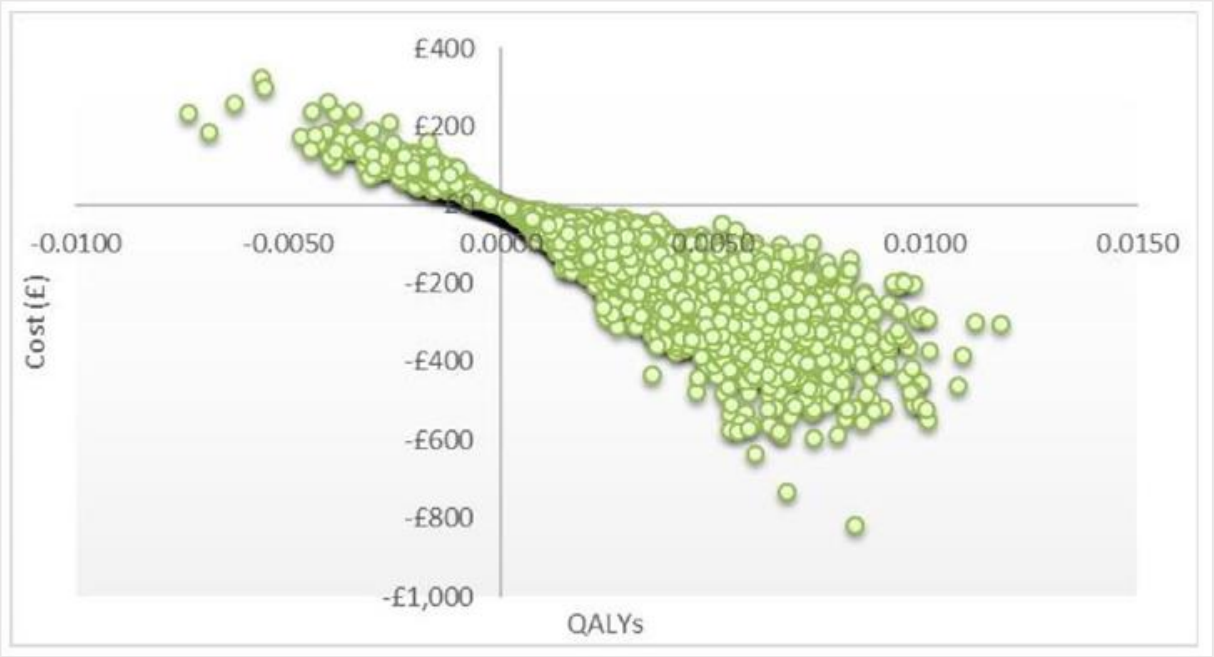

Fig V Cost-effectiveness acceptability plane for ICER

(CardioCel/Xenogeneic patches)

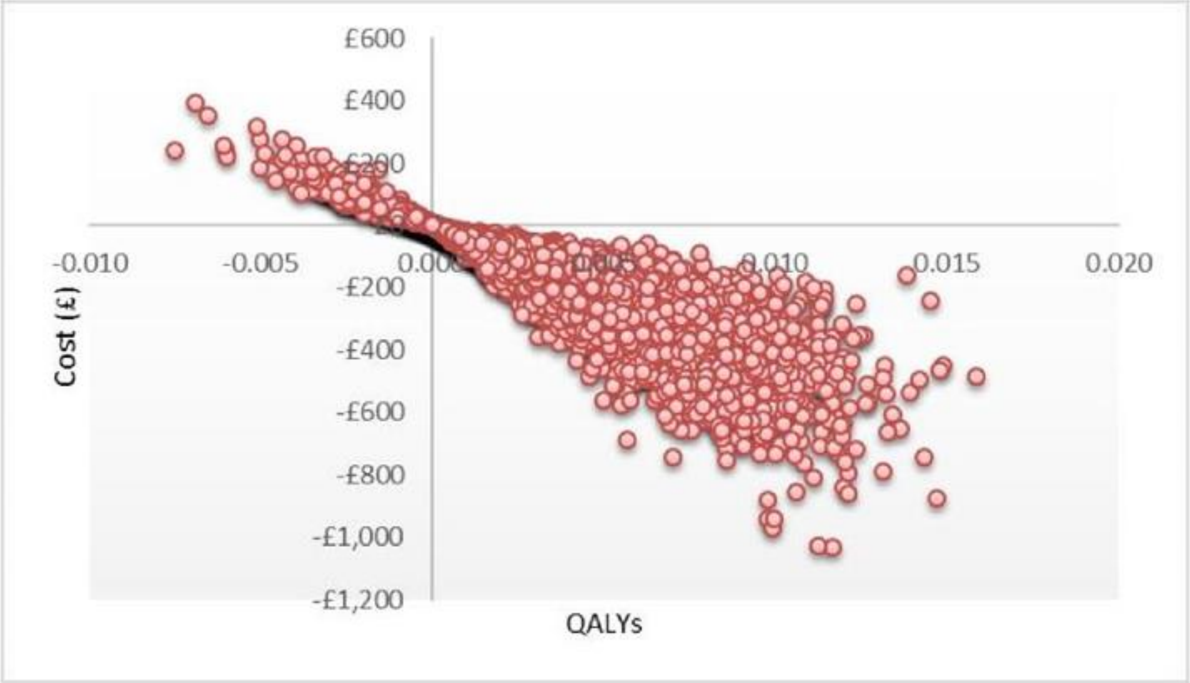

Fig W Cost-effectiveness acceptability plane for ICER

(CardioCel/Autologous patches)

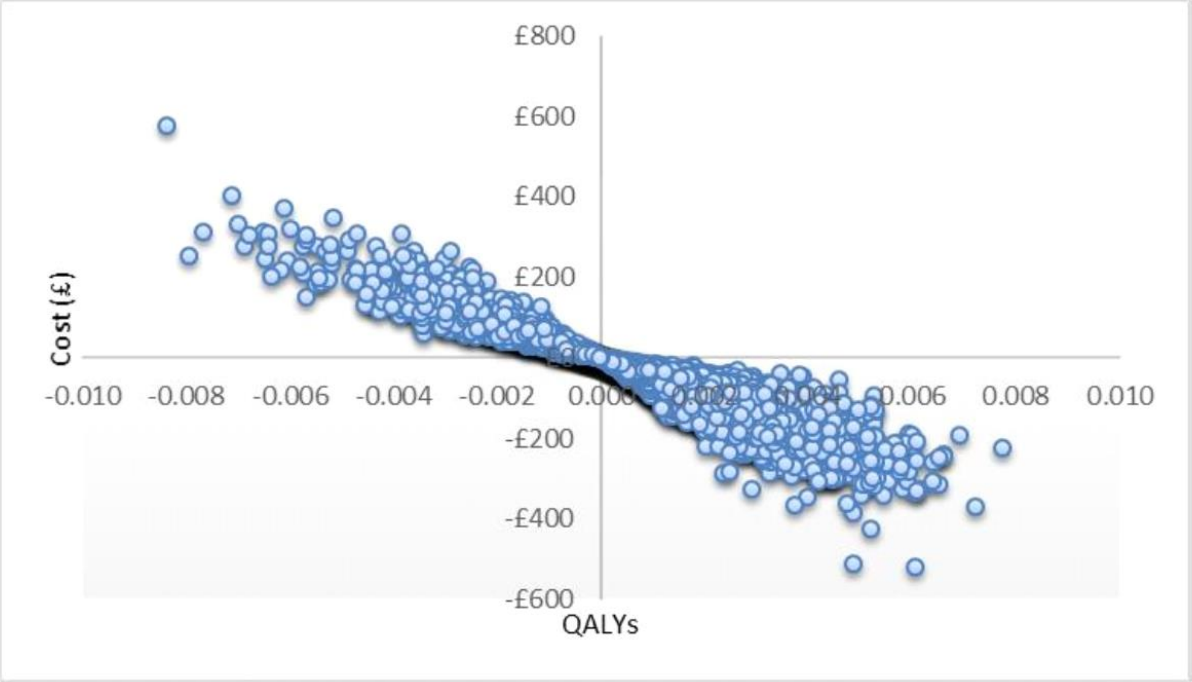

Fig X Cost-effectiveness acceptability plane for ICER (CardioCel/Synthetic patches)

Figure S 24 to S 35 reports the cost breakdowns in all assessed disease according to the model predictions.

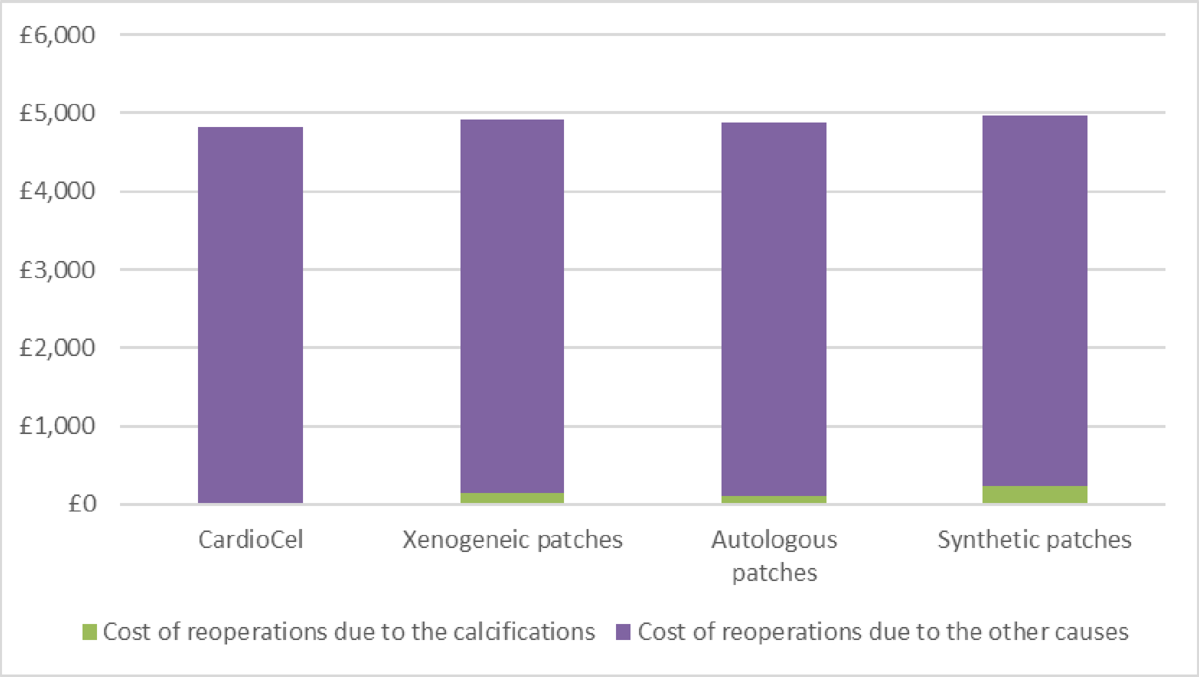

Fig Y Breakdown of cost of reoperations in the aortic valve stenosis cohort

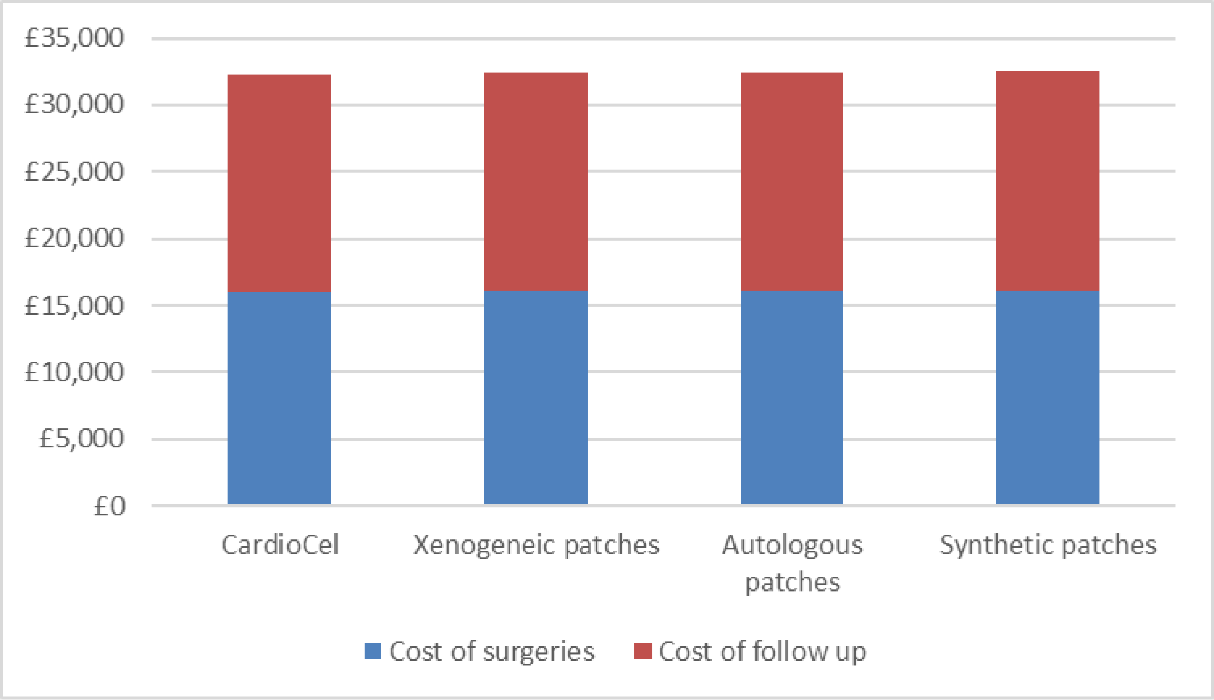

Fig Z Breakdown of cost of index surgery and follow-up in the aortic valve stenosis cohort

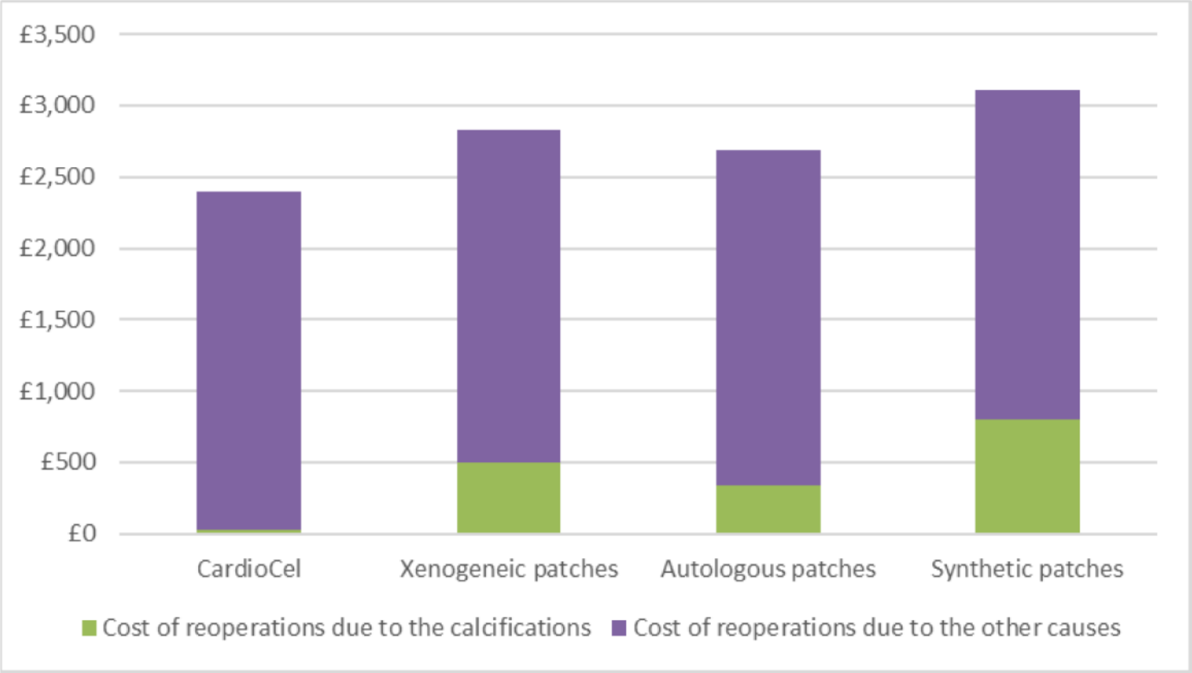

Fig AA Breakdown of cost of reoperations in the atrioventricular septum defect cohort

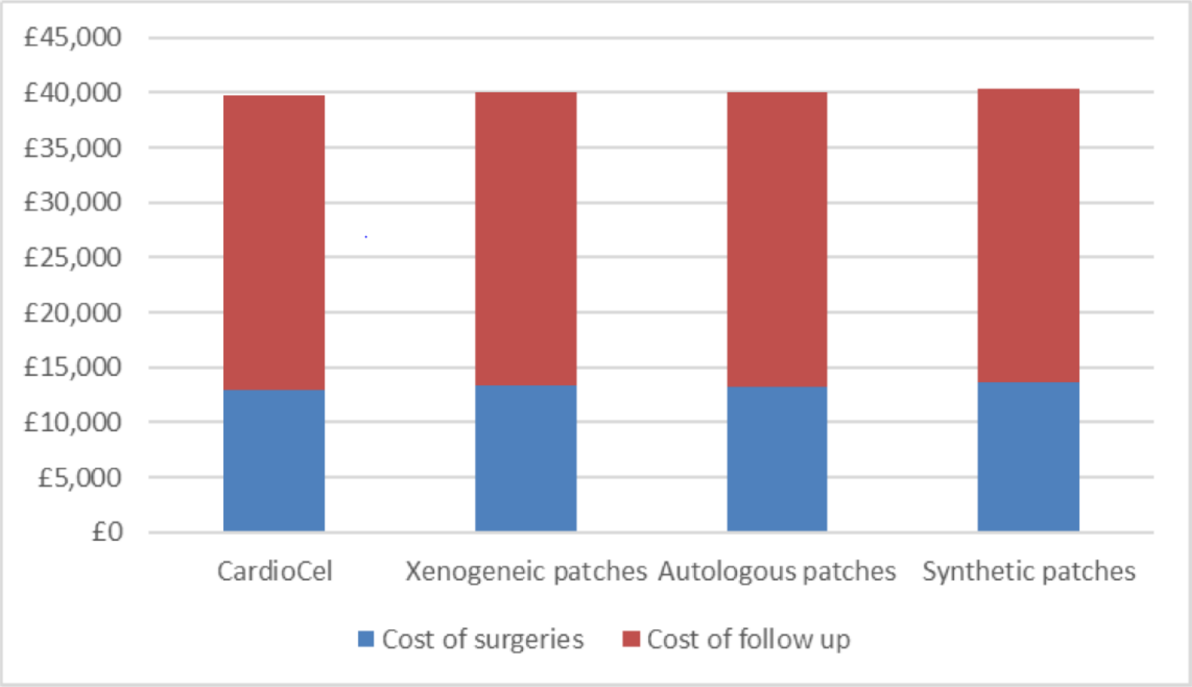

Fig AB Breakdown of cost of index surgery and follow-up in the atrioventricular septum defect cohort

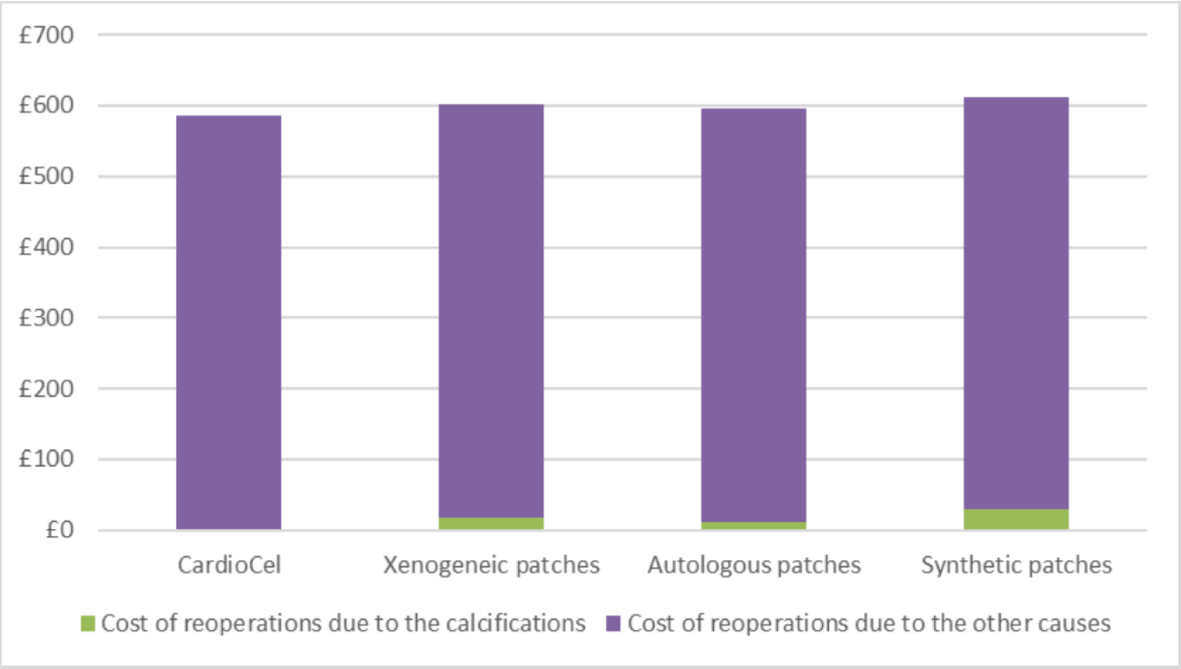

Fig AC Breakdown of cost of reoperations in the ventricular septum defect cohort

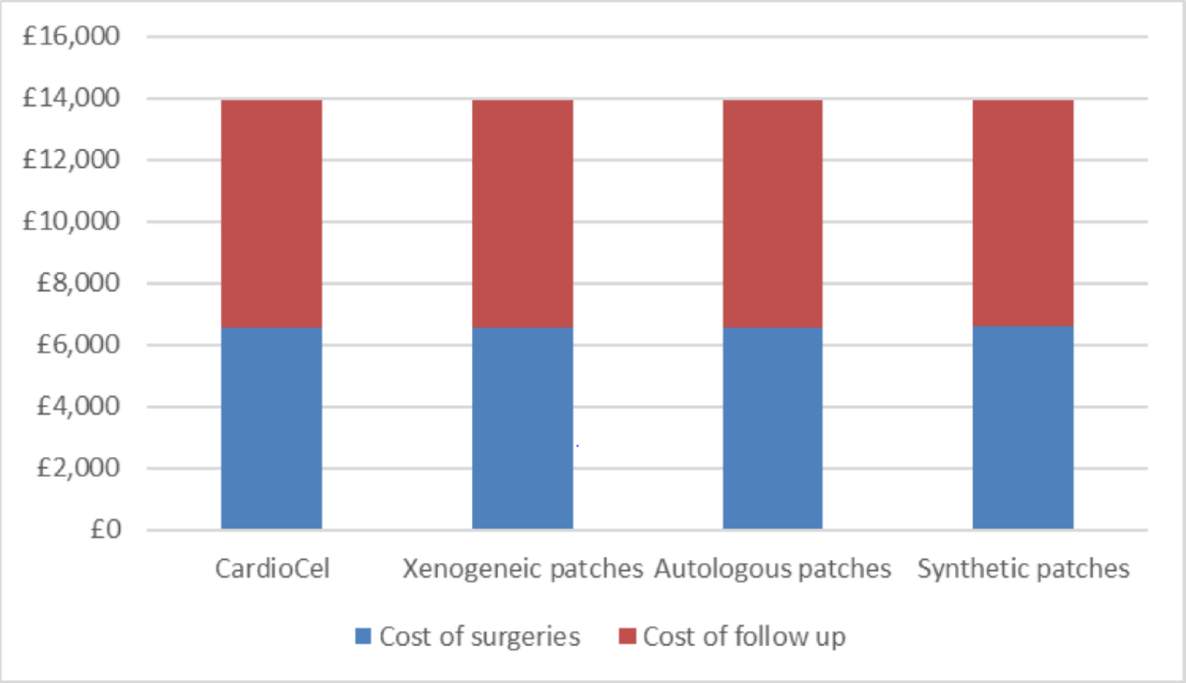

Fig AD Breakdown of cost of index surgery and follow-up in the ventricular septum defect cohort

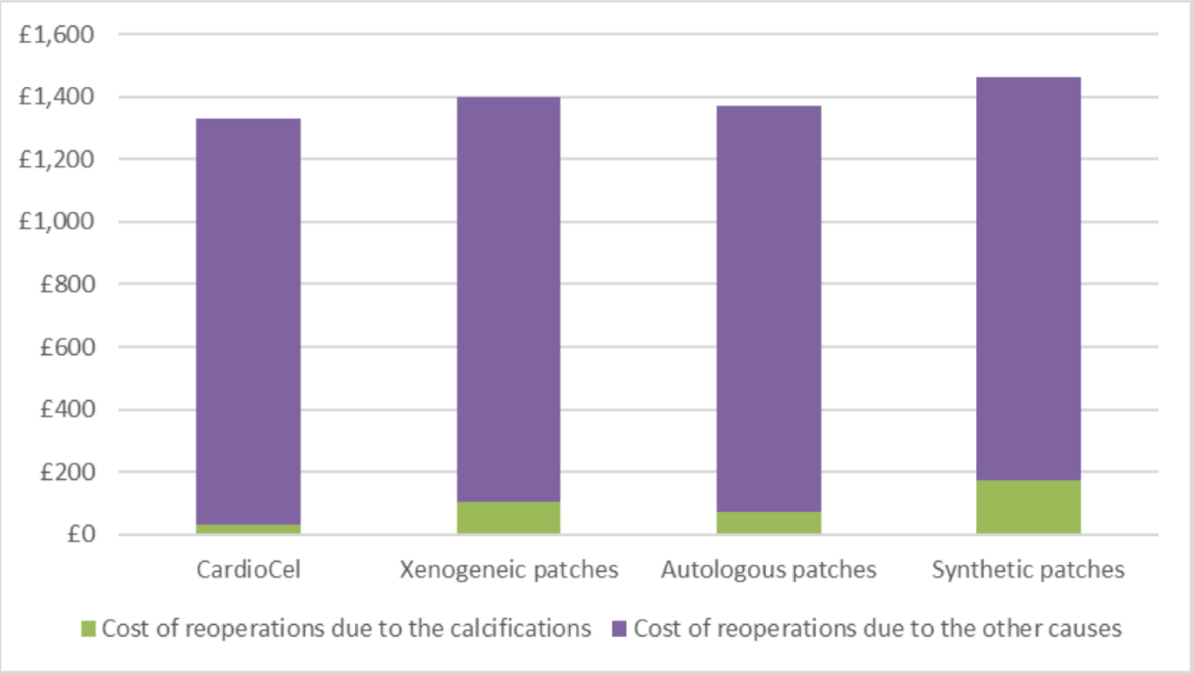

Fig AE Breakdown of cost of reoperations in the tetralogy of Fallot cohort

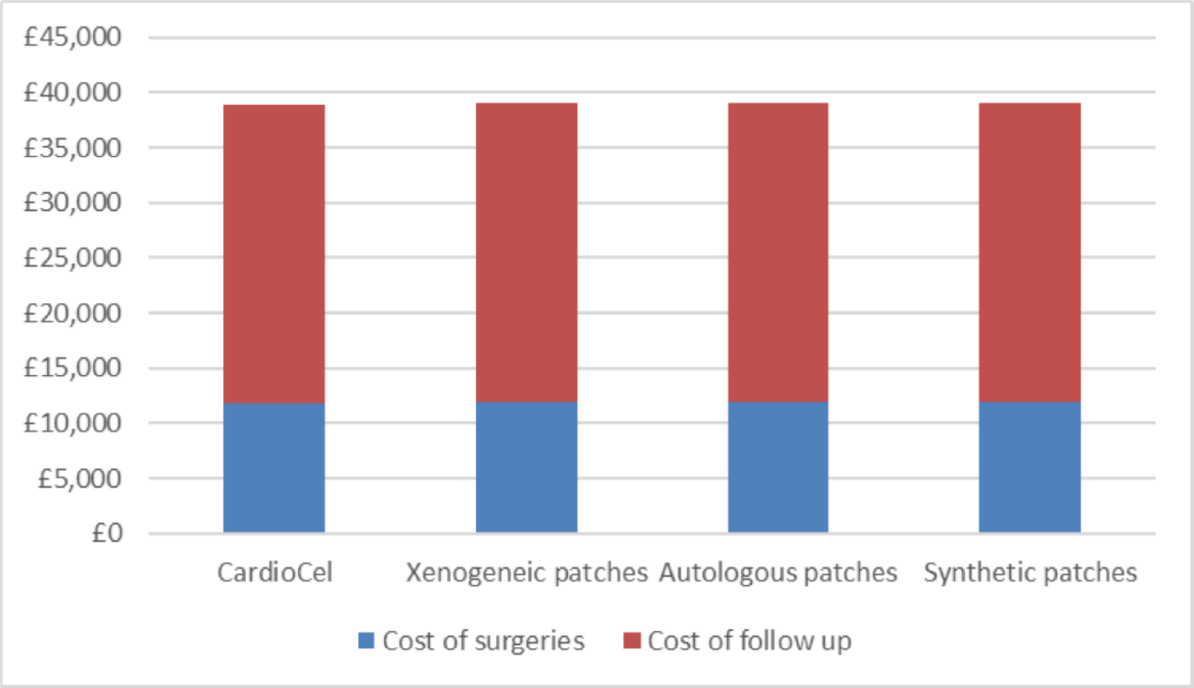

Fig AF Breakdown of cost of index surgery and follow-up in the tetralogy of Fallot cohort

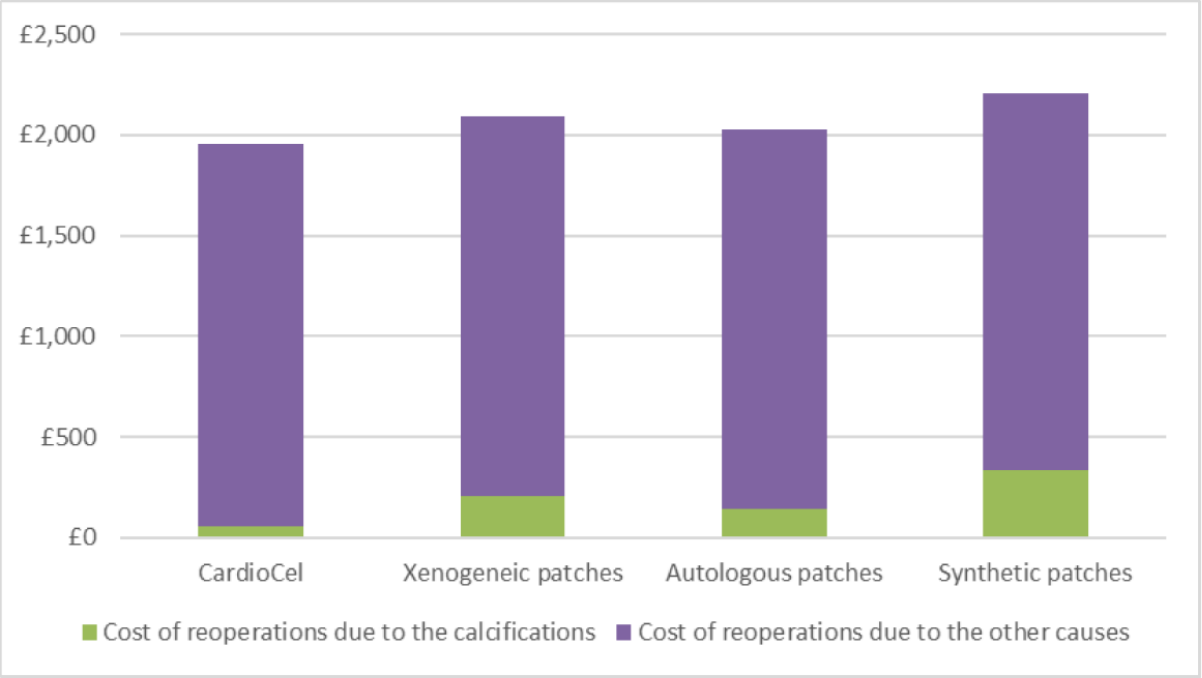

Fig AG Breakdown of cost of reoperations in the transposition of great arteries cohort

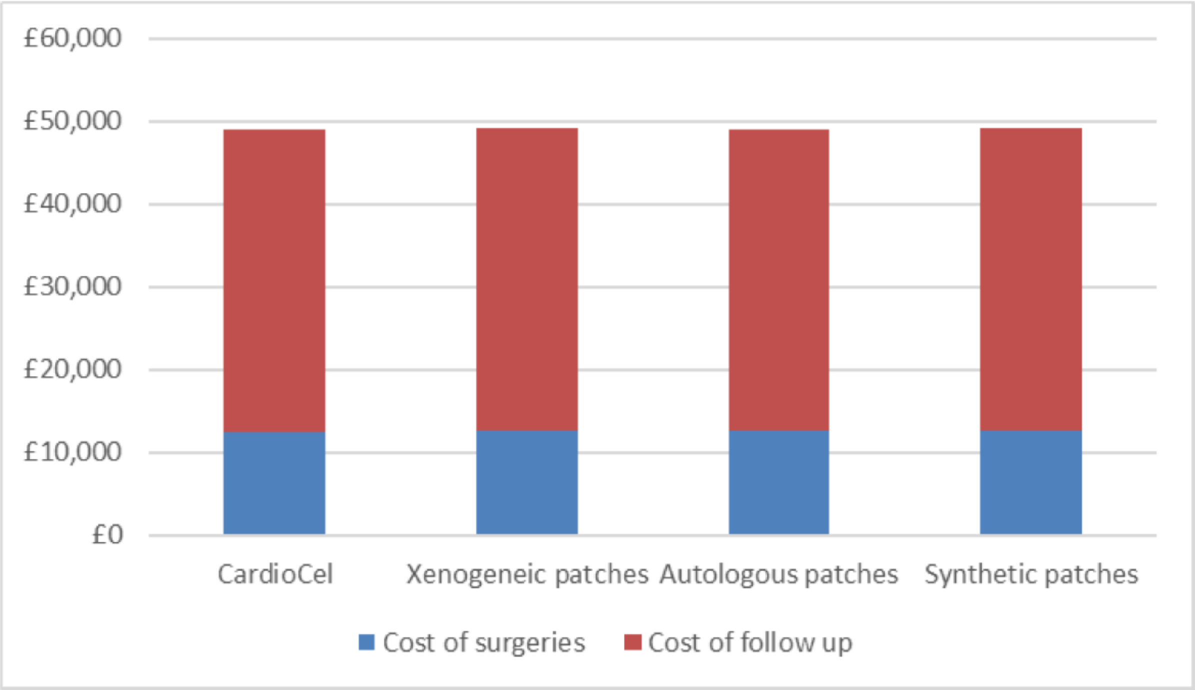

Fig AH Breakdown of cost of index surgery and follow-up in the transposition of great arteries cohort

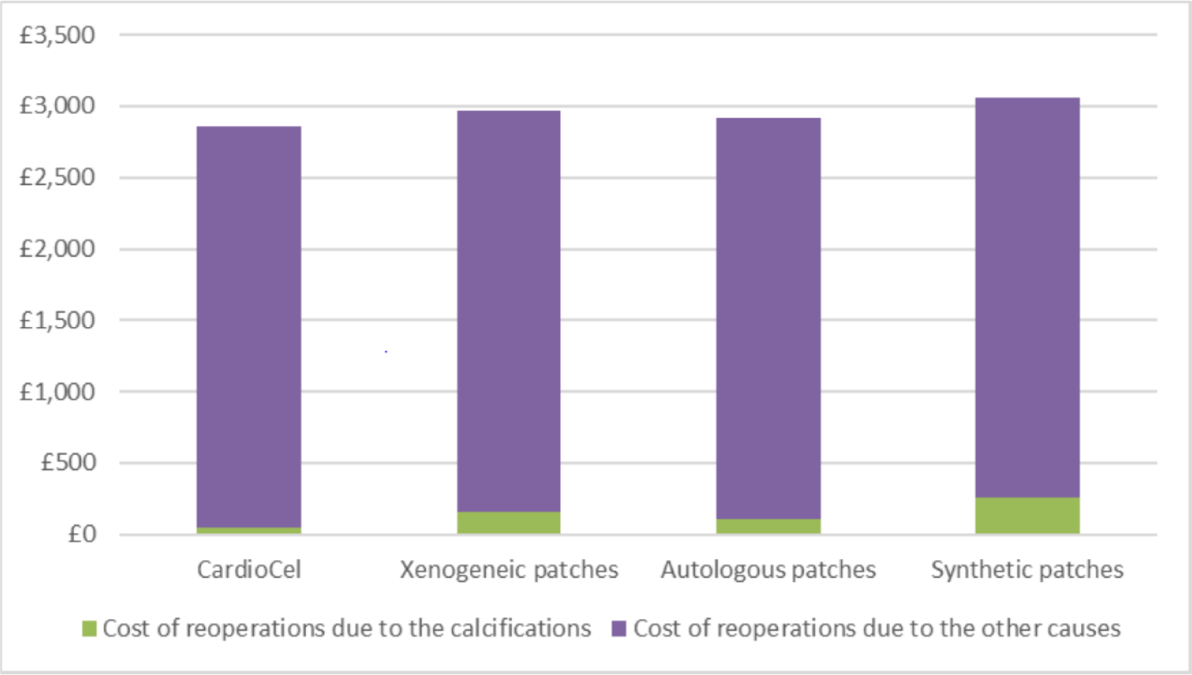

Fig AI Breakdown of cost of reoperations in the coarctation of the aorta cohort

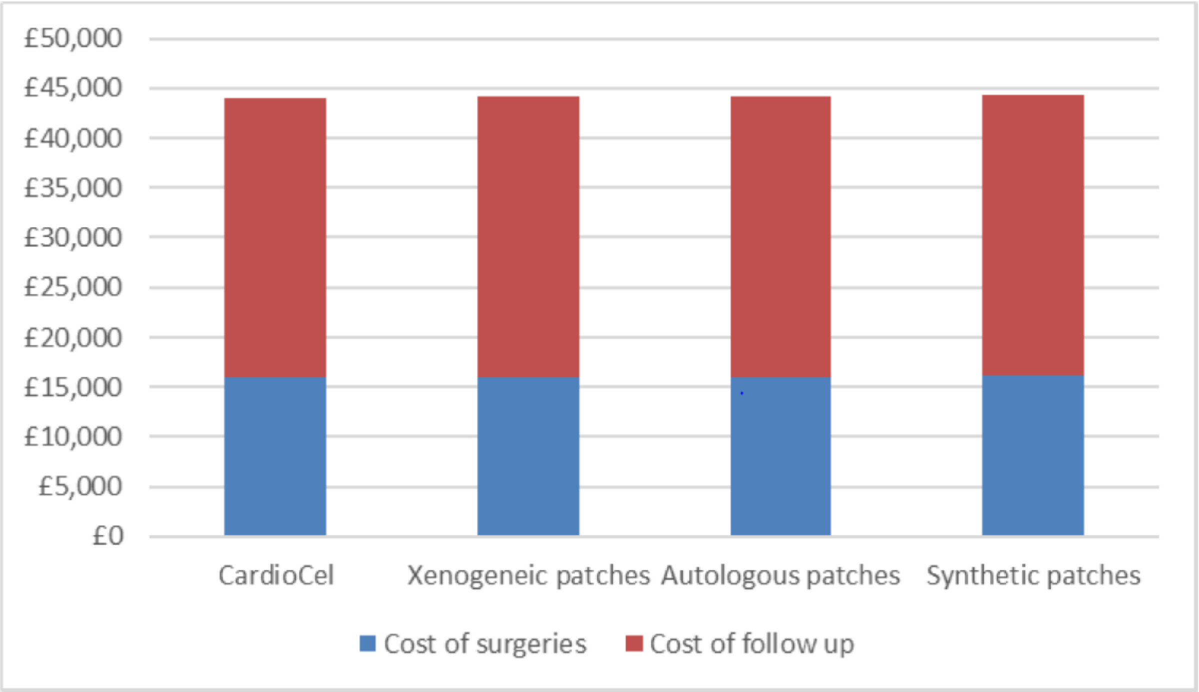

Fig AJ Breakdown of cost of index surgery and follow-up in the coarctation of the aorta cohort

Table L Results of the cost-effectiveness analysis for base-case CHD

**Patch**	**Cost, £**	**LY**	**QALY**	**Incremental**			**ICERs**
				**Cost, £**	**LY**	**QALY**	
CardioCel	27434	33.981	24.895	-	-	-	• Dominating
Autologous patches	27502	33.978	24.892	-68	0.003	0.002	• Dominated by CardioCel<br>• Dominating xenogeneic and synthetic patches
Xenogeneic patches	27532	33.976	24.891	-30	0.002	0.001	• Dominated by autologous patches/Dominating synthetic patches
Synthetic patches	27594	33.973	24.889	-62	0.003	0.002	• Dominated by xenogeneic patches

Legend: LY – life years. QALY – quality adjusted life years, ICER – incremental cost-effectiveness ratio.
