# Supplementary material for: Congenital heart defect repair with ADAPT tissue engineered pericardium scaffold: An early-stage health economic model
Source: PLoS One. 2018 Sep 27;13(9):e0204643. doi: 10.1371/journal.pone.0204643 (PMC6160133; doi:10.1371/journal.pone.0204643)
Supplement: S5 File — (PDF) [file pone.0204643.s005.pdf]

Table A Disease specific variables distributions and parameters used for the uncertainty analysis for the aortic valve stenosis

Variables	Det	Min	Max	P1	P2	Sample	Distribution
Mean age at index surgery	3.00	2.40	3.60	3.00	0.30	12	Normal
Patch related reoperation fraction	0.16	0.13	0.19	0.13	0.19	-	Triangular
Synthetic patch index surgery fraction	0.6	0.2	0.9	123	78	201	Beta
Xenogeneic patches index surgery fraction	0.1	0.0	0.3	18	183	201	Beta
Autologous patches index surgery fraction	0.3	0.1	0.6	59	142	201	Beta
Short term (30 day) operative mortality	0.1	0.0	0.1	232	3083	26519	Beta
Short-term (monthly) disutility of surgical procedure	-0.5	-0.4	-0.6	100	0	-	Gamma
Cost of index surgery	11158	8926	13390	16	697	-	Gamma
Cost of reoperation	13666	10933	16400	16	854	-	Gamma

Legend: Det. – Deterministic, Min. – Minimum, Max. – Maximum, Distr. – Distribution, P1/P2 – Parameters 1/2 (Normal and Lognormal distribution – P1: Mean, P2: Standard deviation; Gamma distribution – P1: Shape, P2: Scale; Beta – P1: Shape (α), P2: Shape (β); Triangular - P1: Minimum; P2: Maximum)

Table B Disease specific variables distributions and parameters used for the uncertainty analysis for the AVSD

Variables	Det	Min	Max	P1	P2	Sample	Distribution
Mean age at index surgery	7.00	5.60	8.40	7.00	0.70	167	Normal
Patch related reoperation fraction	0.90	0.86	0.94	198	22	220	Beta
Synthetic patch index surgery fraction	0.69	0.53	0.76	154	70	224	Beta
Xenogeneic patches index surgery fraction	0.16	0.12	0.23	35	189	224	Beta
Autologous patches index surgery fraction	0.16	0.12	0.23	35	189	224	Beta
Short term (30 day) operative mortality	0.02	0.01	0.03	73	3242	26519	Beta
Short-term (monthly) disutility of surgical procedure	-0.53	-0.42	-0.64	100	0	-	Gamma
Cost of index surgery	10537	8430	12644	16	659	-	Gamma
Cost of reoperation	13235	10588	15883	16	827	-	Gamma

Legend: AVSD - atrioventricular septal defect; Det. – Deterministic, Min. – Minimum, Max. – Maximum, Distr. – Distribution, P1/P2 – Parameters 1/2 (Normal and Lognormal distribution – P1: Mean, P2: Standard deviation; Gamma distribution – P1: Shape, P2: Scale; Beta – P1: Shape (α), P2: Shape (β); Triangular - P1: Minimum; P2: Maximum)

Table C Disease specific variables distributions and parameters used for the uncertainty analysis for the VSD

Variables	Det	Min	Max	P1	P2	Sample	Distribution
Mean age at index surgery	8.00	6.40	9.60	8.00	0.80	673	Normal
Patch related reoperation fraction	0.16	0.04	0.27	6	29	35	Beta
Synthetic patch index surgery fraction	0.53	0.37	0.68	205	178	383	Beta
Xenogeneic patches index surgery fraction	0.23	0.16	0.31	89	294	383	Beta
Autologous patches index surgery fraction	0.23	0.16	0.31	89	294	383	Beta
Short term (30 day) operative mortality	0.01	0.01	0.01	28	3287	26519	Beta
Short-term (monthly) disutility of surgical procedure	-0.35	-0.28	-0.42	100	0	-	Gamma
Cost of index surgery	5968	4774	7161	16	373	-	Gamma
Cost of reoperation	5968	4774	7161	16	373	-	Gamma

Legend: VSD - ventricular septal defect; Det. – Deterministic, Min. – Minimum, Max. – Maximum, Distr. – Distribution, P1/P2 – Parameters 1/2 (Normal and Lognormal distribution – P1: Mean, P2: Standard deviation; Gamma distribution – P1: Shape, P2: Scale; Beta – P1: Shape (α), P2: Shape (β); Triangular - P1: Minimum; P2: Maximum)

Table D Disease specific variables distributions and parameters used for the uncertainty analysis for the ToF

**Variables**	**Det**	**Min**	**Max**	**P1**	**P2**	**Sample**	**Distribution**
Mean age at index surgery	1.00	0.80	1.20	1.00	0.10	192	Normal
Patch related reoperation fraction	0.41	0.33	0.50	66	94	160	Beta
Synthetic patch index surgery fraction	0.01	0.01	0.01	2	158	160	Beta
Xenogeneic patches index surgery fraction	0.54	0.43	0.65	87	73	160	Beta
Autologous patches index surgery fraction	0.45	0.36	0.54	72	88	160	Beta
Short term (30 day) operative mortality	0.02	0.01	0.02	433	26086	26519	Beta
Short-term (monthly) disutility of surgical procedure	-0.47	-0.38	-0.56	100	0	-	Gamma
Cost of index surgery	10537	8430	12644	16	659	-	Gamma
Cost of reoperation	13235	10588	15883	16	827	-	Gamma

Legend: ToF - tetralogy of Fallot; Det. – Deterministic, Min. – Minimum, Max. – Maximum, Distr. – Distribution, P1/P2 – Parameters 1/2 (Normal and Lognormal distribution – P1: Mean, P2: Standard deviation; Gamma distribution – P1: Shape, P2: Scale; Beta – P1: Shape (α), P2: Shape (β); Triangular - P1: Minimum; P2: Maximum)

Table E Disease specific variables distributions and parameters used for the uncertainty analysis for the TGA

Variables	Det	Min	Max	P1	P2	Sample	Distribution
Mean age at index surgery	1.00	0.80	1.20	1.00	0.10	140	Normal
Patch related reoperation fraction	0.53	0.48	0.58	173	153	326	Beta
Synthetic patch index surgery fraction	0.37	0.30	0.56	125	210	335	Beta
Xenogeneic patches index surgery fraction	0.31	0.22	0.38	105	230	335	Beta
Autologous patches index surgery fraction	0.31	0.22	0.38	105	230	335	Beta
Short term (30 day) operative mortality	0.02	0.02	0.04	639	25880	26519	Beta
Short-term (monthly) disutility of surgical procedure	-0.59	-0.47	-0.71	100	0	-	Gamma
Cost of index surgery	10537	8429.6	12644.4	16	659	-	Gamma
Cost of reoperation	10537	8429.6	12644.4	16	659	-	Gamma

Legend: TGA – Transposition of great arteries; Det. – Deterministic, Min. – Minimum, Max. – Maximum, Distr. – Distribution, P1/P2 – Parameters 1/2 (Normal and Lognormal distribution – P1: Mean, P2: Standard deviation; Gamma distribution – P1: Shape, P2: Scale; Beta – P1: Shape (α), P2: Shape (β); Triangular - P1: Minimum; P2: Maximum)

Table F Disease specific variables distributions and parameters used for the uncertainty analysis for the CoA

Variables	Det	Min	Max	P1	P2	Sample	Distribution
Mean age at index surgery	2.00	1.60	2.40	2.00	0.20	202	Normal
Patch related reoperation fraction	0.29	0.20	0.37	136	328	464	Beta
Synthetic patch index surgery fraction	0.37	0.30	0.56	125	210	335	Beta
Xenogeneic patches index surgery fraction	0.31	0.22	0.38	105	230	335	Beta
Autologous patches index surgery fraction	0.31	0.22	0.38	105	230	335	Beta
Short term (30 day) operative mortality	0.01	0.01	0.02	263	26256	26519	Beta
Short-term (monthly) disutility of surgical procedure	-0.35	-0.28	-0.42	100	0	-	Gamma
Cost of index surgery	13049	10439	15658	16	816	-	Gamma
Cost of reoperation	13049	10439	15658	16	816	-	Gamma

Legend: CoA - Coarctation of the aorta; Det. – Deterministic, Min. – Minimum, Max. – Maximum, Distr. – Distribution, P1/P2 – Parameters 1/2 (Normal and Lognormal distribution – P1: Mean, P2: Standard deviation; Gamma distribution – P1: Shape, P2: Scale; Beta – P1: Shape (α), P2: Shape (β); Triangular - P1: Minimum; P2: Maximum)

Table G Utility and cost variables distributions used for the uncertainty analysis for the main classes of CHD disease

Variables	Det	Min	Max	P1	P2	Sample	Distribution
**Utilities**							
Mild CHD disability for the child 0 to 25 years	0.85	0.68	1.00	243.10	42.90	286	Beta
Mild CHD disability for the child 26 to 45 years	0.83	0.67	1.00	53.38	10.62	64	Beta
Mild CHD disability for the child 46 to 65 years	0.70	0.56	0.84	44.61	19.39	64	Beta
Moderate CHD disability for the child 0 to 25 years	0.75	0.60	0.90	24.00	8.00	32	Beta
Moderate CHD disability for the child 26 to 45 years	0.53	0.42	0.64	33.98	30.02	64	Beta
Moderate CHD disability for the child 46 to 65 years	0.49	0.39	0.59	31.23	32.77	64	Beta
Severe CHD disability for the child 0 to 2 years	0.40	0.32	0.48	114	172	286	Beta
Severe CHD disability for the child 3 to 18 years	0.39	0.31	0.47	112	174	286	Beta
Severe CHD disability for the child 19 to 25 years	0.39	0.31	0.47	112	174	286	Beta
Severe CHD disability for the child 26 to 45 years	0.32	0.26	0.39	21	43	64	Beta
**Cost**							
Pediatric mild CHD disability (Annual cost)	993	795	1192	109	9	-	Gamma
Pediatric moderate CHD disability (Annual cost)	1368	1094	1641	128	11	-	Gamma
Pediatric severe CHD disability (Annual cost)	2033	1626	2440	74	28	-	Gamma

Adult mild CHD disability (Annual cost)	974	780	1169	105	9	-	Gamma
Adult moderate CHD disability (Annual cost)	1290	1032	1548	114	11	-	Gamma
Adult severe CHD disability (Annual cost)	1884	1507	2260	63	30	-	Gamma
Paediatric cardiology follow-up attendance	183	146	220	16	11	-	Gamma
Adult cardiology follow-up attendance	141	113	169	16	9	-	Gamma
Consultant cardiology cost (20 mins)	60	48	72	16	4	-	Gamma
Congenital disorders (regular admission)	361	289	433	16	23	-	Gamma
ECG	38	30	46	16	2	-	Gamma
Chest X-ray	16	13	19	16	1	-	Gamma
Exercise test	88	70	106	16	6	-	Gamma
MRI scan	235	188	282	16	15	-	Gamma
Echocardiogram (outpatient)	235	188	282	16	15	-	Gamma
Echocardiogram (inpatient)	2274	1819	2729	16	142	-	Gamma
Catheter ≤ 18 years	199	159	239	16	12	-	Gamma
Catheter ≥ 18 years	340	272	408	16	21	-	Gamma

Legend: CHD – Congenital heart defects, Det. – Deterministic, Min. – Minimum, Max. – Maximum, Distr. – Distribution, P1/P2 – Parameters 1/2 (Normal and Lognormal distribution – P1: Mean, P2: Standard deviation; Gamma distribution – P1: Shape, P2: Scale; Beta – P1: Shape (α), P2: Shape (β); Triangular - P1: Minimum; P2: Maximum)

Table H Drug cost variables distributions used for the uncertainty analysis

Variables	Det	Min	Max	P1	P2	Sample	Distribution
**Children (1 to 18)**							
Digoxin	0.70	0.56	0.84	16.00	0.04	-	Beta
Frusemide	12.07	9.66	14.48	16.00	0.75	-	Beta
Warfarin	1.47	1.18	1.76	16.00	0.09	-	Beta
Amioderone	1.33	1.06	1.60	16.00	0.08	-	Beta
Bisoprolol	1.68	1.34	2.02	16.00	0.11	-	Beta
Verapamil	1.11	0.89	1.33	16.00	0.07	-	Beta
Ramipiril	3.74	2.99	4.49	16.00	0.23	-	Beta
**Adults (18+)**							
Digoxin	1.04	0.83	1.25	16.00	0.07	-	Beta
Frusemide	0.62	0.50	0.60	16.00	0.04	-	Beta
Warfarin	1.47	1.18	1.41	16.00	0.09	-	Beta
Amioderone	2.43	1.94	2.33	16.00	0.15	-	Beta
Bisoprolol	1.68	1.34	1.61	16.00	0.11	-	Beta
Verapamil	0.62	0.50	0.60	16.00	0.04	-	Beta
Ramipiril	3.74	2.99	3.59	16.00	0.23	-	Beta

Legend: Det. – Deterministic, Min. – Minimum, Max. – Maximum, Distr. – Distribution, P1/P2 – Parameters 1/2 (Normal and Lognormal distribution – P1: Mean, P2: Standard deviation; Gamma distribution – P1: Shape, P2: Scale; Beta – P1: Shape (α), P2: Shape (β); Uniform - P1: Minimum; P2: Maximum)

Table I Explanted patches fraction variables distributions and parameters used for the uncertainty analysis

**Variables**	**Det**	**Min**	**Max**	**P1**	**P2**	**Sample**	**Distribution**
Fraction of explanted synthetic patches due to the calcifications	0.3	0.21	0.39	5.4	12.6	18	Beta
Fraction of explanted xenogeneic patches due to the calcifications	0.182	0.1274	0.2366	4.186	18.814	23	Beta
Fraction of explanted autologous patches due to the calcifications	0.125	0.0875	0.1625	1	7	8	Beta
Fraction of explanted CardioCel due to the calcifications	0.05	0	0.1	0.45	8.55	9	Beta

Legend: Det. – Deterministic, Min. – Minimum, Max. – Maximum, Distr. – Distribution, P1/P2 – Parameters 1/2 (Normal and Lognormal distribution – P1: Mean, P2: Standard deviation; Gamma distribution – P1: Shape, P2: Scale; Beta – P1: Shape (α), P2: Shape (β); Triangular - P1: Minimum; P2: Maximum)
